# Bacterial community data associated with *Stephanitis pyri* using 16S rRNA gene metabarcoding

**DOI:** 10.1016/j.dib.2026.113107

**Published:** 2026-07-29

**Authors:** Mario Emilio Ernesto Franco, Óscar Crespo-Romo, Eva Edo-Tena, Ainara Peñalver-Cruz

**Affiliations:** a Department of Soil, Plant and Food Sciences, University of Bari Aldo Moro, Via Giovanni Amendola 165/A, 70126 Bari, Italy; b IRTA, Sustainable Plant Protection, Agrònoms, 25198 Lleida, Catalonia, Spain

**Keywords:** Pear lace bug, Bacteriome, Microbiota, Endosymbionts, Insect pests, Pome fruits, Tingidae

## Abstract

This data article reports the bacterial profile associated with the pear lace bug (*Stephanitis pyri*) collected from organic orchards in Catalonia, Spain, and reared under laboratory conditions. Total DNA from four composite samples, each comprising 30 individuals, along with an extraction control, was subjected to high-throughput amplicon sequencing targeting the V3-V4 region of the 16S rRNA gene on an Illumina NextSeq platform using a 2 × 300 bp paired-end configuration. The sequencing reads were processed with QIIME2 v2025.7, using a pre-trained SILVA classifier (2024–5) for the taxonomic assignment of amplicon sequence variants. Due to the fact that endosymbionts and other associated microorganisms can shape pest adaptation to new environments, this dataset is intended to serve as a valuable resource for evaluating changes in the microbiota of *S. pyri* in response to different conditions, thereby improving our understanding of its ecology and ultimately aiding the development of integrated management strategies.

Specifications Table

**Table:** 

Subject	Biology
Specific subject area	Insect pest bacterial communities.
Type of data	Raw 16S rRNA metabarcoding data.
Data collection	DNA was extracted from composite samples of *Stephanitis pyri* bodies using the SpeedTools Tissue DNA Extraction kit (Biotools, Spain). The V3-V4 variable regions of the 16S rRNA gene were sequenced on an Illumina NextSeq platform using primers 341F and 785R with 2 × 300 bp paired-end reads. Taxonomic analyses were performed with QIIME 2 version 2025.7.0.
Data source location	Insect rearing located in the Institute of Agrifood Research and Technology (IRTA), Av. Alcalde Rovira Roure 191, 25198 Lleida, Spain. Latitude and longitude: 41.62782° N, 0.59794° E.
Data accessibility	The sequencing data have been deposited in the NCBI Sequence Read Archive (SRA) under the accession numbers SRR38194417, SRR38194418, SRR38194419, SRR38194420, and SRR38194421, and are associated with the BioProject PRJNA1454935.
Related research article	None.

## 1 Value of the Data

• This study provides, as far as we know, the first characterization of the bacterial communities associated with *Stephanitis pyri*.
• This dataset can be used as a reference for assessing how different factors influence the bacteriome of the pear lace bug.
• The bacteriome of *S. pyri* could reveal new information about its ecological adaptation and defense mechanisms mediated by endosymbionts, thus providing valuable information for the development of integrated pest management strategies.

## 2 Background

The pear lace bug, *Stephanitis pyri* (Heteroptera: Tingidae), is a pest that primarily affects pear trees, but also other plants belonging to the Rosaceae family, such as apple trees [1]. This polyphagous insect feeds on leaves, causing a decrease in photosynthetic activity and respiration, thereby negatively impacting fruit yield [2]. Widely distributed in Mediterranean countries and the Palearctic region [1], the pear lace bug is being considered an emerging pest in organic fruit orchards in Catalonia, likely favored by the high temperatures and low relative humidity resulting from global warming, together with the withdrawal of synthetic pesticides [2].

Insects have co-evolved with symbiotic microorganisms that have become essential to their physiology and can play a decisive role in niche adaptation by allowing the host to exploit new food sources or to provide defense against pathogenic fungi and bacteria [[3], [4], [5]]. However, little is known about the microbial communities associated with *S. pyri*. Here, we present the first characterization of the bacterial community associated with the pear lace bug using high-throughput 16S rRNA gene amplicon sequencing. We expect this data to provide new insights into the ecology of this emerging pest and pave the way for the development of management strategies based on the microbiome.

## 3 Data Description

The dataset contains 16S rRNA metabarcoding sequencing reads derived from four composite biological samples of *Stephanitis pyri*, as well as an extraction control. The number of raw reads was 200,126 for sample 1, 200,158 for sample 2, 200,438 for sample 3, 107,674 for sample 4, and 69,120 for the extraction control. The non-template control (NTC) included during library preparation did not generate any usable sequencing reads, indicating the absence of detectable contamination during library preparation and sequencing. Quality assessment with FastQC showed high per-base sequence quality (Supplementary Material 1). Cutadapt v5.1 [6] detected that 97.9–99.2% of the reads contained the expected primer sequences, and adapter removal led to a reduction of 6–7% base pairs across all samples. Quality filtering, denoising, merging, and chimera removal with the Divisive Amplicon Denoising Algorithm 2 (DADA2) v.1.30.0 [7] resulted in 72.1–84.8% of reads as non-chimeric amplicon sequence variants (ASVs).

**Fig. 1:**
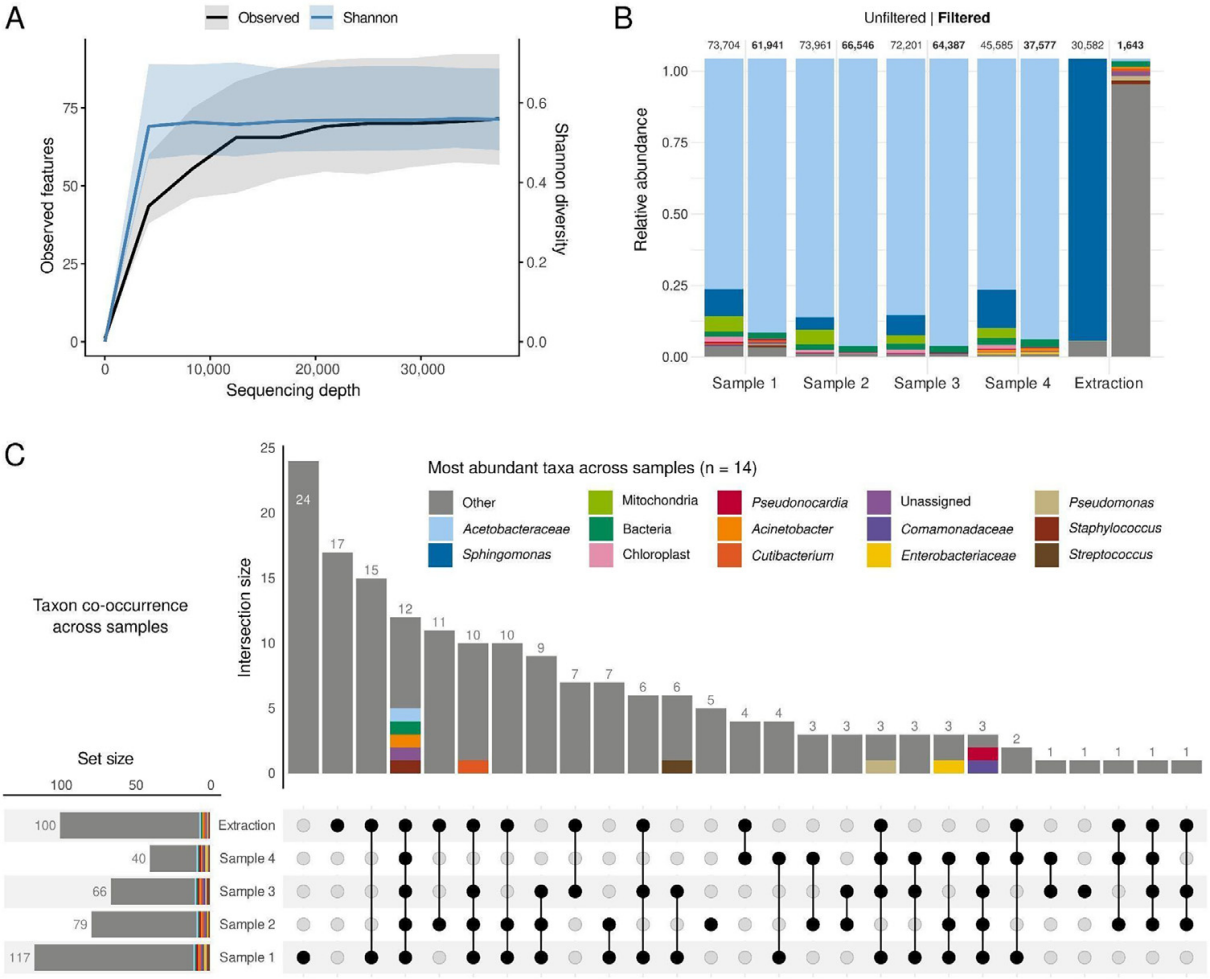
Overview of the bacterial community associated with *Stephanitis pyri*. (A) Alpha rarefaction curves based on Observed Features and Shannon diversity indices across sequencing depths indicate sufficient sampling depth to capture the bacterial diversity. (B) Relative abundance of taxa across samples and the extraction control before and after (in bold) removing non-target reads assigned to Mitochondria and Chloroplast, as well as the potential contaminant *Sphingomonas*. Numbers above the stacked bar charts correspond to read counts. (C) Intersection plot illustrating the taxa shared among samples after excluding Mitochondria, Chloroplast, and *Sphingomonas*. The 14 most abundant taxa across samples, excluding the extraction control, are shown.

Alpha rarefaction curves based on the ASVs and Shannon indices (Fig. 1A), as well as Faith’s phylogenetic diversity metric (Supplementary Material 2), confirmed that the sequencing depth was sufficient to capture the bacterial diversity across samples. Taxonomic assignment of ASVs revealed mitochondrial and chloroplast sequences in the *S. pyri* samples (Fig. 1B), which are possibly derived from plant material ingested by the insect. Notably, the genus *Sphingomonas* was detected in all *S. pyri* samples (10.0–17.6%) but also at high proportions in the extraction control (94.6%), which is consistent with a contamination introduced at some point of the experimental procedure. Consequently, ASVs assigned to mitochondria, chloroplasts, and *Sphingomonas* were excluded from the dataset, which left only 1,643 feature counts in the extraction control (Fig. 1B).

The whole-body-associated bacteriome of *S. pyri* was strongly dominated (91.9–96.3%) by *Acetobacteraceae* (Fig. 1B), which is a taxon that has been previously associated with insect guts [8,9]. It is worth noting that *Acetobacteraceae* was also detected in the extraction control (Fig. 1B, C) at very low abundance (15/1,643), yet its markedly higher relative abundance in *S. pyri* samples, together with the literature, suggests that this taxon is more likely a component of the host microbiome rather than background contamination. *Comamonadaceae* and *Pseudonocardia* were detected in all *S. pyri* samples and absent in the extraction control (Fig. 1C). The former family has also been reported as a member of insect gut microbiomes [9,10], whereas the latter genus is known as a cuticle-associated ant symbiont producing antimicrobial compounds involved in host defense [3]. *Enterobacteriaceae* ASVs were exclusively shared among three *S. pyri* samples (Fig. 1C) and are associated with insect gut microbiota as well [9,10]. After filtering ASVs with fewer than 10 total reads across all samples, the relatively high number of low-abundance taxa assigned at the genus level in the extraction control (*n* = 100, median 13 reads, IQR 8–19; range 4–120) may reflect the contribution of background contamination and low-frequency artifacts in a low-biomass sequencing context (Fig. 1B, C). QIIME2 output files, including ASV abundance tables, are available as Supplementary Material 2.

## 4 Experimental Design, Materials and Methods

### 4.1 Biosample collection and insect rearing

Insects were collected from pear orchards in Lleida (Catalonia, Spain) and maintained under laboratory conditions at the Entomology Laboratory of IRTA-Agrònoms (41.62782 N, 0.59794 E), Lleida, Spain. *Stephanitis pyri* rearing consisted of potted pear plants of 50 to 60 cm with insects of controlled age. Plants were watered weekly, and no fertilizers or chemical treatments were applied to these plants. On the day of the DNA extraction, 30 *S. pyri* adults less than a week old were collected per sample from the rearing colony and placed in 1.5 ml plastic tubes, after which the adults were frozen, and the pronota and wings of each insect were removed to avoid interference in the DNA extraction.

### 4.2 DNA extraction, PCR amplification, and 16S rRNA gene metabarcoding sequencing

Thirty *S. pyri* adults from the rearing colony were combined to generate each composite sample. Four composite samples were obtained from the rearing colony. To eliminate microorganisms on the insect’s surface, the samples were first disinfected with 75% (v/v) ethanol for 90 s and then rinsed three times with sterile double-distilled water. Total DNA extraction was performed using the SpeedTools Tissue DNA Extraction kit (Biotools, Spain). The quality of the obtained DNA was evaluated by absorbance using a microvolume spectrophotometer and on a 0.7% (w/v) agarose gel. Subsequently, an internal verification PCR was performed using 515F/926R to amplify the hypervariable V4-V5 region of the 16S rRNA, yielding amplicons of the expected size. Total DNA was sent to STAB-VIDA for amplification of the 16S rRNA V3-V4 hypervariable region using primers 341F (CCTACGGGNGGCWGCAG) and 785R (GACTACHVGGGTATCTAATCC), followed by library preparation including an NTC and an internally validated positive control and sequencing in a 2×300 bp paired-end approach on the Illumina NextSeq platform. An extraction control containing only reagents was also included and fully processed in parallel with the biological samples in order to monitor potential contamination throughout the whole process.

### 4.3 Bioinformatic analyses

Bioinformatics analysis was carried out using QIIME2 v2025.7 [11]. Sequence quality was evaluated and the parameters to be used in the successive read cleanup step were obtained (q2-demux). Primer and adapter removal was performed using Cutadapt v5.1 (q2-cutadapt) [6], and denoising and chimera removal with DADA2 v1.30.0 (q2-dada2) [7]. ASV representative sequences generated by DADA2 were aligned using MAFFT, and a phylogenetic tree was generated using FastTree (q2-phylogeny align-to-tree-mafft-fasttree). The resulting phylogenetic tree was midpoint-rooted and used to calculate Faith's Phylogenetic Diversity as part of the alpha diversity analysis (q2-diversity alpha-rarefaction). Taxonomy assignment to ASVs was performed using a pre-trained SILVA classifier (2024–5) (q2-feature-classifier) [12]. Potential contaminants or non-specific amplifications, such as mitochondria and chloroplasts, were removed together with low-frequency sequences observed fewer than ten times across all samples (q2-feature-table, q2-taxa). Potential contamination was additionally assessed using the prevalence method implemented in the *decontam* v1.28.0 package [13]; however, no ASVs were flagged as contaminants. Taxa abundance was visualized using bar charts and Krona plots [14]. QIIME2 and *decontam* scripts are provided as Supplementary Materials 3 and 4, respectively. Figures were created in RStudio 2024.12.1 (Build 563) using the *ggplot2* v.4.0.3 [15], *reshape2* v.1.4.5 [16], *dplyr* v.1.2.1 [17], and *ComplexUpset* v.1.3.3 [18] packages for R version 4.5.2 (2025-10-31), and further edited with Inkscape v1.2.2.

## Limitations

The deposited sequences correspond to raw sequencing reads and therefore contain non-target mitochondrial and chloroplast sequences, as well as potential contaminant sequences assigned to *Sphingomonas*. These reads should be filtered out prior to bacteriome analyses. Additionally, this dataset was obtained from laboratory-reared insects and should be interpreted accordingly, as their microbiome profiles may vary from those of insects collected in the field due to differences in diet and other environmental factors. Finally, it is important to note that the use of the V3-V4 region of the 16S rRNA gene and the SILVA database may not provide resolution at the genus level for certain taxa. Some bacteria identified in this study only at higher taxonomy levels (e.g., *Acetobacteraceae, Comamonadaceae*, and *Enterobacteriaceae*) require additional approaches to achieve higher taxonomic resolution.

## Ethics Statement

This study does not involve human subjects, vertebrate animal experiments, or any data collected from social media platforms.

## CRediT authorship contribution statement

**Mario Emilio Ernesto Franco:** Conceptualization, Data curation, Formal analysis, Investigation, Methodology, Software, Visualization, Writing – original draft, Writing – review & editing. **Óscar Crespo-Romo:** Methodology, Writing – review & editing. **Eva Edo-Tena:** Methodology, Writing – review & editing. **Ainara Peñalver-Cruz:** Conceptualization, Data curation, Formal analysis, Funding acquisition, Investigation, Methodology, Project administration, Resources, Supervision, Validation, Visualization, Writing – original draft, Writing – review & editing.

## Declaration of Competing Interest

The authors declare that they have no known competing financial interests or personal relationships that could have appeared to influence the work reported in this paper.

## Data Availability

SRABacterial Community Data Associated with Stephanitis pyri using 16S rRNA Gene Metabarcoding (Original data).

## Appendix Supplementary materials

Supplementary materials are present in the link https://doi.org/10.6084/m9.figshare.c.8428977 Image, application 1

Supplementary materials are present in the link https://doi.org/10.6084/m9.figshare.c.8428977 Image, application 2

Supplementary materials are present in the link https://doi.org/10.6084/m9.figshare.c.8428977 Image, application 3

Supplementary materials are present in the link https://doi.org/10.6084/m9.figshare.c.8428977 Image, application 4
