# Supplementary material for: Bacterial community data associated with Stephanitis pyri using 16S rRNA gene metabarcoding
Source: Data Brief. 2026 Jul 29;68:113107. doi: 10.1016/j.dib.2026.113107 (PMC13474393; doi:10.1016/j.dib.2026.113107)
Supplement: Supplementary file 2 [file mmc2.zip › supplementary_material_2/qiime2_results/baseline/exported_files/demux_visualization/overview.html]

q2\_demux : summarize


# Demultiplexed sequence counts summary

|  | forward reads | reverse reads |
| --- | --- | --- |
| Minimum | 34560 | 34560 |
| Median | 100063.0 | 100063.0 |
| Mean | 77751.6 | 77751.6 |
| Maximum | 100219 | 100219 |
| Total | 388758 | 388758 |

### Forward Reads Frequency Histogram

Download as PDF

### Reverse Reads Frequency Histogram

Download as PDF

# Per-sample sequence counts

#### Total Samples:  5 (forward)  5 (reverse)

|  | forward sequence count | reverse sequence count |
| --- | --- | --- |
| sample ID |  |  |
| Extr | 34560 | 34560 |
| c1 | 100063 | 100063 |
| c2 | 100079 | 100079 |
| c3 | 100219 | 100219 |
| c4 | 53837 | 53837 |

Download as TSV
