# Supplementary material for: Bacterial community data associated with Stephanitis pyri using 16S rRNA gene metabarcoding
Source: Data Brief. 2026 Jul 29;68:113107. doi: 10.1016/j.dib.2026.113107 (PMC13474393; doi:10.1016/j.dib.2026.113107)
Supplement: Supplementary file 2 [file mmc2.zip › supplementary_material_2/qiime2_results/baseline/exported_files/demux_visualization/quality-plot.html]

q2\_demux : summarize


Click and drag on plot to zoom in. Double click to zoom back out to full size.
Hover over a box to see the parametric seven-number summary of the quality scores at the corresponding position.

##### Forward Reads

##### Reverse Reads

# Demultiplexed sequence length summary

#### Forward Reads

|  |  |
| --- | --- |
| Total Sequences Sampled | 10000.0 |
| 2% | 301 nts |
| 9% | 301 nts |
| 25% | 301 nts |
| 50% (Median) | 301 nts |
| 75% | 301 nts |
| 91% | 301 nts |
| 98% | 301 nts |

#### Reverse Reads

|  |  |
| --- | --- |
| Total Sequences Sampled | 10000.0 |
| 2% | 301 nts |
| 9% | 301 nts |
| 25% | 301 nts |
| 50% (Median) | 301 nts |
| 75% | 301 nts |
| 91% | 301 nts |
| 98% | 301 nts |
