# Supplementary material for: Bacterial community data associated with Stephanitis pyri using 16S rRNA gene metabarcoding
Source: Data Brief. 2026 Jul 29;68:113107. doi: 10.1016/j.dib.2026.113107 (PMC13474393; doi:10.1016/j.dib.2026.113107)
Supplement: Supplementary file 2 [file mmc2.zip › supplementary_material_2/qiime2_results/baseline/exported_files/demux_visualization/demultiplex-summary-forward.pdf]

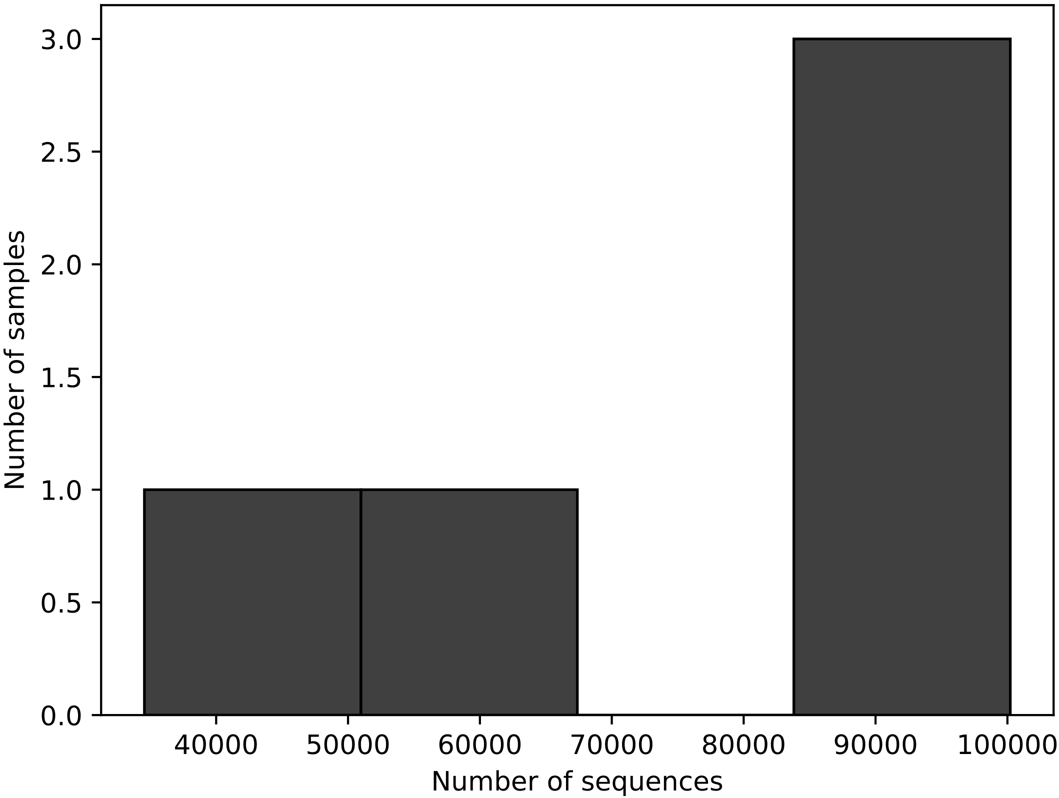
3.0
2.5
2.0
1.5
1.0
0.5
0.0
Number of samples
40000
50000
60000
70000
80000
90000
100000
Number of sequences
