# Supplementary material for: Bacterial community data associated with Stephanitis pyri using 16S rRNA gene metabarcoding
Source: Data Brief. 2026 Jul 29;68:113107. doi: 10.1016/j.dib.2026.113107 (PMC13474393; doi:10.1016/j.dib.2026.113107)
Supplement: Supplementary file 2 [file mmc2.zip › supplementary_material_2/qiime2_results/baseline/exported_files/table_summary/feature-frequency-detail.html]

q2\_feature\_table : summarize


- Overview
- Interactive Sample Detail
- Feature Detail

|  | Frequency | # of Samples Observed In |
| --- | --- | --- |
