# Supplementary material for: Bacterial community data associated with Stephanitis pyri using 16S rRNA gene metabarcoding
Source: Data Brief. 2026 Jul 29;68:113107. doi: 10.1016/j.dib.2026.113107 (PMC13474393; doi:10.1016/j.dib.2026.113107)
Supplement: Supplementary file 2 [file mmc2.zip › supplementary_material_2/qiime2_results/baseline/exported_files/table_summary/index.html]

q2\_feature\_table : summarize


- Overview
- Interactive Sample Detail
- Feature Detail

# Table summary

| Summary Statistic | Value |
| --- | --- |
| Number of samples |  |
| Number of unique features |  |
| Total observations (total frequency) |  |

# Frequency per sample

|  | Frequency |
| --- | --- |

Download as PDF

# Frequency per feature

|  | Frequency |
| --- | --- |

Download as PDF
