# Supplementary material for: Bacterial community data associated with Stephanitis pyri using 16S rRNA gene metabarcoding
Source: Data Brief. 2026 Jul 29;68:113107. doi: 10.1016/j.dib.2026.113107 (PMC13474393; doi:10.1016/j.dib.2026.113107)
Supplement: Supplementary file 2 [file mmc2.zip › supplementary_material_2/qiime2_results/baseline/exported_files/taxonomy_visualization/index.html]

q2\_metadata : tabulate


Download metadata TSV file

This file won't necessarily reflect dynamic sorting or filtering
options based on the interactive table below.
