# Supplementary material for: Bacterial community data associated with Stephanitis pyri using 16S rRNA gene metabarcoding
Source: Data Brief. 2026 Jul 29;68:113107. doi: 10.1016/j.dib.2026.113107 (PMC13474393; doi:10.1016/j.dib.2026.113107)
Supplement: Supplementary file 2 [file mmc2.zip › supplementary_material_2/qiime2_results/baseline/exported_files/krona/index.html]

Javascript must be enabled to view this page.

magnitude
magnitudeUnassigned

c1
c2
c3
c4
Extr

7404174098722934562630882

515000

515000

50000

50000

50000

50000

50000

015000

015000

015000

015000

015000

77608213025

77608213025

77608213025

77608213025

77608213025

77608213025

77608213025

210281014

00003

00003

00003

00003

00003

00003

4012611

401200

00400

00400

00400

00400

40000

40000

40000

40000

00800

00800

00800

00800

00005

00005

00005

00005

00005

00066

00066

00066

00066

00066

1701640

1701640

1701640

1701640

1701640

1701640

7393874023721834548630843

266905

266905

266905

266905

136905

136905

40000

40000

90000

90000

270440

270440

270440

270440

270440

80040

70400

120000

168000

168000

168000

130000

130000

130000

38000

38000

30000

08000

40000

40000

40000

40000

40000

40000

16154206

16154206

16154206

16154206

16154206

16154206

553017

000010

000010

000010

000010

000010

55307

55307

55307

55307

55307

123265886962331

50607

50607

50607

50607

50000

00607

122765886362324

00003

00003

00003

00003

122765886362321

122765886362321

122765886362321

122765886362321

000010

000010

000010

000010

000010

000010

5418351993

4311301971

360201933

360201933

1800129

80055

00070

100004

00600

00600

00600

00600

70835

00005

70830

1100019

80000

000019

30000

00040

00040

20005

20005

20000

20000

00005

00005

0410033

040011

040011

000011

04000

0010022

0010014

00008

001000

00006

00008

00008

50000

50000

50000

50000

07000

07000

07000

07000

00006

00006

00006

00006

00006

1175013

300013

300013

300013

000013

30000

87500

87500

87500

87000

00500

00003

00003

00003

00003

00003

125813781453103031

125813781453103031

125813781453103031

125813781453103031

125813781453103031

125813781453103031

1202325268213241

40000

40000

40000

40000

40000

1172307268206199

00005

00005

00005

00005

27000

20000

20000

20000

07000

07000

07000

080915

080915

080915

080915

270003

00003

00003

00003

120000

120000

120000

150000

150000

120000

30000

10235038

10235038

1011009

1011009

0125022

0125022

00007

00007

3021761651430

3021761651430

3021761651370

3021761651370

00060

00060

50000

50000

50000

50000

280020

280020

250020

70000

180020

30000

30000

14437473575

00608

00608

00608

1395016

04000

04000

850016

850016

50500

50500

160000

160000

160000

04000

04000

04000

1005133527

1005133527

1005133527

00009

00009

00009

151923015

130000

130000

21923015

21923015

405020

405020

405020

405020

4834820018

4301111014

428117014

428117011

00003

00400

00400

20000

20000

5337904

020000

020000

110000

110000

4217904

75004

3512900

1678261725

2120100

2120100

2120100

7661870

310000

310000

4161870

4161870

40000

40000

20000

20000

20000

6808025

190007

190007

4908018

150000

1508018

190000

440011

440011

240011

240011

24000

000011

20000

20000

20000

22140731

16110716

40070

40070

40070

750010

750010

70000

050010

56006

50000

50000

00006

00006

06000

06000

630015

630015

630015

03000

600015

100000

100000

100000

100000

100000

50000

50000

00006

00006

00006

00006

00006

00006

0140317

000017

000017

000017

000017

000017

014030

04000

04000

04000

04000

010030

010030

010030

010030

151610257

03600

03000

03000

03000

03000

00600

00600

00600

00600

874226

874226

07400

07400

00400

07000

00009

00009

00009

800217

800217

00020

800017

760012

50000

50000

50000

50000

200012

200012

200012

00006

00006

20000

06000

06000

06000

06000

000019

000019

000019

000019

000019

70000

70000

70000

70000

70000

70000

00600

00600

00600

00600

00600

00600

6891671154691614345929409

1040254155747254

86428573

86408568

5610050

5610050

9308051

9308051

70007

70007

1400010

1400010

02005

02005

02005

36152354

36152354

06000

06000

117404

00004

117400

70000

70000

02000

02000

1801950

1801950

6221300

6216300

100300

100300

5216000

5216000

05000

05000

05000

00005

00005

00005

00005

00037

00037

00037

00037

02300

02300

02000

02000

00300

00300

400014

400014

400014

40006

00008

03000

03000

03000

03000

11716230510

002310

002310

002310

81602745

050400

050400

81102340

81102340

00005

00005

1070005

1070005

1070005

20000

20000

20000

27042540937

144337811

500400

500400

94333811

340780

60326011

2560223126

2560223126

2560223126

00300

00300

00300

00300

08050

08050

08050

08050

000010

000010

000010

000010

3274041

3274027

03000

03000

3244027

0244027

30000

000014

000014

000014

442116841553

4300024

000012

000012

700012

700012

360000

360000

2611000

011000

011000

260000

260000

375000

170000

170000

05000

05000

200000

200000

522000

017000

017000

55000

55000

00005

00005

00005

3146877155

2576877150

2576877150

90000

90000

50000

50000

160000

160000

80000

80000

00005

00005

110000

110000

80000

80000

173000

03000

03000

170000

170000

02304

02304

02004

00300

050015

00006

00006

00004

00004

05005

00005

05000

00400

00400

00400

200000

200000

200000

30000

170000

6787670900690064271229155

000011

000011

000011

000011

30000

30000

30000

30000

3340013

3340013

334007

334007

00006

00006

5692264099620123532715

5692264099620123532715

5692264099620123532715

5692264099620123532715

83000

83000

83000

03000

80000

000017

000017

000017

000017

28226290127

270000

160000

160000

110000

40000

70000

188513054

190000

190000

000017

000017

2400018

2400018

14501308

14501308

05000

05000

000011

000011

465500

465500

465500

00008

00008

00008

04000

04000

04000

2159035

030025

030017

00008

2129010

2129010

03000

03000

03000

00200

00200

00200

000021

00007

00007

000014

00003

000011

04009

04000

04000

00009

00009

90000

90000

50000

50000

40000

40000

80000

80000

80000

80000

465604

465604

105600

105600

00004

00004

280000

280000

80000

80000

603012

603012

603012

603012

381836502008151810

381836502008151810

381836502008151810

381836502008151810

674131134948586728946

674131134948586728946

060023

060023

200000

200000

005013

005013

671831074943586728908

671831074943586728908

30002

30002

210811136173

210811136173

982545025

425200

425200

05000

260200

160000

5310400

4410400

40000

00400

50000

70000

20000

1910000

70000

90000

70000

20000

3102000

3102000

3102000

0019018

0019018

0019018

00007

00007

00007

00700

00700

00700

00700

12186220

00020

00020

00020

05006

05000

05000

00006

00006

00005

00005

00005

1213609

125009

125000

00009

08600

05600

03000

381432016

00700

00700

00700

70000

70000

70000

696016

40000

40000

20008

20000

00008

06600

06600

00008

00008

03000

03000

601000

601000

601000

195900

195900

195900

51623456

3600022

140000

140000

30006

30000

00006

1900016

1900016

15623028

40700

40700

20008

20008

46000

46000

0016020

0016020

50000

50000

00046

00046

00006

00040

11180056

6160056

20000

20000

00008

00008

00006

00006

05005

05005

4110023

4110023

000014

000014

52000

02000

02000

50000

50000

787225181112604

000011

000011

000011

000011

000011

10528281056

70000

70000

70000

70000

30000

30000

30000

30000

1515000

1515000

1515000

1515000

3838722

80000

80000

80000

2034718

60000

40000

20000

40000

40000

60040

60040

03000

03000

00009

00009

40030

40030

00400

00400

00009

00009

100400

40400

40400

20000

20000

40000

40000

00004

00004

00004

261020323

261020323

80000

80000

181020323

181020023

00030

160007

160007

20000

20000

40000

40000

30000

30000

50000

50000

20000

20000

00007

00007

00004

00004

00004

00004

203315599

171415577

171415577

171415577

17125548

0210029

3190022

3190022

3190022

3190022

1302200

1302200

1302200

1302200

1301600

00600

00004

00004

00004

00004

00004

00006

00006

00006

00006

00006

60114211093423

27885733182

003044

003044

003044

08000

08000

08000

27805433138

6664429120

00600

00008

00001

000012

041000

655282978

070021

000010

000010

1414648

1414648

00400

00400

70000

70000

30000

30000

30000

30000

2641110814

3056614

3006614

2406614

60000

05000

05000

150000

150000

150000

2080400

2080400

60000

480000

1540400

00020

00020

00020

06000

06000

06000

110000

90000

90000

20000

20000

2691861526

2691861520

2691861520

2691861520

00006

00006

00006

00003

00003

00003

00003

0351645

0351645

00006

00006

000812

000812

00020

00020

00003

00003

035624

03000

005624

00503

00503

00503

00503

38192721115

38192721115

005016

005016

1412101127

1412101127

1779766

1200032

579734

00300

00300

00003

00003

00033

00030

00003

70000

70000

030014

030014

030014

030014

000021

000021

000021

000021

214000

214000

30000

30000

30000

184000

184000

184000

06000

06000

06000

06000

06000

2312600

1512600

1512600

1512600

126600

06000

30000

80000

80000

80000

80000

40000

40000

40000

40000

40000

00045

00045

00045

00045

00005

00040

4100033

010007

00007

00007

00007

00007

010000

010000

010000

010000

400026

400026

400026

400026

400022

00004

97936053

78516017

78516017

140006

140006

00003

110003

30000

016007

016007

016007

00004

00004

00004

6435600

130000

90000

40000

5135600

510000

035600

20000

20000

20000

20000

20000

17420036

010006

010006

010006

010006

40000

40000

40000

40000

040020

040020

040020

040020

00005

00005

00005

00005

03000

03000

03000

03000

1325005

1325005

1325005

013000

70000

612005

196111534

6311512

6311512

001100

001100

001100

00050

00050

00050

630012

030012

030012

60000

60000

13301022

30065

00060

00060

00060

00005

00005

00005

30000

30000

30000

500417

000017

000017

000017

50040

50000

50000

00040

00040

50000

50000

50000

50000

03000

03000

03000

03000

080011

080011

080011

080011

080011

080011

110300

50000

50000

50000

50000

50000

60300

60300

00300

00300

00300

60000

60000

60000

1839012

03005

03005

03005

00005

00005

03000

03000

90907

90907

90907

90907

20000

70907

90000

90000

90000

90000

90000

40000

40000

40000

40000

40000

40000
