# Supplementary material for: Bacterial community data associated with Stephanitis pyri using 16S rRNA gene metabarcoding
Source: Data Brief. 2026 Jul 29;68:113107. doi: 10.1016/j.dib.2026.113107 (PMC13474393; doi:10.1016/j.dib.2026.113107)
Supplement: Supplementary file 2 [file mmc2.zip › supplementary_material_2/qiime2_results/filtered/exported_files/table_summary/feature-frequencies.pdf]

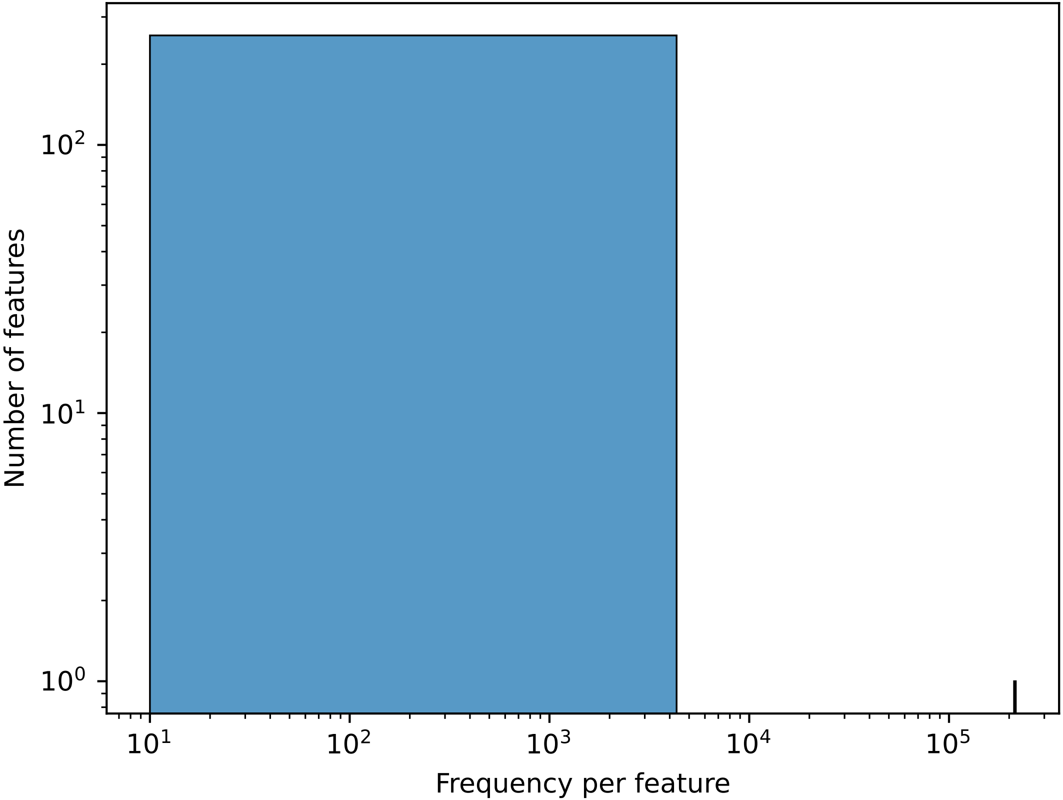

Number of features
10^2
10^1
10^0
10^1
10^2
10^3
10^4
10^5
Frequency per feature
