# Supplementary material for: Bacterial community data associated with Stephanitis pyri using 16S rRNA gene metabarcoding
Source: Data Brief. 2026 Jul 29;68:113107. doi: 10.1016/j.dib.2026.113107 (PMC13474393; doi:10.1016/j.dib.2026.113107)
Supplement: Supplementary file 2 [file mmc2.zip › supplementary_material_2/qiime2_results/filtered/exported_files/table_summary/sample-frequencies.pdf]

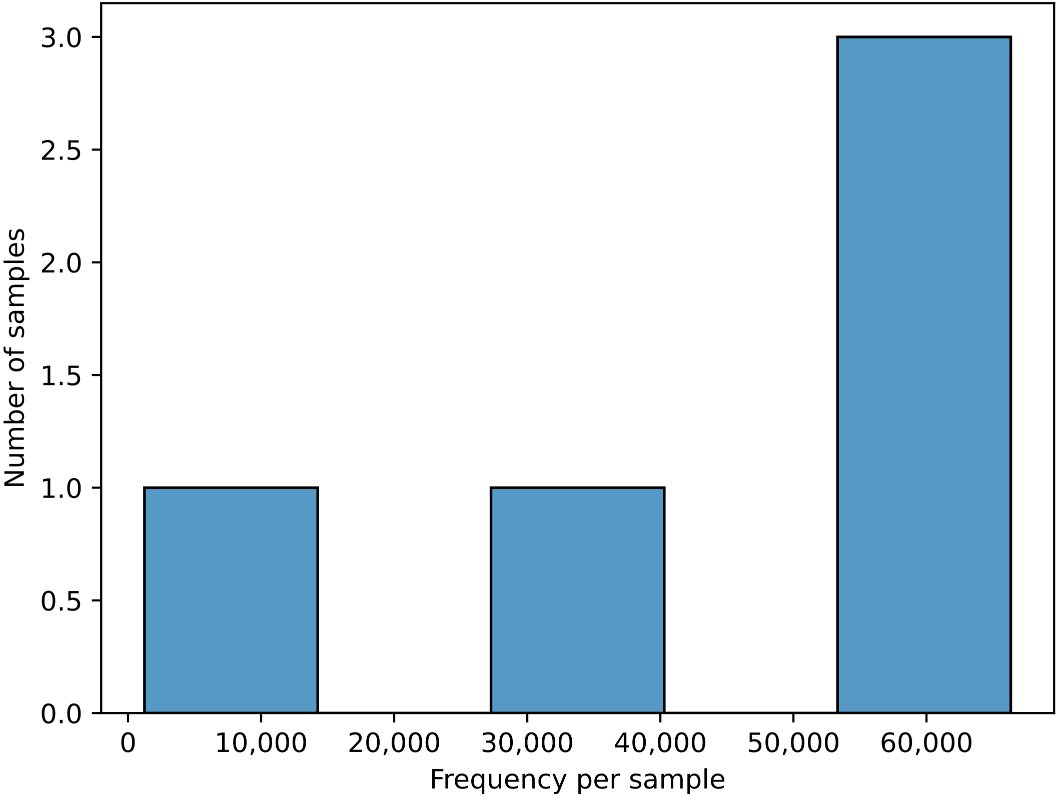

Number of samples
3.0
2.5
2.0
1.5
1.0
0.5
0.0
0
10,000
20,000
30,000
40,000
50,000
60,000
Frequency per sample
