# Supplementary material for: Bacterial community data associated with Stephanitis pyri using 16S rRNA gene metabarcoding
Source: Data Brief. 2026 Jul 29;68:113107. doi: 10.1016/j.dib.2026.113107 (PMC13474393; doi:10.1016/j.dib.2026.113107)
Supplement: Supplementary file 1 [file mmc1.zip › supplementary_material_1/fastqc/c1-N4059-16Sv3v4_S22_L001_R1_001_fastqc.zip!/c1-N4059-16Sv3v4_S22_L001_R1_001_fastqc/fastqc_report.html]

c1-N4059-16Sv3v4\_S22\_L001\_R1\_001.fastq.gz FastQC Report 

FastQC Report

Fri 7 Nov 2025  
c1-N4059-16Sv3v4\_S22\_L001\_R1\_001.fastq.gz

## Summary

- Basic Statistics
- Per base sequence quality
- Per tile sequence quality
- Per sequence quality scores
- Per base sequence content
- Per sequence GC content
- Per base N content
- Sequence Length Distribution
- Sequence Duplication Levels
- Overrepresented sequences
- Adapter Content

## Basic Statistics

| Measure | Value |
| --- | --- |
| Filename | c1-N4059-16Sv3v4\_S22\_L001\_R1\_001.fastq.gz |
| File type | Conventional base calls |
| Encoding | Sanger / Illumina 1.9 |
| Total Sequences | 100063 |
| Total Bases | 30.1 Mbp |
| Sequences flagged as poor quality | 0 |
| Sequence length | 301 |
| %GC | 55 |

## Per base sequence quality

## Per tile sequence quality

## Per sequence quality scores

## Per base sequence content

## Per sequence GC content

## Per base N content

## Sequence Length Distribution

## Sequence Duplication Levels

## Overrepresented sequences

| Sequence | Count | Percentage | Possible Source |
| --- | --- | --- | --- |
| CCTACGGGTGGCAGCAGTGGGGAATATTGGACAATGGGCGCAAGCCTGAT | 4419 | 4.416217782796838 | No Hit |
| TCCTACGGGTGGCAGCAGTGGGGAATATTGGACAATGGGCGCAAGCCTGA | 2049 | 2.0477099427360765 | No Hit |
| CCTACGGGAGGCAGCAGTGGGGAATATTGGACAATGGGCGCAAGCCTGAT | 1993 | 1.9917452005236702 | No Hit |
| CCTACGGGGGGCAGCAGTGGGGAATATTGGACAATGGGCGCAAGCCTGAT | 1735 | 1.7339076381879415 | No Hit |
| CCTACGGGTGGCTGCAGTGGGGAATATTGGACAATGGGCGCAAGCCTGAT | 1702 | 1.700928415098488 | No Hit |
| ACCTACGGGTGGCAGCAGTGGGGAATATTGGACAATGGGCGCAAGCCTGA | 1468 | 1.4670757422823621 | No Hit |
| CCTACGGGAGGCTGCAGTGGGGAATATTGGACAATGGGCGCAAGCCTGAT | 1183 | 1.1822551792370806 | No Hit |
| CCTACGGGCGGCAGCAGTGGGGAATATTGGACAATGGGCGCAAGCCTGAT | 1176 | 1.1752595864605297 | No Hit |
| GCCTACGGGTGGCAGCAGTGGGGAATATTGGACAATGGGCGCAAGCCTGA | 1106 | 1.1053036586950222 | No Hit |
| TCCTACGGGAGGCAGCAGTGGGGAATATTGGACAATGGGCGCAAGCCTGA | 1038 | 1.0373464717228145 | No Hit |
| CCCTACGGGTGGCAGCAGTGGGGAATATTGGACAATGGGCGCAAGCCTGA | 934 | 0.9334119504712031 | No Hit |
| TCCTACGGGTGGCTGCAGTGGGGAATATTGGACAATGGGCGCAAGCCTGA | 862 | 0.8614572819123952 | No Hit |
| TCCTACGGGGGGCAGCAGTGGGGAATATTGGACAATGGGCGCAAGCCTGA | 756 | 0.7555240198674835 | No Hit |
| CCTACGGGGGGCTGCAGTGGGGAATATTGGACAATGGGCGCAAGCCTGAT | 726 | 0.7255429079679802 | No Hit |
| TTCCTACGGGTGGCAGCAGTGGGGAATATTGGACAATGGGCGCAAGCCTG | 704 | 0.7035567592416777 | No Hit |
| GCCTACGGGAGGCAGCAGTGGGGAATATTGGACAATGGGCGCAAGCCTGA | 693 | 0.6925636848785265 | No Hit |
| ACCTACGGGAGGCAGCAGTGGGGAATATTGGACAATGGGCGCAAGCCTGA | 683 | 0.6825699809120255 | No Hit |
| TCCTACGGGAGGCTGCAGTGGGGAATATTGGACAATGGGCGCAAGCCTGA | 623 | 0.6226077571130189 | No Hit |
| ACCTACGGGGGGCAGCAGTGGGGAATATTGGACAATGGGCGCAAGCCTGA | 615 | 0.6146127939398179 | No Hit |
| ACCTACGGGTGGCTGCAGTGGGGAATATTGGACAATGGGCGCAAGCCTGA | 541 | 0.5406593845877097 | No Hit |
| TACCTACGGGTGGCAGCAGTGGGGAATATTGGACAATGGGCGCAAGCCTG | 531 | 0.5306656806212087 | No Hit |
| CCTACGGGTGGCAGCAGTGGGGAATATTGGACAATGGGCGAAAGCCTGAT | 528 | 0.5276675694312583 | No Hit |
| GCCTACGGGTGGCTGCAGTGGGGAATATTGGACAATGGGCGCAAGCCTGA | 487 | 0.48669338316860383 | No Hit |
| CCCTACGGGAGGCAGCAGTGGGGAATATTGGACAATGGGCGCAAGCCTGA | 463 | 0.46270849364900113 | No Hit |
| ATCCTACGGGTGGCAGCAGTGGGGAATATTGGACAATGGGCGCAAGCCTG | 461 | 0.4607097528557009 | No Hit |
| TCCTACGGGCGGCAGCAGTGGGGAATATTGGACAATGGGCGCAAGCCTGA | 454 | 0.4537141600791501 | No Hit |
| GCCTACGGGGGGCAGCAGTGGGGAATATTGGACAATGGGCGCAAGCCTGA | 422 | 0.4217343073863466 | No Hit |
| CCTACGGGCGGCTGCAGTGGGGAATATTGGACAATGGGCGCAAGCCTGAT | 415 | 0.41473871460979583 | No Hit |
| AACCTACGGGTGGCAGCAGTGGGGAATATTGGACAATGGGCGCAAGCCTG | 411 | 0.41074123302319543 | No Hit |
| ACCTACGGGAGGCTGCAGTGGGGAATATTGGACAATGGGCGCAAGCCTGA | 407 | 0.4067437514365949 | No Hit |
| CCCTACGGGTGGCTGCAGTGGGGAATATTGGACAATGGGCGCAAGCCTGA | 403 | 0.4027462698499945 | No Hit |
| GTCCTACGGGTGGCAGCAGTGGGGAATATTGGACAATGGGCGCAAGCCTG | 388 | 0.3877557139002428 | No Hit |
| TGCCTACGGGTGGCAGCAGTGGGGAATATTGGACAATGGGCGCAAGCCTG | 358 | 0.3577746020007395 | No Hit |
| ACCTACGGGCGGCAGCAGTGGGGAATATTGGACAATGGGCGCAAGCCTGA | 352 | 0.35177837962083885 | No Hit |
| TTCCTACGGGAGGCAGCAGTGGGGAATATTGGACAATGGGCGCAAGCCTG | 352 | 0.35177837962083885 | No Hit |
| TCCCTACGGGTGGCAGCAGTGGGGAATATTGGACAATGGGCGCAAGCCTG | 348 | 0.34778089803423845 | No Hit |
| GCCTACGGGAGGCTGCAGTGGGGAATATTGGACAATGGGCGCAAGCCTGA | 328 | 0.3277934901012362 | No Hit |
| CTCCTACGGGTGGCAGCAGTGGGGAATATTGGACAATGGGCGCAAGCCTG | 319 | 0.31879915653138524 | No Hit |
| TCCTACGGGGGGCTGCAGTGGGGAATATTGGACAATGGGCGCAAGCCTGA | 315 | 0.3148016749447848 | No Hit |
| CCTACGGGTGGCAGCAGTGGGGAATCTTGGACAATGGGCGAAAGCCCGAT | 306 | 0.30580734137493376 | No Hit |
| TTCCTACGGGGGGCAGCAGTGGGGAATATTGGACAATGGGCGCAAGCCTG | 301 | 0.3008104893916832 | No Hit |
| AGCCTACGGGTGGCAGCAGTGGGGAATATTGGACAATGGGCGCAAGCCTG | 293 | 0.2928155262184824 | No Hit |
| CCCTACGGGGGGCAGCAGTGGGGAATATTGGACAATGGGCGCAAGCCTGA | 287 | 0.2868193038385817 | No Hit |
| GACCTACGGGTGGCAGCAGTGGGGAATATTGGACAATGGGCGCAAGCCTG | 286 | 0.2858199334419316 | No Hit |
| TACCTACGGGAGGCAGCAGTGGGGAATATTGGACAATGGGCGCAAGCCTG | 270 | 0.26983000709552984 | No Hit |
| CACCTACGGGTGGCAGCAGTGGGGAATATTGGACAATGGGCGCAAGCCTG | 267 | 0.2668318959055795 | No Hit |
| TTCCTACGGGTGGCTGCAGTGGGGAATATTGGACAATGGGCGCAAGCCTG | 264 | 0.2638337847156291 | No Hit |
| ACCCTACGGGTGGCAGCAGTGGGGAATATTGGACAATGGGCGCAAGCCTG | 262 | 0.2618350439223289 | No Hit |
| ACCTACGGGGGGCTGCAGTGGGGAATATTGGACAATGGGCGCAAGCCTGA | 255 | 0.25483945114577816 | No Hit |
| GGCCTACGGGTGGCAGCAGTGGGGAATATTGGACAATGGGCGCAAGCCTG | 245 | 0.24484574717927704 | No Hit |
| CCTACGGGAGGCAGCAGTGGGGAATATTGGACAATGGGCGAAAGCCTGAT | 241 | 0.2408482655926766 | No Hit |
| CCTACGGGTGGCTGCAGTGGGGAATATTGGACAATGGGCGAAAGCCTGAT | 239 | 0.2388495247993764 | No Hit |
| GCCCTACGGGTGGCAGCAGTGGGGAATATTGGACAATGGGCGCAAGCCTG | 233 | 0.23285330241947572 | No Hit |
| CCCTACGGGAGGCTGCAGTGGGGAATATTGGACAATGGGCGCAAGCCTGA | 231 | 0.23085456162617551 | No Hit |
| CCTACGGGGGGCAGCAGTGGGGAATATTGGACAATGGGCGAAAGCCTGAT | 230 | 0.22985519122952539 | No Hit |
| TCCTACGGGTGGCAGCAGTGGGGAATATTGGACAATGGGCGAAAGCCTGA | 222 | 0.2218602280563245 | No Hit |
| GCCTACGGGCGGCAGCAGTGGGGAATATTGGACAATGGGCGCAAGCCTGA | 220 | 0.2198614872630243 | No Hit |
| TTTCCTACGGGTGGCAGCAGTGGGGAATATTGGACAATGGGCGCAAGCCT | 218 | 0.21786274646972406 | No Hit |
| CCCTACGGGCGGCAGCAGTGGGGAATATTGGACAATGGGCGCAAGCCTGA | 210 | 0.20986778329652317 | No Hit |
| TACCTACGGGGGGCAGCAGTGGGGAATATTGGACAATGGGCGCAAGCCTG | 206 | 0.20587030170992277 | No Hit |
| AGCCTACGGGAGGCAGCAGTGGGGAATATTGGACAATGGGCGCAAGCCTG | 203 | 0.2028721905199724 | No Hit |
| GTCCTACGGGAGGCAGCAGTGGGGAATATTGGACAATGGGCGCAAGCCTG | 201 | 0.2008734497266722 | No Hit |
| TGCCTACGGGAGGCAGCAGTGGGGAATATTGGACAATGGGCGCAAGCCTG | 199 | 0.19887470893337195 | No Hit |
| ATCCTACGGGAGGCAGCAGTGGGGAATATTGGACAATGGGCGCAAGCCTG | 199 | 0.19887470893337195 | No Hit |
| TTCCTACGGGAGGCTGCAGTGGGGAATATTGGACAATGGGCGCAAGCCTG | 194 | 0.1938778569501214 | No Hit |
| CCTACGGGGGGCAGCAGTGGGGAATCTTGGACAATGGGCGAAAGCCCGAT | 194 | 0.1938778569501214 | No Hit |
| TACCTACGGGTGGCTGCAGTGGGGAATATTGGACAATGGGCGCAAGCCTG | 194 | 0.1938778569501214 | No Hit |
| TTCCTACGGGCGGCAGCAGTGGGGAATATTGGACAATGGGCGCAAGCCTG | 186 | 0.18588289377692055 | No Hit |
| ATCCTACGGGGGGCAGCAGTGGGGAATATTGGACAATGGGCGCAAGCCTG | 185 | 0.18488352338027042 | No Hit |
| AACCTACGGGAGGCAGCAGTGGGGAATATTGGACAATGGGCGCAAGCCTG | 185 | 0.18488352338027042 | No Hit |
| ATCCTACGGGTGGCTGCAGTGGGGAATATTGGACAATGGGCGCAAGCCTG | 172 | 0.171891708223819 | No Hit |
| CACCTACGGGAGGCAGCAGTGGGGAATATTGGACAATGGGCGCAAGCCTG | 170 | 0.16989296743051877 | No Hit |
| ATTCCTACGGGTGGCAGCAGTGGGGAATATTGGACAATGGGCGCAAGCCT | 165 | 0.16489611544726823 | No Hit |
| TCCCTACGGGAGGCAGCAGTGGGGAATATTGGACAATGGGCGCAAGCCTG | 165 | 0.16489611544726823 | No Hit |
| GCCTACGGGGGGCTGCAGTGGGGAATATTGGACAATGGGCGCAAGCCTGA | 165 | 0.16489611544726823 | No Hit |
| ACCTACGGGTGGCAGCAGTGGGGAATATTGGACAATGGGCGAAAGCCTGA | 164 | 0.1638967450506181 | No Hit |
| TACCTACGGGAGGCTGCAGTGGGGAATATTGGACAATGGGCGCAAGCCTG | 161 | 0.16089863386066777 | No Hit |
| GACCTACGGGAGGCAGCAGTGGGGAATATTGGACAATGGGCGCAAGCCTG | 155 | 0.1549024114807671 | No Hit |
| CCTACGGGAGGCTGCAGTGGGGAATATTGGACAATGGGCGAAAGCCTGAT | 155 | 0.1549024114807671 | No Hit |
| TATCCTACGGGTGGCAGCAGTGGGGAATATTGGACAATGGGCGCAAGCCT | 154 | 0.153903041084117 | No Hit |
| AACCTACGGGTGGCTGCAGTGGGGAATATTGGACAATGGGCGCAAGCCTG | 152 | 0.1519043002908168 | No Hit |
| GGCCTACGGGAGGCAGCAGTGGGGAATATTGGACAATGGGCGCAAGCCTG | 150 | 0.14990555949751658 | No Hit |
| CTCCTACGGGAGGCAGCAGTGGGGAATATTGGACAATGGGCGCAAGCCTG | 148 | 0.14790681870421635 | No Hit |
| GCCTACGGGTGGCAGCAGTGGGGAATATTGGACAATGGGCGAAAGCCTGA | 146 | 0.14590807791091612 | No Hit |
| TCCTACGGGTGGCAGCAGTGGGGAATCTTGGACAATGGGCGAAAGCCCGA | 146 | 0.14590807791091612 | No Hit |
| GTCCTACGGGTGGCTGCAGTGGGGAATATTGGACAATGGGCGCAAGCCTG | 145 | 0.14490870751426602 | No Hit |
| TTACCTACGGGTGGCAGCAGTGGGGAATATTGGACAATGGGCGCAAGCCT | 144 | 0.14390933711761592 | No Hit |
| TGCCTACGGGTGGCTGCAGTGGGGAATATTGGACAATGGGCGCAAGCCTG | 143 | 0.1429099667209658 | No Hit |
| ACCCTACGGGAGGCAGCAGTGGGGAATATTGGACAATGGGCGCAAGCCTG | 143 | 0.1429099667209658 | No Hit |
| TACCTACGGGCGGCAGCAGTGGGGAATATTGGACAATGGGCGCAAGCCTG | 142 | 0.14191059632431569 | No Hit |
| CCCCTACGGGTGGCAGCAGTGGGGAATATTGGACAATGGGCGCAAGCCTG | 140 | 0.13991185553101546 | No Hit |
| CCTACGGGCGGCAGCAGTGGGGAATATTGGACAATGGGCGAAAGCCTGAT | 138 | 0.13791311473771523 | No Hit |
| AACCTACGGGGGGCAGCAGTGGGGAATATTGGACAATGGGCGCAAGCCTG | 137 | 0.13691374434106512 | No Hit |
| ATCCTACGGGAGGCTGCAGTGGGGAATATTGGACAATGGGCGCAAGCCTG | 133 | 0.1329162627544647 | No Hit |
| TCCCTACGGGTGGCTGCAGTGGGGAATATTGGACAATGGGCGCAAGCCTG | 129 | 0.12891878116786426 | No Hit |
| CCCTACGGGTGGCAGCAGTGGGGAATATTGGACAATGGGCGAAAGCCTGA | 129 | 0.12891878116786426 | No Hit |
| GACCTACGGGGGGCAGCAGTGGGGAATATTGGACAATGGGCGCAAGCCTG | 129 | 0.12891878116786426 | No Hit |
| CCTACGGGTGGCTGCATTTGATACGTCTAGACTAGAGTGTGAGAGAGGGT | 129 | 0.12891878116786426 | No Hit |
| CGCCTACGGGTGGCAGCAGTGGGGAATATTGGACAATGGGCGCAAGCCTG | 128 | 0.12791941077121413 | No Hit |
| TCCTACGGGCGGCTGCAGTGGGGAATATTGGACAATGGGCGCAAGCCTGA | 128 | 0.12791941077121413 | No Hit |
| ATACCTACGGGTGGCAGCAGTGGGGAATATTGGACAATGGGCGCAAGCCT | 126 | 0.1259206699779139 | No Hit |
| TGCCTACGGGGGGCAGCAGTGGGGAATATTGGACAATGGGCGCAAGCCTG | 124 | 0.12392192918461369 | No Hit |
| GTCCTACGGGAGGCTGCAGTGGGGAATATTGGACAATGGGCGCAAGCCTG | 123 | 0.12292255878796358 | No Hit |
| TCCTACGGGAGGCAGCAGTGGGGAATATTGGACAATGGGCGAAAGCCTGA | 122 | 0.12192318839131346 | No Hit |
| GACCTACGGGTGGCTGCAGTGGGGAATATTGGACAATGGGCGCAAGCCTG | 122 | 0.12192318839131346 | No Hit |
| AGCCTACGGGTGGCTGCAGTGGGGAATATTGGACAATGGGCGCAAGCCTG | 122 | 0.12192318839131346 | No Hit |
| CCCTACGGGGGGCTGCAGTGGGGAATATTGGACAATGGGCGCAAGCCTGA | 121 | 0.12092381799466337 | No Hit |
| TGCCTACGGGAGGCTGCAGTGGGGAATATTGGACAATGGGCGCAAGCCTG | 119 | 0.11892507720136314 | No Hit |
| TAACCTACGGGTGGCAGCAGTGGGGAATATTGGACAATGGGCGCAAGCCT | 118 | 0.11792570680471304 | No Hit |
| TCCCTACGGGGGGCAGCAGTGGGGAATATTGGACAATGGGCGCAAGCCTG | 116 | 0.11592696601141282 | No Hit |
| GTCCTACGGGGGGCAGCAGTGGGGAATATTGGACAATGGGCGCAAGCCTG | 116 | 0.11592696601141282 | No Hit |
| ACCCTACGGGTGGCTGCAGTGGGGAATATTGGACAATGGGCGCAAGCCTG | 115 | 0.11492759561476269 | No Hit |
| CCTACGGGTGGCTGCAGTGGGGAATCTTGGACAATGGGCGAAAGCCCGAT | 114 | 0.11392822521811259 | No Hit |
| TTCCCTACGGGTGGCAGCAGTGGGGAATATTGGACAATGGGCGCAAGCCT | 114 | 0.11392822521811259 | No Hit |
| CTCCTACGGGGGGCAGCAGTGGGGAATATTGGACAATGGGCGCAAGCCTG | 114 | 0.11392822521811259 | No Hit |
| AACCTACGGGAGGCTGCAGTGGGGAATATTGGACAATGGGCGCAAGCCTG | 113 | 0.11292885482146248 | No Hit |
| CCTACGGGAGGCAGCAGTGGGGAATCTTGGACAATGGGCGAAAGCCCGAT | 113 | 0.11292885482146248 | No Hit |
| AATCCTACGGGTGGCAGCAGTGGGGAATATTGGACAATGGGCGCAAGCCT | 110 | 0.10993074363151215 | No Hit |
| AGCCTACGGGGGGCAGCAGTGGGGAATATTGGACAATGGGCGCAAGCCTG | 110 | 0.10993074363151215 | No Hit |
| CTCCTACGGGTGGCTGCAGTGGGGAATATTGGACAATGGGCGCAAGCCTG | 108 | 0.10793200283821193 | No Hit |
| TTTCCTACGGGAGGCAGCAGTGGGGAATATTGGACAATGGGCGCAAGCCT | 107 | 0.1069326324415618 | No Hit |
| ACCTACGGGCGGCTGCAGTGGGGAATATTGGACAATGGGCGCAAGCCTGA | 107 | 0.1069326324415618 | No Hit |
| GCCCTACGGGAGGCAGCAGTGGGGAATATTGGACAATGGGCGCAAGCCTG | 107 | 0.1069326324415618 | No Hit |
| GTTCCTACGGGTGGCAGCAGTGGGGAATATTGGACAATGGGCGCAAGCCT | 103 | 0.10293515085496138 | No Hit |
| ATCCCTACGGGTGGCAGCAGTGGGGAATATTGGACAATGGGCGCAAGCCT | 103 | 0.10293515085496138 | No Hit |
| TTCCTACGGGGGGCTGCAGTGGGGAATATTGGACAATGGGCGCAAGCCTG | 101 | 0.10093641006166117 | No Hit |

## Adapter Content

Produced by FastQC (version 0.12.1)
