# Supplementary material for: Bacterial community data associated with Stephanitis pyri using 16S rRNA gene metabarcoding
Source: Data Brief. 2026 Jul 29;68:113107. doi: 10.1016/j.dib.2026.113107 (PMC13474393; doi:10.1016/j.dib.2026.113107)
Supplement: Supplementary file 1 [file mmc1.zip › supplementary_material_1/fastqc/c1-N4059-16Sv3v4_S22_L001_R2_001_fastqc.zip!/c1-N4059-16Sv3v4_S22_L001_R2_001_fastqc/fastqc_report.html]

c1-N4059-16Sv3v4\_S22\_L001\_R2\_001.fastq.gz FastQC Report 

FastQC Report

Fri 7 Nov 2025  
c1-N4059-16Sv3v4\_S22\_L001\_R2\_001.fastq.gz

## Summary

- Basic Statistics
- Per base sequence quality
- Per tile sequence quality
- Per sequence quality scores
- Per base sequence content
- Per sequence GC content
- Per base N content
- Sequence Length Distribution
- Sequence Duplication Levels
- Overrepresented sequences
- Adapter Content

## Basic Statistics

| Measure | Value |
| --- | --- |
| Filename | c1-N4059-16Sv3v4\_S22\_L001\_R2\_001.fastq.gz |
| File type | Conventional base calls |
| Encoding | Sanger / Illumina 1.9 |
| Total Sequences | 100063 |
| Total Bases | 30.1 Mbp |
| Sequences flagged as poor quality | 0 |
| Sequence length | 301 |
| %GC | 54 |

## Per base sequence quality

## Per tile sequence quality

## Per sequence quality scores

## Per base sequence content

## Per sequence GC content

## Per base N content

## Sequence Length Distribution

## Sequence Duplication Levels

## Overrepresented sequences

| Sequence | Count | Percentage | Possible Source |
| --- | --- | --- | --- |
| GACTACTAGGGTATCTAATCCTGTTTGCTCCCCACGCTTTCGCGCCTCAG | 2432 | 2.430468804653069 | No Hit |
| GACTACTCGGGTATCTAATCCTGTTTGCTCCCCACGCTTTCGCGCCTCAG | 2069 | 2.0676973506690786 | No Hit |
| GACTACTGGGGTATCTAATCCTGTTTGCTCCCCACGCTTTCGCGCCTCAG | 1882 | 1.880815086495508 | No Hit |
| GACTACAAGGGTATCTAATCCTGTTTGCTCCCCACGCTTTCGCGCCTCAG | 1839 | 1.837842159439553 | No Hit |
| GACTACCAGGGTATCTAATCCTGTTTGCTCCCCACGCTTTCGCGCCTCAG | 1588 | 1.5870001898803756 | No Hit |
| GACTACACGGGTATCTAATCCTGTTTGCTCCCCACGCTTTCGCGCCTCAG | 1518 | 1.5170442621148676 | No Hit |
| GACTACAGGGGTATCTAATCCTGTTTGCTCCCCACGCTTTCGCGCCTCAG | 1314 | 1.313172701198245 | No Hit |
| GACTACCGGGGTATCTAATCCTGTTTGCTCCCCACGCTTTCGCGCCTCAG | 1183 | 1.1822551792370806 | No Hit |
| GACTACCCGGGTATCTAATCCTGTTTGCTCCCCACGCTTTCGCGCCTCAG | 1036 | 1.0353477309295145 | No Hit |
| TGACTACTAGGGTATCTAATCCTGTTTGCTCCCCACGCTTTCGCGCCTCA | 856 | 0.8554610595324944 | No Hit |
| TGACTACTCGGGTATCTAATCCTGTTTGCTCCCCACGCTTTCGCGCCTCA | 687 | 0.6865674624986259 | No Hit |
| TGACTACAAGGGTATCTAATCCTGTTTGCTCCCCACGCTTTCGCGCCTCA | 599 | 0.5986228675934162 | No Hit |
| AGACTACTAGGGTATCTAATCCTGTTTGCTCCCCACGCTTTCGCGCCTCA | 568 | 0.5676423852972627 | No Hit |
| TGACTACTGGGGTATCTAATCCTGTTTGCTCCCCACGCTTTCGCGCCTCA | 549 | 0.5486543477609106 | No Hit |
| TGACTACCAGGGTATCTAATCCTGTTTGCTCCCCACGCTTTCGCGCCTCA | 513 | 0.5126770134815066 | No Hit |
| CGACTACTAGGGTATCTAATCCTGTTTGCTCCCCACGCTTTCGCGCCTCA | 511 | 0.5106782726882064 | No Hit |
| GGACTACTAGGGTATCTAATCCTGTTTGCTCCCCACGCTTTCGCGCCTCA | 508 | 0.5076801614982561 | No Hit |
| AGACTACTCGGGTATCTAATCCTGTTTGCTCCCCACGCTTTCGCGCCTCA | 501 | 0.5006845687217053 | No Hit |
| AGACTACTGGGGTATCTAATCCTGTTTGCTCCCCACGCTTTCGCGCCTCA | 450 | 0.44971667849254976 | No Hit |
| TGACTACAGGGGTATCTAATCCTGTTTGCTCCCCACGCTTTCGCGCCTCA | 441 | 0.44072234492269874 | No Hit |
| GGACTACTCGGGTATCTAATCCTGTTTGCTCCCCACGCTTTCGCGCCTCA | 435 | 0.4347261225427981 | No Hit |
| GGACTACTGGGGTATCTAATCCTGTTTGCTCCCCACGCTTTCGCGCCTCA | 434 | 0.433726752146148 | No Hit |
| TGACTACACGGGTATCTAATCCTGTTTGCTCCCCACGCTTTCGCGCCTCA | 410 | 0.4097418626265453 | No Hit |
| AGACTACCAGGGTATCTAATCCTGTTTGCTCCCCACGCTTTCGCGCCTCA | 407 | 0.4067437514365949 | No Hit |
| GGACTACAAGGGTATCTAATCCTGTTTGCTCCCCACGCTTTCGCGCCTCA | 406 | 0.4057443810399448 | No Hit |
| TGACTACCGGGGTATCTAATCCTGTTTGCTCCCCACGCTTTCGCGCCTCA | 395 | 0.39475130667679365 | No Hit |
| AGACTACAAGGGTATCTAATCCTGTTTGCTCCCCACGCTTTCGCGCCTCA | 384 | 0.38375823231364237 | No Hit |
| GGACTACCAGGGTATCTAATCCTGTTTGCTCCCCACGCTTTCGCGCCTCA | 374 | 0.3737645283471413 | No Hit |
| CGACTACTGGGGTATCTAATCCTGTTTGCTCCCCACGCTTTCGCGCCTCA | 365 | 0.36477019477729034 | No Hit |
| GGACTACAGGGGTATCTAATCCTGTTTGCTCCCCACGCTTTCGCGCCTCA | 352 | 0.35177837962083885 | No Hit |
| CGACTACTCGGGTATCTAATCCTGTTTGCTCCCCACGCTTTCGCGCCTCA | 347 | 0.3467815276375883 | No Hit |
| TGACTACCCGGGTATCTAATCCTGTTTGCTCCCCACGCTTTCGCGCCTCA | 345 | 0.3447827868442881 | No Hit |
| CGACTACCAGGGTATCTAATCCTGTTTGCTCCCCACGCTTTCGCGCCTCA | 329 | 0.3287928604978863 | No Hit |
| CGACTACAAGGGTATCTAATCCTGTTTGCTCCCCACGCTTTCGCGCCTCA | 323 | 0.3227966381179857 | No Hit |
| GGACTACACGGGTATCTAATCCTGTTTGCTCCCCACGCTTTCGCGCCTCA | 316 | 0.3158010453414349 | No Hit |
| AGACTACACGGGTATCTAATCCTGTTTGCTCCCCACGCTTTCGCGCCTCA | 313 | 0.3128029341514846 | No Hit |
| GACTACTAGGGTATCTAATCCTGTTTGCTCCCCACGCTTTCGCACCTCAG | 303 | 0.30280923018498346 | No Hit |
| AGACTACAGGGGTATCTAATCCTGTTTGCTCCCCACGCTTTCGCGCCTCA | 285 | 0.28482056304528147 | No Hit |
| TTGACTACTAGGGTATCTAATCCTGTTTGCTCCCCACGCTTTCGCGCCTC | 280 | 0.2798237110620309 | No Hit |
| GACTACTCGGGTATCTAATCCTGTTTGCTCCCCACGCTTTCGCACCTCAG | 280 | 0.2798237110620309 | No Hit |
| AGACTACCCGGGTATCTAATCCTGTTTGCTCCCCACGCTTTCGCGCCTCA | 271 | 0.2708293774921799 | No Hit |
| AGACTACCGGGGTATCTAATCCTGTTTGCTCCCCACGCTTTCGCGCCTCA | 264 | 0.2638337847156291 | No Hit |
| GGACTACCGGGGTATCTAATCCTGTTTGCTCCCCACGCTTTCGCGCCTCA | 264 | 0.2638337847156291 | No Hit |
| CGACTACAGGGGTATCTAATCCTGTTTGCTCCCCACGCTTTCGCGCCTCA | 258 | 0.2578375623357285 | No Hit |
| GACTACTGGGGTATCTAATCCTGTTTGCTCCCCACGCTTTCGCACCTCAG | 254 | 0.25384008074912806 | No Hit |
| CGACTACACGGGTATCTAATCCTGTTTGCTCCCCACGCTTTCGCGCCTCA | 247 | 0.2468444879725773 | No Hit |
| GACTACAAGGGTATCTAATCCTGTTTGCTCCCCACGCTTTCGCACCTCAG | 246 | 0.24584511757592717 | No Hit |
| CGACTACCCGGGTATCTAATCCTGTTTGCTCCCCACGCTTTCGCGCCTCA | 238 | 0.23785015440272628 | No Hit |
| TTGACTACTCGGGTATCTAATCCTGTTTGCTCCCCACGCTTTCGCGCCTC | 218 | 0.21786274646972406 | No Hit |
| GACTACCAGGGTATCTAATCCTGTTTGCTCCCCACGCTTTCGCACCTCAG | 207 | 0.20686967210657284 | No Hit |
| GACTACACGGGTATCTAATCCTGTTTGCTCCCCACGCTTTCGCACCTCAG | 199 | 0.19887470893337195 | No Hit |
| GACTACAGGGGTATCTAATCCTGTTTGCTCCCCACGCTTTCGCACCTCAG | 190 | 0.18988037536352098 | No Hit |
| GACTACTAGGGTATCTAATCCCGTTCGCTCCCCATGCTTTCGCACTCCAG | 184 | 0.18388415298362032 | No Hit |
| ATGACTACTAGGGTATCTAATCCTGTTTGCTCCCCACGCTTTCGCGCCTC | 181 | 0.18088604179367 | No Hit |
| GTGACTACTAGGGTATCTAATCCTGTTTGCTCCCCACGCTTTCGCGCCTC | 179 | 0.17888730100036976 | No Hit |
| TTGACTACACGGGTATCTAATCCTGTTTGCTCCCCACGCTTTCGCGCCTC | 179 | 0.17888730100036976 | No Hit |
| TTGACTACTGGGGTATCTAATCCTGTTTGCTCCCCACGCTTTCGCGCCTC | 172 | 0.171891708223819 | No Hit |
| GGACTACCCGGGTATCTAATCCTGTTTGCTCCCCACGCTTTCGCGCCTCA | 170 | 0.16989296743051877 | No Hit |
| TGGACTACTCGGGTATCTAATCCTGTTTGCTCCCCACGCTTTCGCGCCTC | 168 | 0.16789422663721854 | No Hit |
| TGGACTACTAGGGTATCTAATCCTGTTTGCTCCCCACGCTTTCGCGCCTC | 166 | 0.16589548584391833 | No Hit |
| TTGACTACAGGGGTATCTAATCCTGTTTGCTCCCCACGCTTTCGCGCCTC | 166 | 0.16589548584391833 | No Hit |
| TAGACTACTAGGGTATCTAATCCTGTTTGCTCCCCACGCTTTCGCGCCTC | 166 | 0.16589548584391833 | No Hit |
| GACTACTAGGGTATCTAATCCTGTTTGCTCCCCACGCTTTCGCGNCTCAG | 165 | 0.16489611544726823 | No Hit |
| TTGACTACCAGGGTATCTAATCCTGTTTGCTCCCCACGCTTTCGCGCCTC | 165 | 0.16489611544726823 | No Hit |
| GACTACCGGGGTATCTAATCCTGTTTGCTCCCCACGCTTTCGCACCTCAG | 162 | 0.16189800425731787 | No Hit |
| TCGACTACTAGGGTATCTAATCCTGTTTGCTCCCCACGCTTTCGCGCCTC | 151 | 0.15090492989416668 | No Hit |
| TAGACTACTGGGGTATCTAATCCTGTTTGCTCCCCACGCTTTCGCGCCTC | 149 | 0.14890618910086645 | No Hit |
| AAGACTACTAGGGTATCTAATCCTGTTTGCTCCCCACGCTTTCGCGCCTC | 148 | 0.14790681870421635 | No Hit |
| CTGACTACTAGGGTATCTAATCCTGTTTGCTCCCCACGCTTTCGCGCCTC | 145 | 0.14490870751426602 | No Hit |
| ATGACTACTGGGGTATCTAATCCTGTTTGCTCCCCACGCTTTCGCGCCTC | 145 | 0.14490870751426602 | No Hit |
| CGACTACCGGGGTATCTAATCCTGTTTGCTCCCCACGCTTTCGCGCCTCA | 144 | 0.14390933711761592 | No Hit |
| TAGACTACAAGGGTATCTAATCCTGTTTGCTCCCCACGCTTTCGCGCCTC | 144 | 0.14390933711761592 | No Hit |
| GTGACTACTGGGGTATCTAATCCTGTTTGCTCCCCACGCTTTCGCGCCTC | 143 | 0.1429099667209658 | No Hit |
| GACTACTCGGGTATCTAATCCCGTTCGCTCCCCATGCTTTCGCACTCCAG | 142 | 0.14191059632431569 | No Hit |
| GACTACTGGGGTATCTAATCCCGTTCGCTCCCCATGCTTTCGCACTCCAG | 142 | 0.14191059632431569 | No Hit |
| GACTACTGGGGTATCTAATCCTGTTTGCTCCCCACGCTTTCGCGNCTCAG | 141 | 0.14091122592766556 | No Hit |
| TAGACTACTCGGGTATCTAATCCTGTTTGCTCCCCACGCTTTCGCGCCTC | 140 | 0.13991185553101546 | No Hit |
| GTGACTACAAGGGTATCTAATCCTGTTTGCTCCCCACGCTTTCGCGCCTC | 139 | 0.13891248513436535 | No Hit |
| ATGACTACTCGGGTATCTAATCCTGTTTGCTCCCCACGCTTTCGCGCCTC | 139 | 0.13891248513436535 | No Hit |
| ATGACTACAAGGGTATCTAATCCTGTTTGCTCCCCACGCTTTCGCGCCTC | 135 | 0.13491500354776492 | No Hit |
| TAGACTACCAGGGTATCTAATCCTGTTTGCTCCCCACGCTTTCGCGCCTC | 135 | 0.13491500354776492 | No Hit |
| GACTACAAGGGTATCTAATCCTGTTTGCTCCCCACGCTTTCGCGNCTCAG | 134 | 0.1339156331511148 | No Hit |
| GACTACTCGGGTATCTAATCCTGTTTGCTCCCCACGCTTTCGCGNCTCAG | 133 | 0.1329162627544647 | No Hit |
| TCGACTACAAGGGTATCTAATCCTGTTTGCTCCCCACGCTTTCGCGCCTC | 130 | 0.12991815156451436 | No Hit |
| GTGACTACTCGGGTATCTAATCCTGTTTGCTCCCCACGCTTTCGCGCCTC | 129 | 0.12891878116786426 | No Hit |
| TCGACTACTCGGGTATCTAATCCTGTTTGCTCCCCACGCTTTCGCGCCTC | 129 | 0.12891878116786426 | No Hit |
| ATGACTACAGGGGTATCTAATCCTGTTTGCTCCCCACGCTTTCGCGCCTC | 119 | 0.11892507720136314 | No Hit |
| TGGACTACAAGGGTATCTAATCCTGTTTGCTCCCCACGCTTTCGCGCCTC | 117 | 0.11692633640806292 | No Hit |
| TAGACTACAGGGGTATCTAATCCTGTTTGCTCCCCACGCTTTCGCGCCTC | 116 | 0.11592696601141282 | No Hit |
| CAGACTACTAGGGTATCTAATCCTGTTTGCTCCCCACGCTTTCGCGCCTC | 116 | 0.11592696601141282 | No Hit |
| TAGACTACACGGGTATCTAATCCTGTTTGCTCCCCACGCTTTCGCGCCTC | 116 | 0.11592696601141282 | No Hit |
| TGGACTACTGGGGTATCTAATCCTGTTTGCTCCCCACGCTTTCGCGCCTC | 115 | 0.11492759561476269 | No Hit |
| GACTACCCGGGTATCTAATCCTGTTTGCTCCCCACGCTTTCGCACCTCAG | 114 | 0.11392822521811259 | No Hit |
| GACTACACGGGTATCTAATCCTGTTTGCTCCCCACGCTTTCGCGNCTCAG | 114 | 0.11392822521811259 | No Hit |
| GAGACTACTAGGGTATCTAATCCTGTTTGCTCCCCACGCTTTCGCGCCTC | 113 | 0.11292885482146248 | No Hit |
| GACTACAAGGGTATCTAATCCCGTTCGCTCCCCATGCTTTCGCACTCCAG | 113 | 0.11292885482146248 | No Hit |
| GTGACTACAGGGGTATCTAATCCTGTTTGCTCCCCACGCTTTCGCGCCTC | 113 | 0.11292885482146248 | No Hit |
| TTGACTACAAGGGTATCTAATCCTGTTTGCTCCCCACGCTTTCGCGCCTC | 111 | 0.11093011402816225 | No Hit |
| GGGACTACTAGGGTATCTAATCCTGTTTGCTCCCCACGCTTTCGCGCCTC | 110 | 0.10993074363151215 | No Hit |
| GACTACCAGGGTATCTAATCCCGTTCGCTCCCCATGCTTTCGCACTCCAG | 109 | 0.10893137323486203 | No Hit |
| ATGACTACCAGGGTATCTAATCCTGTTTGCTCCCCACGCTTTCGCGCCTC | 108 | 0.10793200283821193 | No Hit |
| GACTACAGGGGTATCTAATCCCGTTCGCTCCCCATGCTTTCGCACTCCAG | 107 | 0.1069326324415618 | No Hit |
| CTGACTACTCGGGTATCTAATCCTGTTTGCTCCCCACGCTTTCGCGCCTC | 106 | 0.10593326204491171 | No Hit |
| GGGACTACTCGGGTATCTAATCCTGTTTGCTCCCCACGCTTTCGCGCCTC | 106 | 0.10593326204491171 | No Hit |
| AAGACTACTCGGGTATCTAATCCTGTTTGCTCCCCACGCTTTCGCGCCTC | 105 | 0.10493389164826158 | No Hit |
| TCGACTACTGGGGTATCTAATCCTGTTTGCTCCCCACGCTTTCGCGCCTC | 104 | 0.10393452125161148 | No Hit |
| CGGACTACTAGGGTATCTAATCCTGTTTGCTCCCCACGCTTTCGCGCCTC | 103 | 0.10293515085496138 | No Hit |
| AGGACTACTAGGGTATCTAATCCTGTTTGCTCCCCACGCTTTCGCGCCTC | 102 | 0.10193578045831127 | No Hit |
| GGGGGGGGGGGGGGGGGGGGGGGGGGGGGGGGGGGGGGGGGGGGGGGGGG | 102 | 0.10193578045831127 | No Hit |
| TTGACTACCGGGGTATCTAATCCTGTTTGCTCCCCACGCTTTCGCGCCTC | 102 | 0.10193578045831127 | No Hit |
| TGGACTACAGGGGTATCTAATCCTGTTTGCTCCCCACGCTTTCGCGCCTC | 102 | 0.10193578045831127 | No Hit |

## Adapter Content

Produced by FastQC (version 0.12.1)
