# Supplementary material for: Bacterial community data associated with Stephanitis pyri using 16S rRNA gene metabarcoding
Source: Data Brief. 2026 Jul 29;68:113107. doi: 10.1016/j.dib.2026.113107 (PMC13474393; doi:10.1016/j.dib.2026.113107)
Supplement: Supplementary file 1 [file mmc1.zip › supplementary_material_1/fastqc/c2-N4060-16Sv3v4_S23_L001_R1_001_fastqc.zip!/c2-N4060-16Sv3v4_S23_L001_R1_001_fastqc/fastqc_report.html]

c2-N4060-16Sv3v4\_S23\_L001\_R1\_001.fastq.gz FastQC Report 

FastQC Report

Fri 7 Nov 2025  
c2-N4060-16Sv3v4\_S23\_L001\_R1\_001.fastq.gz

## Summary

- Basic Statistics
- Per base sequence quality
- Per tile sequence quality
- Per sequence quality scores
- Per base sequence content
- Per sequence GC content
- Per base N content
- Sequence Length Distribution
- Sequence Duplication Levels
- Overrepresented sequences
- Adapter Content

## Basic Statistics

| Measure | Value |
| --- | --- |
| Filename | c2-N4060-16Sv3v4\_S23\_L001\_R1\_001.fastq.gz |
| File type | Conventional base calls |
| Encoding | Sanger / Illumina 1.9 |
| Total Sequences | 100079 |
| Total Bases | 30.1 Mbp |
| Sequences flagged as poor quality | 0 |
| Sequence length | 301 |
| %GC | 55 |

## Per base sequence quality

## Per tile sequence quality

## Per sequence quality scores

## Per base sequence content

## Per sequence GC content

## Per base N content

## Sequence Length Distribution

## Sequence Duplication Levels

## Overrepresented sequences

| Sequence | Count | Percentage | Possible Source |
| --- | --- | --- | --- |
| CCTACGGGTGGCAGCAGTGGGGAATATTGGACAATGGGCGCAAGCCTGAT | 4852 | 4.848169945742863 | No Hit |
| CCTACGGGTGGCTGCAGTGGGGAATATTGGACAATGGGCGCAAGCCTGAT | 2327 | 2.325163121134304 | No Hit |
| TCCTACGGGTGGCAGCAGTGGGGAATATTGGACAATGGGCGCAAGCCTGA | 2322 | 2.320167068016267 | No Hit |
| CCTACGGGAGGCAGCAGTGGGGAATATTGGACAATGGGCGCAAGCCTGAT | 1978 | 1.9764386134953387 | No Hit |
| CCTACGGGGGGCAGCAGTGGGGAATATTGGACAATGGGCGCAAGCCTGAT | 1911 | 1.9094915017136462 | No Hit |
| ACCTACGGGTGGCAGCAGTGGGGAATATTGGACAATGGGCGCAAGCCTGA | 1545 | 1.5437804134733562 | No Hit |
| CCTACGGGCGGCAGCAGTGGGGAATATTGGACAATGGGCGCAAGCCTGAT | 1444 | 1.4428601404890136 | No Hit |
| CCTACGGGAGGCTGCAGTGGGGAATATTGGACAATGGGCGCAAGCCTGAT | 1309 | 1.3079667063020213 | No Hit |
| GCCTACGGGTGGCAGCAGTGGGGAATATTGGACAATGGGCGCAAGCCTGA | 1293 | 1.291979336324304 | No Hit |
| TCCTACGGGTGGCTGCAGTGGGGAATATTGGACAATGGGCGCAAGCCTGA | 1068 | 1.06715694601265 | No Hit |
| CCTACGGGGGGCTGCAGTGGGGAATATTGGACAATGGGCGCAAGCCTGAT | 1048 | 1.047172733540503 | No Hit |
| CCCTACGGGTGGCAGCAGTGGGGAATATTGGACAATGGGCGCAAGCCTGA | 970 | 0.9692343048991297 | No Hit |
| TCCTACGGGAGGCAGCAGTGGGGAATATTGGACAATGGGCGCAAGCCTGA | 940 | 0.9392579861909093 | No Hit |
| TCCTACGGGGGGCAGCAGTGGGGAATATTGGACAATGGGCGCAAGCCTGA | 810 | 0.8093606051219537 | No Hit |
| TTCCTACGGGTGGCAGCAGTGGGGAATATTGGACAATGGGCGCAAGCCTG | 775 | 0.7743882332956964 | No Hit |
| ACCTACGGGTGGCTGCAGTGGGGAATATTGGACAATGGGCGCAAGCCTGA | 724 | 0.7234284914917215 | No Hit |
| GCCTACGGGAGGCAGCAGTGGGGAATATTGGACAATGGGCGCAAGCCTGA | 643 | 0.6424924309795261 | No Hit |
| ACCTACGGGAGGCAGCAGTGGGGAATATTGGACAATGGGCGCAAGCCTGA | 638 | 0.6374963778614894 | No Hit |
| CCTACGGGCGGCTGCAGTGGGGAATATTGGACAATGGGCGCAAGCCTGAT | 621 | 0.6205097972601645 | No Hit |
| TCCTACGGGAGGCTGCAGTGGGGAATATTGGACAATGGGCGCAAGCCTGA | 619 | 0.6185113760129497 | No Hit |
| ACCTACGGGGGGCAGCAGTGGGGAATATTGGACAATGGGCGCAAGCCTGA | 604 | 0.6035232166588395 | No Hit |
| GCCTACGGGTGGCTGCAGTGGGGAATATTGGACAATGGGCGCAAGCCTGA | 580 | 0.5795421616922631 | No Hit |
| TCCTACGGGCGGCAGCAGTGGGGAATATTGGACAATGGGCGCAAGCCTGA | 548 | 0.5475674217368279 | No Hit |
| TACCTACGGGTGGCAGCAGTGGGGAATATTGGACAATGGGCGCAAGCCTG | 540 | 0.5395737367479692 | No Hit |
| ATCCTACGGGTGGCAGCAGTGGGGAATATTGGACAATGGGCGCAAGCCTG | 482 | 0.48161952057874274 | No Hit |
| GCCTACGGGGGGCAGCAGTGGGGAATATTGGACAATGGGCGCAAGCCTGA | 460 | 0.45963688685938103 | No Hit |
| TCCTACGGGGGGCTGCAGTGGGGAATATTGGACAATGGGCGCAAGCCTGA | 453 | 0.4526424124941296 | No Hit |
| ACCTACGGGAGGCTGCAGTGGGGAATATTGGACAATGGGCGCAAGCCTGA | 446 | 0.4456479381288782 | No Hit |
| GTCCTACGGGTGGCAGCAGTGGGGAATATTGGACAATGGGCGCAAGCCTG | 446 | 0.4456479381288782 | No Hit |
| CCCTACGGGTGGCTGCAGTGGGGAATATTGGACAATGGGCGCAAGCCTGA | 432 | 0.43165898939837527 | No Hit |
| GCCTACGGGAGGCTGCAGTGGGGAATATTGGACAATGGGCGCAAGCCTGA | 410 | 0.40967635567901356 | No Hit |
| CCCTACGGGAGGCAGCAGTGGGGAATATTGGACAATGGGCGCAAGCCTGA | 408 | 0.4076779344317989 | No Hit |
| ACCTACGGGCGGCAGCAGTGGGGAATATTGGACAATGGGCGCAAGCCTGA | 397 | 0.396686617572118 | No Hit |
| CTCCTACGGGTGGCAGCAGTGGGGAATATTGGACAATGGGCGCAAGCCTG | 387 | 0.38669451133604454 | No Hit |
| TTCCTACGGGTGGCTGCAGTGGGGAATATTGGACAATGGGCGCAAGCCTG | 387 | 0.38669451133604454 | No Hit |
| AACCTACGGGTGGCAGCAGTGGGGAATATTGGACAATGGGCGCAAGCCTG | 383 | 0.3826976688416151 | No Hit |
| TGCCTACGGGTGGCAGCAGTGGGGAATATTGGACAATGGGCGCAAGCCTG | 372 | 0.37170635198193425 | No Hit |
| TCCCTACGGGTGGCAGCAGTGGGGAATATTGGACAATGGGCGCAAGCCTG | 358 | 0.35771740325143137 | No Hit |
| TTCCTACGGGAGGCAGCAGTGGGGAATATTGGACAATGGGCGCAAGCCTG | 337 | 0.336733980155677 | No Hit |
| ACCTACGGGGGGCTGCAGTGGGGAATATTGGACAATGGGCGCAAGCCTGA | 336 | 0.33573476953206965 | No Hit |
| CACCTACGGGTGGCAGCAGTGGGGAATATTGGACAATGGGCGCAAGCCTG | 332 | 0.33173792703764027 | No Hit |
| GACCTACGGGTGGCAGCAGTGGGGAATATTGGACAATGGGCGCAAGCCTG | 315 | 0.3147513464363153 | No Hit |
| AGCCTACGGGTGGCAGCAGTGGGGAATATTGGACAATGGGCGCAAGCCTG | 309 | 0.3087560826946712 | No Hit |
| TTCCTACGGGGGGCAGCAGTGGGGAATATTGGACAATGGGCGCAAGCCTG | 293 | 0.2927687127169536 | No Hit |
| GCCTACGGGCGGCAGCAGTGGGGAATATTGGACAATGGGCGCAAGCCTGA | 292 | 0.29176950209334623 | No Hit |
| CCCTACGGGGGGCAGCAGTGGGGAATATTGGACAATGGGCGCAAGCCTGA | 274 | 0.27378371086841397 | No Hit |
| GGCCTACGGGTGGCAGCAGTGGGGAATATTGGACAATGGGCGCAAGCCTG | 269 | 0.26878765775037716 | No Hit |
| CCTACGGGTGGCAGCAGTGGGGAATATTGGACAATGGGCGAAAGCCTGAT | 262 | 0.26179318338512575 | No Hit |
| CCTACGGGTGGCTGCATTTGATACGTCTAGACTAGAGTGTGAGAGAGGGT | 254 | 0.253799498396267 | No Hit |
| CCCTACGGGAGGCTGCAGTGGGGAATATTGGACAATGGGCGCAAGCCTGA | 250 | 0.24980265590183753 | No Hit |
| ACCCTACGGGTGGCAGCAGTGGGGAATATTGGACAATGGGCGCAAGCCTG | 247 | 0.24680502403101548 | No Hit |
| CCTACGGGTGGCAGCAGTGGGGAATCTTGGACAATGGGCGAAAGCCCGAT | 246 | 0.24580581340740817 | No Hit |
| GCCTACGGGGGGCTGCAGTGGGGAATATTGGACAATGGGCGCAAGCCTGA | 244 | 0.24380739216019343 | No Hit |
| GCCCTACGGGTGGCAGCAGTGGGGAATATTGGACAATGGGCGCAAGCCTG | 243 | 0.24280818153658612 | No Hit |
| TTTCCTACGGGTGGCAGCAGTGGGGAATATTGGACAATGGGCGCAAGCCT | 238 | 0.23781212841854932 | No Hit |
| ATCCTACGGGTGGCTGCAGTGGGGAATATTGGACAATGGGCGCAAGCCTG | 238 | 0.23781212841854932 | No Hit |
| CCCTACGGGCGGCAGCAGTGGGGAATATTGGACAATGGGCGCAAGCCTGA | 231 | 0.2308176540532979 | No Hit |
| TACCTACGGGAGGCAGCAGTGGGGAATATTGGACAATGGGCGCAAGCCTG | 226 | 0.22582160093526116 | No Hit |
| TTCCTACGGGAGGCTGCAGTGGGGAATATTGGACAATGGGCGCAAGCCTG | 224 | 0.22382317968804646 | No Hit |
| TACCTACGGGTGGCTGCAGTGGGGAATATTGGACAATGGGCGCAAGCCTG | 222 | 0.22182475844083174 | No Hit |
| TACCTACGGGGGGCAGCAGTGGGGAATATTGGACAATGGGCGCAAGCCTG | 217 | 0.216828705322795 | No Hit |
| TCCTACGGGCGGCTGCAGTGGGGAATATTGGACAATGGGCGCAAGCCTGA | 217 | 0.216828705322795 | No Hit |
| ATCCTACGGGAGGCAGCAGTGGGGAATATTGGACAATGGGCGCAAGCCTG | 212 | 0.21183265220475825 | No Hit |
| GTCCTACGGGAGGCAGCAGTGGGGAATATTGGACAATGGGCGCAAGCCTG | 201 | 0.2008413353450774 | No Hit |
| GTCCTACGGGTGGCTGCAGTGGGGAATATTGGACAATGGGCGCAAGCCTG | 200 | 0.19984212472147003 | No Hit |
| TTCCTACGGGCGGCAGCAGTGGGGAATATTGGACAATGGGCGCAAGCCTG | 194 | 0.19384686097982595 | No Hit |
| TATCCTACGGGTGGCAGCAGTGGGGAATATTGGACAATGGGCGCAAGCCT | 192 | 0.19184843973261123 | No Hit |
| TGCCTACGGGAGGCAGCAGTGGGGAATATTGGACAATGGGCGCAAGCCTG | 188 | 0.18785159723818184 | No Hit |
| ATCCTACGGGGGGCAGCAGTGGGGAATATTGGACAATGGGCGCAAGCCTG | 180 | 0.17985791224932304 | No Hit |
| AACCTACGGGTGGCTGCAGTGGGGAATATTGGACAATGGGCGCAAGCCTG | 180 | 0.17985791224932304 | No Hit |
| TTACCTACGGGTGGCAGCAGTGGGGAATATTGGACAATGGGCGCAAGCCT | 179 | 0.17885870162571568 | No Hit |
| ATTCCTACGGGTGGCAGCAGTGGGGAATATTGGACAATGGGCGCAAGCCT | 179 | 0.17885870162571568 | No Hit |
| TACCTACGGGAGGCTGCAGTGGGGAATATTGGACAATGGGCGCAAGCCTG | 174 | 0.17386264850767894 | No Hit |
| TGCCTACGGGTGGCTGCAGTGGGGAATATTGGACAATGGGCGCAAGCCTG | 173 | 0.1728634378840716 | No Hit |
| GTCCTACGGGGGGCAGCAGTGGGGAATATTGGACAATGGGCGCAAGCCTG | 171 | 0.17086501663685688 | No Hit |
| AACCTACGGGGGGCAGCAGTGGGGAATATTGGACAATGGGCGCAAGCCTG | 167 | 0.1668681741424275 | No Hit |
| CGCCTACGGGTGGCAGCAGTGGGGAATATTGGACAATGGGCGCAAGCCTG | 166 | 0.16586896351882013 | No Hit |
| TCCCTACGGGTGGCTGCAGTGGGGAATATTGGACAATGGGCGCAAGCCTG | 165 | 0.16486975289521277 | No Hit |
| GACCTACGGGAGGCAGCAGTGGGGAATATTGGACAATGGGCGCAAGCCTG | 164 | 0.16387054227160544 | No Hit |
| AACCTACGGGAGGCAGCAGTGGGGAATATTGGACAATGGGCGCAAGCCTG | 164 | 0.16387054227160544 | No Hit |
| CTCCTACGGGAGGCAGCAGTGGGGAATATTGGACAATGGGCGCAAGCCTG | 163 | 0.16287133164799808 | No Hit |
| AGCCTACGGGTGGCTGCAGTGGGGAATATTGGACAATGGGCGCAAGCCTG | 163 | 0.16287133164799808 | No Hit |
| CTCCTACGGGTGGCTGCAGTGGGGAATATTGGACAATGGGCGCAAGCCTG | 163 | 0.16287133164799808 | No Hit |
| GACCTACGGGTGGCTGCAGTGGGGAATATTGGACAATGGGCGCAAGCCTG | 159 | 0.1588744891535687 | No Hit |
| TACCTACGGGCGGCAGCAGTGGGGAATATTGGACAATGGGCGCAAGCCTG | 155 | 0.15487764665913928 | No Hit |
| CCCTACGGGGGGCTGCAGTGGGGAATATTGGACAATGGGCGCAAGCCTGA | 152 | 0.15188001478831722 | No Hit |
| GGCCTACGGGAGGCAGCAGTGGGGAATATTGGACAATGGGCGCAAGCCTG | 151 | 0.15088080416470986 | No Hit |
| CACCTACGGGTGGCTGCAGTGGGGAATATTGGACAATGGGCGCAAGCCTG | 151 | 0.15088080416470986 | No Hit |
| AATCCTACGGGTGGCAGCAGTGGGGAATATTGGACAATGGGCGCAAGCCT | 147 | 0.14688396167028048 | No Hit |
| ACCTACGGGCGGCTGCAGTGGGGAATATTGGACAATGGGCGCAAGCCTGA | 145 | 0.14488554042306578 | No Hit |
| ATCCTACGGGAGGCTGCAGTGGGGAATATTGGACAATGGGCGCAAGCCTG | 144 | 0.14388632979945845 | No Hit |
| TTCCTACGGGGGGCTGCAGTGGGGAATATTGGACAATGGGCGCAAGCCTG | 143 | 0.14288711917585106 | No Hit |
| CCCCTACGGGTGGCAGCAGTGGGGAATATTGGACAATGGGCGCAAGCCTG | 142 | 0.14188790855224373 | No Hit |
| TGCCTACGGGGGGCAGCAGTGGGGAATATTGGACAATGGGCGCAAGCCTG | 141 | 0.1408886979286364 | No Hit |
| AGCCTACGGGGGGCAGCAGTGGGGAATATTGGACAATGGGCGCAAGCCTG | 141 | 0.1408886979286364 | No Hit |
| CCTACGGGGGGCAGCAGTGGGGAATCTTGGACAATGGGCGAAAGCCCGAT | 140 | 0.139889487305029 | No Hit |
| CCTACGGGTGGCTGCAGTGGGGAATCTTGGACAATGGGCGAAAGCCCGAT | 135 | 0.1348934341869923 | No Hit |
| AACCTACGGGCGGCAGCAGTGGGGAATATTGGACAATGGGCGCAAGCCTG | 135 | 0.1348934341869923 | No Hit |
| CCTACGGGGGGCTGCATTTGATACGTCTAGACTAGAGTGTGAGAGAGGGT | 131 | 0.13089659169256287 | No Hit |
| AGCCTACGGGAGGCAGCAGTGGGGAATATTGGACAATGGGCGCAAGCCTG | 129 | 0.12889817044534818 | No Hit |
| GTCCTACGGGAGGCTGCAGTGGGGAATATTGGACAATGGGCGCAAGCCTG | 129 | 0.12889817044534818 | No Hit |
| GGCCTACGGGTGGCTGCAGTGGGGAATATTGGACAATGGGCGCAAGCCTG | 128 | 0.12789895982174082 | No Hit |
| TGTCCTACGGGTGGCAGCAGTGGGGAATATTGGACAATGGGCGCAAGCCT | 125 | 0.12490132795091877 | No Hit |
| TCCCTACGGGAGGCAGCAGTGGGGAATATTGGACAATGGGCGCAAGCCTG | 125 | 0.12490132795091877 | No Hit |
| TCCCTACGGGGGGCAGCAGTGGGGAATATTGGACAATGGGCGCAAGCCTG | 122 | 0.12190369608009671 | No Hit |
| TTCCCTACGGGTGGCAGCAGTGGGGAATATTGGACAATGGGCGCAAGCCT | 119 | 0.11890606420927466 | No Hit |
| GACCTACGGGGGGCAGCAGTGGGGAATATTGGACAATGGGCGCAAGCCTG | 118 | 0.11790685358566731 | No Hit |
| TCCTACGGGTGGCAGCAGTGGGGAATCTTGGACAATGGGCGAAAGCCCGA | 117 | 0.11690764296205998 | No Hit |
| GTTCCTACGGGTGGCAGCAGTGGGGAATATTGGACAATGGGCGCAAGCCT | 117 | 0.11690764296205998 | No Hit |
| ACCCTACGGGTGGCTGCAGTGGGGAATATTGGACAATGGGCGCAAGCCTG | 116 | 0.11590843233845263 | No Hit |
| ACCCTACGGGAGGCAGCAGTGGGGAATATTGGACAATGGGCGCAAGCCTG | 115 | 0.11490922171484526 | No Hit |
| TCCTACGGGTGGCAGCAGTGGGGAATATTGGACAATGGGCGAAAGCCTGA | 115 | 0.11490922171484526 | No Hit |
| GCCTACGGGCGGCTGCAGTGGGGAATATTGGACAATGGGCGCAAGCCTGA | 115 | 0.11490922171484526 | No Hit |
| TAACCTACGGGTGGCAGCAGTGGGGAATATTGGACAATGGGCGCAAGCCT | 113 | 0.11291080046763058 | No Hit |
| TTTCCTACGGGTGGCTGCAGTGGGGAATATTGGACAATGGGCGCAAGCCT | 113 | 0.11291080046763058 | No Hit |
| GCCCTACGGGTGGCTGCAGTGGGGAATATTGGACAATGGGCGCAAGCCTG | 111 | 0.11091237922041587 | No Hit |
| ATCCTACGGGCGGCAGCAGTGGGGAATATTGGACAATGGGCGCAAGCCTG | 111 | 0.11091237922041587 | No Hit |
| TAGCCTACGGGTGGCAGCAGTGGGGAATATTGGACAATGGGCGCAAGCCT | 110 | 0.10991316859680852 | No Hit |
| TGCCTACGGGAGGCTGCAGTGGGGAATATTGGACAATGGGCGCAAGCCTG | 110 | 0.10991316859680852 | No Hit |
| AACCTACGGGAGGCTGCAGTGGGGAATATTGGACAATGGGCGCAAGCCTG | 109 | 0.10891395797320118 | No Hit |
| TCCTACGGGTGGCTGCATTTGATACGTCTAGACTAGAGTGTGAGAGAGGG | 109 | 0.10891395797320118 | No Hit |
| CCTACGGGAGGCAGCAGTGGGGAATATTGGACAATGGGCGAAAGCCTGAT | 108 | 0.10791474734959382 | No Hit |
| TTGCCTACGGGTGGCAGCAGTGGGGAATATTGGACAATGGGCGCAAGCCT | 106 | 0.10591632610237912 | No Hit |
| TACCTACGGGGGGCTGCAGTGGGGAATATTGGACAATGGGCGCAAGCCTG | 106 | 0.10591632610237912 | No Hit |
| ATACCTACGGGTGGCAGCAGTGGGGAATATTGGACAATGGGCGCAAGCCT | 105 | 0.10491711547877178 | No Hit |
| GGCCTACGGGGGGCAGCAGTGGGGAATATTGGACAATGGGCGCAAGCCTG | 104 | 0.10391790485516442 | No Hit |
| CCTACGGGAGGCAGCAGTGGGGAATCTTGGACAATGGGCGAAAGCCCGAT | 103 | 0.10291869423155707 | No Hit |
| ATCCCTACGGGTGGCAGCAGTGGGGAATATTGGACAATGGGCGCAAGCCT | 101 | 0.10092027298434236 | No Hit |
| TTTCCTACGGGGGGCAGCAGTGGGGAATATTGGACAATGGGCGCAAGCCT | 101 | 0.10092027298434236 | No Hit |
| CCTACGGGAGGCTGCATTTGATACGTCTAGACTAGAGTGTGAGAGAGGGT | 101 | 0.10092027298434236 | No Hit |

## Adapter Content

Produced by FastQC (version 0.12.1)
