# Supplementary material for: Bacterial community data associated with Stephanitis pyri using 16S rRNA gene metabarcoding
Source: Data Brief. 2026 Jul 29;68:113107. doi: 10.1016/j.dib.2026.113107 (PMC13474393; doi:10.1016/j.dib.2026.113107)
Supplement: Supplementary file 1 [file mmc1.zip › supplementary_material_1/fastqc/c2-N4060-16Sv3v4_S23_L001_R2_001_fastqc.zip!/c2-N4060-16Sv3v4_S23_L001_R2_001_fastqc/fastqc_report.html]

c2-N4060-16Sv3v4\_S23\_L001\_R2\_001.fastq.gz FastQC Report 

FastQC Report

Fri 7 Nov 2025  
c2-N4060-16Sv3v4\_S23\_L001\_R2\_001.fastq.gz

## Summary

- Basic Statistics
- Per base sequence quality
- Per tile sequence quality
- Per sequence quality scores
- Per base sequence content
- Per sequence GC content
- Per base N content
- Sequence Length Distribution
- Sequence Duplication Levels
- Overrepresented sequences
- Adapter Content

## Basic Statistics

| Measure | Value |
| --- | --- |
| Filename | c2-N4060-16Sv3v4\_S23\_L001\_R2\_001.fastq.gz |
| File type | Conventional base calls |
| Encoding | Sanger / Illumina 1.9 |
| Total Sequences | 100079 |
| Total Bases | 30.1 Mbp |
| Sequences flagged as poor quality | 0 |
| Sequence length | 301 |
| %GC | 54 |

## Per base sequence quality

## Per tile sequence quality

## Per sequence quality scores

## Per base sequence content

## Per sequence GC content

## Per base N content

## Sequence Length Distribution

## Sequence Duplication Levels

## Overrepresented sequences

| Sequence | Count | Percentage | Possible Source |
| --- | --- | --- | --- |
| GACTACTAGGGTATCTAATCCTGTTTGCTCCCCACGCTTTCGCGCCTCAG | 2700 | 2.6978686837398453 | No Hit |
| GACTACTCGGGTATCTAATCCTGTTTGCTCCCCACGCTTTCGCGCCTCAG | 2448 | 2.4460676065907934 | No Hit |
| GACTACTGGGGTATCTAATCCTGTTTGCTCCCCACGCTTTCGCGCCTCAG | 2121 | 2.11932573267119 | No Hit |
| GACTACAAGGGTATCTAATCCTGTTTGCTCCCCACGCTTTCGCGCCTCAG | 2028 | 2.026399144675706 | No Hit |
| GACTACCAGGGTATCTAATCCTGTTTGCTCCCCACGCTTTCGCGCCTCAG | 1844 | 1.8425443899319538 | No Hit |
| GACTACACGGGTATCTAATCCTGTTTGCTCCCCACGCTTTCGCGCCTCAG | 1657 | 1.6556920033173792 | No Hit |
| GACTACAGGGGTATCTAATCCTGTTTGCTCCCCACGCTTTCGCGCCTCAG | 1550 | 1.5487764665913928 | No Hit |
| GACTACCGGGGTATCTAATCCTGTTTGCTCCCCACGCTTTCGCGCCTCAG | 1405 | 1.403890926168327 | No Hit |
| GACTACCCGGGTATCTAATCCTGTTTGCTCCCCACGCTTTCGCGCCTCAG | 1156 | 1.1550874808900968 | No Hit |
| TGACTACTAGGGTATCTAATCCTGTTTGCTCCCCACGCTTTCGCGCCTCA | 953 | 0.9522477242978047 | No Hit |
| TGACTACTCGGGTATCTAATCCTGTTTGCTCCCCACGCTTTCGCGCCTCA | 792 | 0.7913748138970214 | No Hit |
| TGACTACAAGGGTATCTAATCCTGTTTGCTCCCCACGCTTTCGCGCCTCA | 748 | 0.7474095464582979 | No Hit |
| TGACTACTGGGGTATCTAATCCTGTTTGCTCCCCACGCTTTCGCGCCTCA | 626 | 0.6255058503782013 | No Hit |
| AGACTACTAGGGTATCTAATCCTGTTTGCTCCCCACGCTTTCGCGCCTCA | 609 | 0.6085192697768762 | No Hit |
| TGACTACCAGGGTATCTAATCCTGTTTGCTCCCCACGCTTTCGCGCCTCA | 569 | 0.5685508448325822 | No Hit |
| AGACTACTCGGGTATCTAATCCTGTTTGCTCCCCACGCTTTCGCGCCTCA | 531 | 0.530580841135503 | No Hit |
| CGACTACTAGGGTATCTAATCCTGTTTGCTCCCCACGCTTTCGCGCCTCA | 529 | 0.5285824198882882 | No Hit |
| GGACTACTAGGGTATCTAATCCTGTTTGCTCCCCACGCTTTCGCGCCTCA | 523 | 0.5225871561466442 | No Hit |
| AGACTACTGGGGTATCTAATCCTGTTTGCTCCCCACGCTTTCGCGCCTCA | 513 | 0.5125950499105706 | No Hit |
| TGACTACAGGGGTATCTAATCCTGTTTGCTCCCCACGCTTTCGCGCCTCA | 504 | 0.5036021542981045 | No Hit |
| GGACTACTGGGGTATCTAATCCTGTTTGCTCCCCACGCTTTCGCGCCTCA | 492 | 0.49161162681481635 | No Hit |
| GGACTACTCGGGTATCTAATCCTGTTTGCTCCCCACGCTTTCGCGCCTCA | 473 | 0.47262662496627666 | No Hit |
| TGACTACACGGGTATCTAATCCTGTTTGCTCCCCACGCTTTCGCGCCTCA | 462 | 0.4616353081065958 | No Hit |
| TGACTACCGGGGTATCTAATCCTGTTTGCTCCCCACGCTTTCGCGCCTCA | 455 | 0.45464083374134434 | No Hit |
| GGACTACAAGGGTATCTAATCCTGTTTGCTCCCCACGCTTTCGCGCCTCA | 448 | 0.44764635937609293 | No Hit |
| TGACTACCCGGGTATCTAATCCTGTTTGCTCCCCACGCTTTCGCGCCTCA | 429 | 0.4286613575275532 | No Hit |
| CGACTACTCGGGTATCTAATCCTGTTTGCTCCCCACGCTTTCGCGCCTCA | 425 | 0.4246645150331238 | No Hit |
| CGACTACAAGGGTATCTAATCCTGTTTGCTCCCCACGCTTTCGCGCCTCA | 423 | 0.4226660937859092 | No Hit |
| CGACTACTGGGGTATCTAATCCTGTTTGCTCCCCACGCTTTCGCGCCTCA | 408 | 0.4076779344317989 | No Hit |
| AGACTACCAGGGTATCTAATCCTGTTTGCTCCCCACGCTTTCGCGCCTCA | 393 | 0.39268977507768865 | No Hit |
| AGACTACAAGGGTATCTAATCCTGTTTGCTCCCCACGCTTTCGCGCCTCA | 388 | 0.3876937219596519 | No Hit |
| CGACTACCAGGGTATCTAATCCTGTTTGCTCCCCACGCTTTCGCGCCTCA | 373 | 0.3727055626055416 | No Hit |
| GGACTACCAGGGTATCTAATCCTGTTTGCTCCCCACGCTTTCGCGCCTCA | 365 | 0.36471187761668283 | No Hit |
| AGACTACCGGGGTATCTAATCCTGTTTGCTCCCCACGCTTTCGCGCCTCA | 355 | 0.3547197713806093 | No Hit |
| AGACTACACGGGTATCTAATCCTGTTTGCTCCCCACGCTTTCGCGCCTCA | 346 | 0.3457268757681432 | No Hit |
| GGACTACAGGGGTATCTAATCCTGTTTGCTCCCCACGCTTTCGCGCCTCA | 342 | 0.34173003327371376 | No Hit |
| AGACTACAGGGGTATCTAATCCTGTTTGCTCCCCACGCTTTCGCGCCTCA | 341 | 0.3407308226501064 | No Hit |
| GGACTACACGGGTATCTAATCCTGTTTGCTCCCCACGCTTTCGCGCCTCA | 327 | 0.3267418739196035 | No Hit |
| CGACTACACGGGTATCTAATCCTGTTTGCTCCCCACGCTTTCGCGCCTCA | 306 | 0.30575845082384917 | No Hit |
| AGACTACCCGGGTATCTAATCCTGTTTGCTCCCCACGCTTTCGCGCCTCA | 300 | 0.29976318708220506 | No Hit |
| TTGACTACTAGGGTATCTAATCCTGTTTGCTCCCCACGCTTTCGCGCCTC | 280 | 0.279778974610058 | No Hit |
| CGACTACAGGGGTATCTAATCCTGTTTGCTCCCCACGCTTTCGCGCCTCA | 279 | 0.2787797639864507 | No Hit |
| TTGACTACTCGGGTATCTAATCCTGTTTGCTCCCCACGCTTTCGCGCCTC | 278 | 0.27778055336284335 | No Hit |
| GGACTACCGGGGTATCTAATCCTGTTTGCTCCCCACGCTTTCGCGCCTCA | 265 | 0.26479081525594783 | No Hit |
| CGACTACCCGGGTATCTAATCCTGTTTGCTCCCCACGCTTTCGCGCCTCA | 253 | 0.2528002877726596 | No Hit |
| GGACTACCCGGGTATCTAATCCTGTTTGCTCCCCACGCTTTCGCGCCTCA | 231 | 0.2308176540532979 | No Hit |
| TAGACTACTAGGGTATCTAATCCTGTTTGCTCCCCACGCTTTCGCGCCTC | 205 | 0.20483817783950678 | No Hit |
| TTGACTACTGGGGTATCTAATCCTGTTTGCTCCCCACGCTTTCGCGCCTC | 197 | 0.19684449285064798 | No Hit |
| ATGACTACTAGGGTATCTAATCCTGTTTGCTCCCCACGCTTTCGCGCCTC | 190 | 0.18985001848539654 | No Hit |
| GTGACTACTCGGGTATCTAATCCTGTTTGCTCCCCACGCTTTCGCGCCTC | 188 | 0.18785159723818184 | No Hit |
| TTGACTACCAGGGTATCTAATCCTGTTTGCTCCCCACGCTTTCGCGCCTC | 185 | 0.1848539653673598 | No Hit |
| GTGACTACTAGGGTATCTAATCCTGTTTGCTCCCCACGCTTTCGCGCCTC | 181 | 0.18085712287293038 | No Hit |
| CGACTACCGGGGTATCTAATCCTGTTTGCTCCCCACGCTTTCGCGCCTCA | 179 | 0.17885870162571568 | No Hit |
| TAGACTACTCGGGTATCTAATCCTGTTTGCTCCCCACGCTTTCGCGCCTC | 177 | 0.176860280378501 | No Hit |
| GACTACTAGGGTATCTAATCCTGTTTGCTCCCCACGCTTTCGCGNCTCAG | 170 | 0.16986580601324955 | No Hit |
| TTGACTACAGGGGTATCTAATCCTGTTTGCTCCCCACGCTTTCGCGCCTC | 166 | 0.16586896351882013 | No Hit |
| TAGACTACTGGGGTATCTAATCCTGTTTGCTCCCCACGCTTTCGCGCCTC | 165 | 0.16486975289521277 | No Hit |
| TGGACTACTAGGGTATCTAATCCTGTTTGCTCCCCACGCTTTCGCGCCTC | 164 | 0.16387054227160544 | No Hit |
| TTGACTACAAGGGTATCTAATCCTGTTTGCTCCCCACGCTTTCGCGCCTC | 159 | 0.1588744891535687 | No Hit |
| ATGACTACAAGGGTATCTAATCCTGTTTGCTCCCCACGCTTTCGCGCCTC | 158 | 0.15787527852996133 | No Hit |
| TAGACTACAAGGGTATCTAATCCTGTTTGCTCCCCACGCTTTCGCGCCTC | 158 | 0.15787527852996133 | No Hit |
| GTGACTACTGGGGTATCTAATCCTGTTTGCTCCCCACGCTTTCGCGCCTC | 156 | 0.15587685728274664 | No Hit |
| TGGACTACTCGGGTATCTAATCCTGTTTGCTCCCCACGCTTTCGCGCCTC | 155 | 0.15487764665913928 | No Hit |
| GACTACTAGGGTATCTAATCCCGTTCGCTCCCCATGCTTTCGCACTCCAG | 155 | 0.15487764665913928 | No Hit |
| TTGACTACACGGGTATCTAATCCTGTTTGCTCCCCACGCTTTCGCGCCTC | 151 | 0.15088080416470986 | No Hit |
| ATGACTACTGGGGTATCTAATCCTGTTTGCTCCCCACGCTTTCGCGCCTC | 150 | 0.14988159354110253 | No Hit |
| TCGACTACTGGGGTATCTAATCCTGTTTGCTCCCCACGCTTTCGCGCCTC | 149 | 0.14888238291749517 | No Hit |
| TTGACTACCGGGGTATCTAATCCTGTTTGCTCCCCACGCTTTCGCGCCTC | 147 | 0.14688396167028048 | No Hit |
| TCGACTACTAGGGTATCTAATCCTGTTTGCTCCCCACGCTTTCGCGCCTC | 147 | 0.14688396167028048 | No Hit |
| ATGACTACTCGGGTATCTAATCCTGTTTGCTCCCCACGCTTTCGCGCCTC | 147 | 0.14688396167028048 | No Hit |
| GTGACTACAAGGGTATCTAATCCTGTTTGCTCCCCACGCTTTCGCGCCTC | 146 | 0.14588475104667312 | No Hit |
| CTGACTACTCGGGTATCTAATCCTGTTTGCTCCCCACGCTTTCGCGCCTC | 144 | 0.14388632979945845 | No Hit |
| TGGACTACAAGGGTATCTAATCCTGTTTGCTCCCCACGCTTTCGCGCCTC | 140 | 0.139889487305029 | No Hit |
| GACTACTGGGGTATCTAATCCCGTTCGCTCCCCATGCTTTCGCACTCCAG | 140 | 0.139889487305029 | No Hit |
| AAGACTACTAGGGTATCTAATCCTGTTTGCTCCCCACGCTTTCGCGCCTC | 139 | 0.13889027668142168 | No Hit |
| CTGACTACTAGGGTATCTAATCCTGTTTGCTCCCCACGCTTTCGCGCCTC | 139 | 0.13889027668142168 | No Hit |
| CTGACTACTGGGGTATCTAATCCTGTTTGCTCCCCACGCTTTCGCGCCTC | 137 | 0.13689185543420698 | No Hit |
| GACTACTCGGGTATCTAATCCTGTTTGCTCCCCACGCTTTCGCGNCTCAG | 137 | 0.13689185543420698 | No Hit |
| GACTACAAGGGTATCTAATCCTGTTTGCTCCCCACGCTTTCGCGNCTCAG | 136 | 0.13589264481059962 | No Hit |
| TCGACTACAAGGGTATCTAATCCTGTTTGCTCCCCACGCTTTCGCGCCTC | 134 | 0.13389422356338493 | No Hit |
| CAGACTACTAGGGTATCTAATCCTGTTTGCTCCCCACGCTTTCGCGCCTC | 134 | 0.13389422356338493 | No Hit |
| TCGACTACTCGGGTATCTAATCCTGTTTGCTCCCCACGCTTTCGCGCCTC | 131 | 0.13089659169256287 | No Hit |
| GACTACTAGGGTATCTAATCCTGTTTGCTCCCCACGCTTTCGCACCTCAG | 129 | 0.12889817044534818 | No Hit |
| AAGACTACTCGGGTATCTAATCCTGTTTGCTCCCCACGCTTTCGCGCCTC | 128 | 0.12789895982174082 | No Hit |
| TAGACTACCAGGGTATCTAATCCTGTTTGCTCCCCACGCTTTCGCGCCTC | 128 | 0.12789895982174082 | No Hit |
| GGGACTACTAGGGTATCTAATCCTGTTTGCTCCCCACGCTTTCGCGCCTC | 128 | 0.12789895982174082 | No Hit |
| AGGACTACTAGGGTATCTAATCCTGTTTGCTCCCCACGCTTTCGCGCCTC | 127 | 0.1268997491981335 | No Hit |
| AAGACTACTGGGGTATCTAATCCTGTTTGCTCCCCACGCTTTCGCGCCTC | 127 | 0.1268997491981335 | No Hit |
| ACGACTACTAGGGTATCTAATCCTGTTTGCTCCCCACGCTTTCGCGCCTC | 126 | 0.12590053857452613 | No Hit |
| TGGACTACTGGGGTATCTAATCCTGTTTGCTCCCCACGCTTTCGCGCCTC | 126 | 0.12590053857452613 | No Hit |
| CTGACTACAAGGGTATCTAATCCTGTTTGCTCCCCACGCTTTCGCGCCTC | 123 | 0.12290290670370409 | No Hit |
| ATGACTACCAGGGTATCTAATCCTGTTTGCTCCCCACGCTTTCGCGCCTC | 123 | 0.12290290670370409 | No Hit |
| GACTACTCGGGTATCTAATCCCGTTCGCTCCCCATGCTTTCGCACTCCAG | 123 | 0.12290290670370409 | No Hit |
| ATGACTACAGGGGTATCTAATCCTGTTTGCTCCCCACGCTTTCGCGCCTC | 123 | 0.12290290670370409 | No Hit |
| TAGACTACAGGGGTATCTAATCCTGTTTGCTCCCCACGCTTTCGCGCCTC | 120 | 0.11990527483288203 | No Hit |
| GACTACTGGGGTATCTAATCCTGTTTGCTCCCCACGCTTTCGCGNCTCAG | 120 | 0.11990527483288203 | No Hit |
| CGGACTACTAGGGTATCTAATCCTGTTTGCTCCCCACGCTTTCGCGCCTC | 116 | 0.11590843233845263 | No Hit |
| GACTACTCGGGTATCTAATCCTGTTTGCTCCCCACGCTTTCGCACCTCAG | 116 | 0.11590843233845263 | No Hit |
| TTGACTACCCGGGTATCTAATCCTGTTTGCTCCCCACGCTTTCGCGCCTC | 115 | 0.11490922171484526 | No Hit |
| GTGACTACCAGGGTATCTAATCCTGTTTGCTCCCCACGCTTTCGCGCCTC | 115 | 0.11490922171484526 | No Hit |
| CAGACTACTCGGGTATCTAATCCTGTTTGCTCCCCACGCTTTCGCGCCTC | 114 | 0.11391001109123793 | No Hit |
| GAGACTACTAGGGTATCTAATCCTGTTTGCTCCCCACGCTTTCGCGCCTC | 113 | 0.11291080046763058 | No Hit |
| ATGACTACACGGGTATCTAATCCTGTTTGCTCCCCACGCTTTCGCGCCTC | 112 | 0.11191158984402323 | No Hit |
| GCGACTACTAGGGTATCTAATCCTGTTTGCTCCCCACGCTTTCGCGCCTC | 112 | 0.11191158984402323 | No Hit |
| ACGACTACTGGGGTATCTAATCCTGTTTGCTCCCCACGCTTTCGCGCCTC | 111 | 0.11091237922041587 | No Hit |
| GACTACCAGGGTATCTAATCCTGTTTGCTCCCCACGCTTTCGCGNCTCAG | 109 | 0.10891395797320118 | No Hit |
| TGGACTACAGGGGTATCTAATCCTGTTTGCTCCCCACGCTTTCGCGCCTC | 109 | 0.10891395797320118 | No Hit |
| GACTACTGGGGTATCTAATCCTGTTTGCTCCCCACGCTTTCGCACCTCAG | 108 | 0.10791474734959382 | No Hit |
| TAGACTACACGGGTATCTAATCCTGTTTGCTCCCCACGCTTTCGCGCCTC | 106 | 0.10591632610237912 | No Hit |
| AAGACTACCAGGGTATCTAATCCTGTTTGCTCCCCACGCTTTCGCGCCTC | 105 | 0.10491711547877178 | No Hit |
| GACTACAAGGGTATCTAATCCTGTTTGCTCCCCACGCTTTCGCACCTCAG | 105 | 0.10491711547877178 | No Hit |
| GACTACACGGGTATCTAATCCTGTTTGCTCCCCACGCTTTCGCGNCTCAG | 104 | 0.10391790485516442 | No Hit |
| GAGACTACTCGGGTATCTAATCCTGTTTGCTCCCCACGCTTTCGCGCCTC | 102 | 0.10191948360794972 | No Hit |
| AGGACTACTGGGGTATCTAATCCTGTTTGCTCCCCACGCTTTCGCGCCTC | 101 | 0.10092027298434236 | No Hit |
| GACTACACGGGTATCTAATCCCGTTCGCTCCCCATGCTTTCGCACTCCAG | 101 | 0.10092027298434236 | No Hit |

## Adapter Content

Produced by FastQC (version 0.12.1)
