# Supplementary material for: Bacterial community data associated with Stephanitis pyri using 16S rRNA gene metabarcoding
Source: Data Brief. 2026 Jul 29;68:113107. doi: 10.1016/j.dib.2026.113107 (PMC13474393; doi:10.1016/j.dib.2026.113107)
Supplement: Supplementary file 1 [file mmc1.zip › supplementary_material_1/fastqc/c3-N4061-16Sv3v4_S24_L001_R1_001_fastqc.zip!/c3-N4061-16Sv3v4_S24_L001_R1_001_fastqc/fastqc_report.html]

c3-N4061-16Sv3v4\_S24\_L001\_R1\_001.fastq.gz FastQC Report 

FastQC Report

Fri 7 Nov 2025  
c3-N4061-16Sv3v4\_S24\_L001\_R1\_001.fastq.gz

## Summary

- Basic Statistics
- Per base sequence quality
- Per tile sequence quality
- Per sequence quality scores
- Per base sequence content
- Per sequence GC content
- Per base N content
- Sequence Length Distribution
- Sequence Duplication Levels
- Overrepresented sequences
- Adapter Content

## Basic Statistics

| Measure | Value |
| --- | --- |
| Filename | c3-N4061-16Sv3v4\_S24\_L001\_R1\_001.fastq.gz |
| File type | Conventional base calls |
| Encoding | Sanger / Illumina 1.9 |
| Total Sequences | 100219 |
| Total Bases | 30.1 Mbp |
| Sequences flagged as poor quality | 0 |
| Sequence length | 301 |
| %GC | 55 |

## Per base sequence quality

## Per tile sequence quality

## Per sequence quality scores

## Per base sequence content

## Per sequence GC content

## Per base N content

## Sequence Length Distribution

## Sequence Duplication Levels

## Overrepresented sequences

| Sequence | Count | Percentage | Possible Source |
| --- | --- | --- | --- |
| CCTACGGGTGGCAGCAGTGGGGAATATTGGACAATGGGCGCAAGCCTGAT | 4643 | 4.632854049631307 | No Hit |
| TCCTACGGGTGGCAGCAGTGGGGAATATTGGACAATGGGCGCAAGCCTGA | 2338 | 2.332890968778375 | No Hit |
| CCTACGGGAGGCAGCAGTGGGGAATATTGGACAATGGGCGCAAGCCTGAT | 2058 | 2.053502828804917 | No Hit |
| CCTACGGGTGGCTGCAGTGGGGAATATTGGACAATGGGCGCAAGCCTGAT | 1945 | 1.9407497580299145 | No Hit |
| CCTACGGGGGGCAGCAGTGGGGAATATTGGACAATGGGCGCAAGCCTGAT | 1782 | 1.7781059479739372 | No Hit |
| ACCTACGGGTGGCAGCAGTGGGGAATATTGGACAATGGGCGCAAGCCTGA | 1575 | 1.571558287350702 | No Hit |
| CCTACGGGAGGCTGCAGTGGGGAATATTGGACAATGGGCGCAAGCCTGAT | 1271 | 1.268222592522376 | No Hit |
| CCTACGGGCGGCAGCAGTGGGGAATATTGGACAATGGGCGCAAGCCTGAT | 1236 | 1.2332990750256936 | No Hit |
| GCCTACGGGTGGCAGCAGTGGGGAATATTGGACAATGGGCGCAAGCCTGA | 1196 | 1.1933864836009138 | No Hit |
| TCCTACGGGAGGCAGCAGTGGGGAATATTGGACAATGGGCGCAAGCCTGA | 1045 | 1.0427164509723705 | No Hit |
| CCCTACGGGTGGCAGCAGTGGGGAATATTGGACAATGGGCGCAAGCCTGA | 995 | 0.9928257116913959 | No Hit |
| CCTACGGGGGGCTGCAGTGGGGAATATTGGACAATGGGCGCAAGCCTGAT | 844 | 0.8421556790628523 | No Hit |
| TCCTACGGGTGGCTGCAGTGGGGAATATTGGACAATGGGCGCAAGCCTGA | 843 | 0.8411578642772328 | No Hit |
| TCCTACGGGGGGCAGCAGTGGGGAATATTGGACAATGGGCGCAAGCCTGA | 841 | 0.8391622347059939 | No Hit |
| TTCCTACGGGTGGCAGCAGTGGGGAATATTGGACAATGGGCGCAAGCCTG | 826 | 0.8241950129217015 | No Hit |
| ACCTACGGGAGGCAGCAGTGGGGAATATTGGACAATGGGCGCAAGCCTGA | 712 | 0.7104441273610792 | No Hit |
| GCCTACGGGAGGCAGCAGTGGGGAATATTGGACAATGGGCGCAAGCCTGA | 679 | 0.677516239435636 | No Hit |
| ACCTACGGGTGGCTGCAGTGGGGAATATTGGACAATGGGCGCAAGCCTGA | 669 | 0.6675380915794411 | No Hit |
| TCCTACGGGAGGCTGCAGTGGGGAATATTGGACAATGGGCGCAAGCCTGA | 667 | 0.665542462008202 | No Hit |
| ACCTACGGGGGGCAGCAGTGGGGAATATTGGACAATGGGCGCAAGCCTGA | 628 | 0.6266276853690418 | No Hit |
| TACCTACGGGTGGCAGCAGTGGGGAATATTGGACAATGGGCGCAAGCCTG | 557 | 0.5557828355900578 | No Hit |
| TCCTACGGGCGGCAGCAGTGGGGAATATTGGACAATGGGCGCAAGCCTGA | 542 | 0.5408156138057654 | No Hit |
| GCCTACGGGGGGCAGCAGTGGGGAATATTGGACAATGGGCGCAAGCCTGA | 513 | 0.5118789850228 | No Hit |
| CCTACGGGCGGCTGCAGTGGGGAATATTGGACAATGGGCGCAAGCCTGAT | 475 | 0.4739620231692593 | No Hit |
| ATCCTACGGGTGGCAGCAGTGGGGAATATTGGACAATGGGCGCAAGCCTG | 475 | 0.4739620231692593 | No Hit |
| GCCTACGGGTGGCTGCAGTGGGGAATATTGGACAATGGGCGCAAGCCTGA | 467 | 0.46597950488430334 | No Hit |
| CCCTACGGGAGGCAGCAGTGGGGAATATTGGACAATGGGCGCAAGCCTGA | 464 | 0.4629860605274449 | No Hit |
| CCCTACGGGTGGCTGCAGTGGGGAATATTGGACAATGGGCGCAAGCCTGA | 449 | 0.44801883874315246 | No Hit |
| GTCCTACGGGTGGCAGCAGTGGGGAATATTGGACAATGGGCGCAAGCCTG | 448 | 0.44702102395753296 | No Hit |
| ACCTACGGGAGGCTGCAGTGGGGAATATTGGACAATGGGCGCAAGCCTGA | 433 | 0.43205380217324063 | No Hit |
| CCTACGGGTGGCAGCAGTGGGGAATATTGGACAATGGGCGAAAGCCTGAT | 432 | 0.43105598738762113 | No Hit |
| AACCTACGGGTGGCAGCAGTGGGGAATATTGGACAATGGGCGCAAGCCTG | 413 | 0.41209750646085075 | No Hit |
| GCCTACGGGAGGCTGCAGTGGGGAATATTGGACAATGGGCGCAAGCCTGA | 401 | 0.40012372903341686 | No Hit |
| TCCCTACGGGTGGCAGCAGTGGGGAATATTGGACAATGGGCGCAAGCCTG | 392 | 0.39114339596284137 | No Hit |
| TTCCTACGGGAGGCAGCAGTGGGGAATATTGGACAATGGGCGCAAGCCTG | 375 | 0.37418054460731 | No Hit |
| TCCTACGGGGGGCTGCAGTGGGGAATATTGGACAATGGGCGCAAGCCTGA | 365 | 0.36420239675111504 | No Hit |
| TGCCTACGGGTGGCAGCAGTGGGGAATATTGGACAATGGGCGCAAGCCTG | 362 | 0.3612089523942566 | No Hit |
| ACCTACGGGCGGCAGCAGTGGGGAATATTGGACAATGGGCGCAAGCCTGA | 354 | 0.35322643410930066 | No Hit |
| CTCCTACGGGTGGCAGCAGTGGGGAATATTGGACAATGGGCGCAAGCCTG | 349 | 0.34823736018120316 | No Hit |
| TTCCTACGGGTGGCTGCAGTGGGGAATATTGGACAATGGGCGCAAGCCTG | 323 | 0.32229417575509633 | No Hit |
| AGCCTACGGGTGGCAGCAGTGGGGAATATTGGACAATGGGCGCAAGCCTG | 307 | 0.30632913918518445 | No Hit |
| CCCTACGGGGGGCAGCAGTGGGGAATATTGGACAATGGGCGCAAGCCTGA | 307 | 0.30632913918518445 | No Hit |
| GACCTACGGGTGGCAGCAGTGGGGAATATTGGACAATGGGCGCAAGCCTG | 301 | 0.3003422504714675 | No Hit |
| ACCTACGGGGGGCTGCAGTGGGGAATATTGGACAATGGGCGCAAGCCTGA | 294 | 0.293357546972131 | No Hit |
| GGCCTACGGGTGGCAGCAGTGGGGAATATTGGACAATGGGCGCAAGCCTG | 287 | 0.28637284347279457 | No Hit |
| CACCTACGGGTGGCAGCAGTGGGGAATATTGGACAATGGGCGCAAGCCTG | 285 | 0.2843772139015556 | No Hit |
| TTCCTACGGGGGGCAGCAGTGGGGAATATTGGACAATGGGCGCAAGCCTG | 281 | 0.2803859547590776 | No Hit |
| ACCCTACGGGTGGCAGCAGTGGGGAATATTGGACAATGGGCGCAAGCCTG | 280 | 0.2793881399734581 | No Hit |
| TACCTACGGGAGGCAGCAGTGGGGAATATTGGACAATGGGCGCAAGCCTG | 280 | 0.2793881399734581 | No Hit |
| CCCTACGGGAGGCTGCAGTGGGGAATATTGGACAATGGGCGCAAGCCTGA | 278 | 0.27739251040221913 | No Hit |
| GCCTACGGGCGGCAGCAGTGGGGAATATTGGACAATGGGCGCAAGCCTGA | 269 | 0.26841217733164374 | No Hit |
| TACCTACGGGTGGCTGCAGTGGGGAATATTGGACAATGGGCGCAAGCCTG | 240 | 0.23947554854867836 | No Hit |
| TACCTACGGGGGGCAGCAGTGGGGAATATTGGACAATGGGCGCAAGCCTG | 238 | 0.2374799189774394 | No Hit |
| GCCCTACGGGTGGCAGCAGTGGGGAATATTGGACAATGGGCGCAAGCCTG | 231 | 0.23049521547810295 | No Hit |
| TGCCTACGGGAGGCAGCAGTGGGGAATATTGGACAATGGGCGCAAGCCTG | 224 | 0.22351051197876648 | No Hit |
| ATCCTACGGGAGGCAGCAGTGGGGAATATTGGACAATGGGCGCAAGCCTG | 223 | 0.22251269719314704 | No Hit |
| TTTCCTACGGGTGGCAGCAGTGGGGAATATTGGACAATGGGCGCAAGCCT | 214 | 0.21353236412257154 | No Hit |
| GTCCTACGGGAGGCAGCAGTGGGGAATATTGGACAATGGGCGCAAGCCTG | 209 | 0.2085432901944741 | No Hit |
| CCCTACGGGCGGCAGCAGTGGGGAATATTGGACAATGGGCGCAAGCCTGA | 205 | 0.20455203105199612 | No Hit |
| TCCTACGGGTGGCAGCAGTGGGGAATATTGGACAATGGGCGAAAGCCTGA | 202 | 0.20155858669513765 | No Hit |
| GCCTACGGGGGGCTGCAGTGGGGAATATTGGACAATGGGCGCAAGCCTGA | 197 | 0.1965695127670402 | No Hit |
| TTCCTACGGGAGGCTGCAGTGGGGAATATTGGACAATGGGCGCAAGCCTG | 196 | 0.19557169798142068 | No Hit |
| TCCCTACGGGTGGCTGCAGTGGGGAATATTGGACAATGGGCGCAAGCCTG | 195 | 0.1945738831958012 | No Hit |
| TCCTACGGGCGGCTGCAGTGGGGAATATTGGACAATGGGCGCAAGCCTGA | 194 | 0.1935760684101817 | No Hit |
| TTACCTACGGGTGGCAGCAGTGGGGAATATTGGACAATGGGCGCAAGCCT | 191 | 0.1905826240533232 | No Hit |
| ATCCTACGGGTGGCTGCAGTGGGGAATATTGGACAATGGGCGCAAGCCTG | 188 | 0.18758917969646474 | No Hit |
| TTCCTACGGGCGGCAGCAGTGGGGAATATTGGACAATGGGCGCAAGCCTG | 187 | 0.18659136491084524 | No Hit |
| ATTCCTACGGGTGGCAGCAGTGGGGAATATTGGACAATGGGCGCAAGCCT | 186 | 0.18559355012522574 | No Hit |
| CCTACGGGTGGCTGCATTTGATACGTCTAGACTAGAGTGTGAGAGAGGGT | 186 | 0.18559355012522574 | No Hit |
| TATCCTACGGGTGGCAGCAGTGGGGAATATTGGACAATGGGCGCAAGCCT | 177 | 0.17661321705465033 | No Hit |
| ATCCTACGGGGGGCAGCAGTGGGGAATATTGGACAATGGGCGCAAGCCTG | 174 | 0.17361977269779183 | No Hit |
| AACCTACGGGTGGCTGCAGTGGGGAATATTGGACAATGGGCGCAAGCCTG | 174 | 0.17361977269779183 | No Hit |
| AACCTACGGGGGGCAGCAGTGGGGAATATTGGACAATGGGCGCAAGCCTG | 171 | 0.17062632834093336 | No Hit |
| CCTACGGGGGGCAGCAGTGGGGAATATTGGACAATGGGCGAAAGCCTGAT | 170 | 0.16962851355531386 | No Hit |
| CTCCTACGGGTGGCTGCAGTGGGGAATATTGGACAATGGGCGCAAGCCTG | 168 | 0.1676328839840749 | No Hit |
| TCCCTACGGGAGGCAGCAGTGGGGAATATTGGACAATGGGCGCAAGCCTG | 165 | 0.1646394396272164 | No Hit |
| CCTACGGGAGGCAGCAGTGGGGAATATTGGACAATGGGCGAAAGCCTGAT | 161 | 0.16064818048473842 | No Hit |
| GTCCTACGGGTGGCTGCAGTGGGGAATATTGGACAATGGGCGCAAGCCTG | 161 | 0.16064818048473842 | No Hit |
| TACCTACGGGAGGCTGCAGTGGGGAATATTGGACAATGGGCGCAAGCCTG | 161 | 0.16064818048473842 | No Hit |
| ATCCTACGGGAGGCTGCAGTGGGGAATATTGGACAATGGGCGCAAGCCTG | 158 | 0.15765473612787995 | No Hit |
| CTCCTACGGGAGGCAGCAGTGGGGAATATTGGACAATGGGCGCAAGCCTG | 157 | 0.15665692134226045 | No Hit |
| AGCCTACGGGAGGCAGCAGTGGGGAATATTGGACAATGGGCGCAAGCCTG | 155 | 0.15466129177102148 | No Hit |
| CCTACGGGTGGCAGCAGTGGGGAATCTTGGACAATGGGCGAAAGCCCGAT | 155 | 0.15466129177102148 | No Hit |
| GTCCTACGGGGGGCAGCAGTGGGGAATATTGGACAATGGGCGCAAGCCTG | 154 | 0.15366347698540198 | No Hit |
| GGCCTACGGGAGGCAGCAGTGGGGAATATTGGACAATGGGCGCAAGCCTG | 152 | 0.15166784741416298 | No Hit |
| AACCTACGGGAGGCAGCAGTGGGGAATATTGGACAATGGGCGCAAGCCTG | 148 | 0.147676588271685 | No Hit |
| CCCCTACGGGTGGCAGCAGTGGGGAATATTGGACAATGGGCGCAAGCCTG | 147 | 0.1466787734860655 | No Hit |
| CCTACGGGTGGCTGCAGTGGGGAATATTGGACAATGGGCGAAAGCCTGAT | 146 | 0.14568095870044603 | No Hit |
| TACCTACGGGCGGCAGCAGTGGGGAATATTGGACAATGGGCGCAAGCCTG | 146 | 0.14568095870044603 | No Hit |
| GACCTACGGGAGGCAGCAGTGGGGAATATTGGACAATGGGCGCAAGCCTG | 145 | 0.14468314391482653 | No Hit |
| GTCCTACGGGAGGCTGCAGTGGGGAATATTGGACAATGGGCGCAAGCCTG | 141 | 0.14069188477234856 | No Hit |
| ACCCTACGGGTGGCTGCAGTGGGGAATATTGGACAATGGGCGCAAGCCTG | 139 | 0.13869625520110956 | No Hit |
| ACCTACGGGTGGCAGCAGTGGGGAATATTGGACAATGGGCGAAAGCCTGA | 139 | 0.13869625520110956 | No Hit |
| TGCCTACGGGGGGCAGCAGTGGGGAATATTGGACAATGGGCGCAAGCCTG | 137 | 0.1367006256298706 | No Hit |
| TAACCTACGGGTGGCAGCAGTGGGGAATATTGGACAATGGGCGCAAGCCT | 135 | 0.1347049960586316 | No Hit |
| AATCCTACGGGTGGCAGCAGTGGGGAATATTGGACAATGGGCGCAAGCCT | 135 | 0.1347049960586316 | No Hit |
| GACCTACGGGGGGCAGCAGTGGGGAATATTGGACAATGGGCGCAAGCCTG | 134 | 0.1337071812730121 | No Hit |
| TTCCTACGGGGGGCTGCAGTGGGGAATATTGGACAATGGGCGCAAGCCTG | 132 | 0.13171155170177312 | No Hit |
| ACCTACGGGCGGCTGCAGTGGGGAATATTGGACAATGGGCGCAAGCCTGA | 130 | 0.12971592213053412 | No Hit |
| AACCTACGGGAGGCTGCAGTGGGGAATATTGGACAATGGGCGCAAGCCTG | 128 | 0.12772029255929515 | No Hit |
| TGCCTACGGGTGGCTGCAGTGGGGAATATTGGACAATGGGCGCAAGCCTG | 128 | 0.12772029255929515 | No Hit |
| TTCCCTACGGGTGGCAGCAGTGGGGAATATTGGACAATGGGCGCAAGCCT | 128 | 0.12772029255929515 | No Hit |
| CACCTACGGGTGGCTGCAGTGGGGAATATTGGACAATGGGCGCAAGCCTG | 128 | 0.12772029255929515 | No Hit |
| TCCCTACGGGGGGCAGCAGTGGGGAATATTGGACAATGGGCGCAAGCCTG | 127 | 0.12672247777367565 | No Hit |
| TGCCTACGGGAGGCTGCAGTGGGGAATATTGGACAATGGGCGCAAGCCTG | 125 | 0.12472684820243665 | No Hit |
| AGCCTACGGGTGGCTGCAGTGGGGAATATTGGACAATGGGCGCAAGCCTG | 122 | 0.12173340384557818 | No Hit |
| AGCCTACGGGGGGCAGCAGTGGGGAATATTGGACAATGGGCGCAAGCCTG | 122 | 0.12173340384557818 | No Hit |
| CGCCTACGGGTGGCAGCAGTGGGGAATATTGGACAATGGGCGCAAGCCTG | 121 | 0.1207355890599587 | No Hit |
| ACCCTACGGGAGGCAGCAGTGGGGAATATTGGACAATGGGCGCAAGCCTG | 121 | 0.1207355890599587 | No Hit |
| CACCTACGGGAGGCAGCAGTGGGGAATATTGGACAATGGGCGCAAGCCTG | 119 | 0.1187399594887197 | No Hit |
| ATCCTACGGGCGGCAGCAGTGGGGAATATTGGACAATGGGCGCAAGCCTG | 118 | 0.11774214470310021 | No Hit |
| ATACCTACGGGTGGCAGCAGTGGGGAATATTGGACAATGGGCGCAAGCCT | 116 | 0.11574651513186122 | No Hit |
| TGTCCTACGGGTGGCAGCAGTGGGGAATATTGGACAATGGGCGCAAGCCT | 116 | 0.11574651513186122 | No Hit |
| TACCCTACGGGTGGCAGCAGTGGGGAATATTGGACAATGGGCGCAAGCCT | 116 | 0.11574651513186122 | No Hit |
| CCTACGGGTGGCTGCATTTCAAACTGGGAGTCTAGAGTGCAGGAGAGGAG | 113 | 0.11275307077500275 | No Hit |
| AACCTACGGGCGGCAGCAGTGGGGAATATTGGACAATGGGCGCAAGCCTG | 111 | 0.11075744120376375 | No Hit |
| CCTACGGGCGGCAGCAGTGGGGAATATTGGACAATGGGCGAAAGCCTGAT | 111 | 0.11075744120376375 | No Hit |
| CCTACGGGAGGCTGCAGTGGGGAATATTGGACAATGGGCGAAAGCCTGAT | 110 | 0.10975962641814427 | No Hit |
| CCTACGGGGGGCTGCATTTGATACGTCTAGACTAGAGTGTGAGAGAGGGT | 110 | 0.10975962641814427 | No Hit |
| GTTCCTACGGGTGGCAGCAGTGGGGAATATTGGACAATGGGCGCAAGCCT | 108 | 0.10776399684690528 | No Hit |
| GACCTACGGGTGGCTGCAGTGGGGAATATTGGACAATGGGCGCAAGCCTG | 107 | 0.10676618206128577 | No Hit |
| ACCCTACGGGGGGCAGCAGTGGGGAATATTGGACAATGGGCGCAAGCCTG | 105 | 0.1047705524900468 | No Hit |
| GCCCTACGGGAGGCAGCAGTGGGGAATATTGGACAATGGGCGCAAGCCTG | 105 | 0.1047705524900468 | No Hit |
| TTTCCTACGGGAGGCAGCAGTGGGGAATATTGGACAATGGGCGCAAGCCT | 104 | 0.1037727377044273 | No Hit |
| GTCCTACGGGCGGCAGCAGTGGGGAATATTGGACAATGGGCGCAAGCCTG | 103 | 0.10277492291880781 | No Hit |
| TCCTACGGGAGGCAGCAGTGGGGAATATTGGACAATGGGCGAAAGCCTGA | 103 | 0.10277492291880781 | No Hit |
| ATCCCTACGGGTGGCAGCAGTGGGGAATATTGGACAATGGGCGCAAGCCT | 103 | 0.10277492291880781 | No Hit |
| GGCCTACGGGTGGCTGCAGTGGGGAATATTGGACAATGGGCGCAAGCCTG | 103 | 0.10277492291880781 | No Hit |
| GCCTACGGGTGGCAGCAGTGGGGAATATTGGACAATGGGCGAAAGCCTGA | 101 | 0.10077929334756883 | No Hit |

## Adapter Content

Produced by FastQC (version 0.12.1)
