# Supplementary material for: Bacterial community data associated with Stephanitis pyri using 16S rRNA gene metabarcoding
Source: Data Brief. 2026 Jul 29;68:113107. doi: 10.1016/j.dib.2026.113107 (PMC13474393; doi:10.1016/j.dib.2026.113107)
Supplement: Supplementary file 1 [file mmc1.zip › supplementary_material_1/fastqc/c3-N4061-16Sv3v4_S24_L001_R2_001_fastqc.zip!/c3-N4061-16Sv3v4_S24_L001_R2_001_fastqc/fastqc_report.html]

c3-N4061-16Sv3v4\_S24\_L001\_R2\_001.fastq.gz FastQC Report 

FastQC Report

Fri 7 Nov 2025  
c3-N4061-16Sv3v4\_S24\_L001\_R2\_001.fastq.gz

## Summary

- Basic Statistics
- Per base sequence quality
- Per tile sequence quality
- Per sequence quality scores
- Per base sequence content
- Per sequence GC content
- Per base N content
- Sequence Length Distribution
- Sequence Duplication Levels
- Overrepresented sequences
- Adapter Content

## Basic Statistics

| Measure | Value |
| --- | --- |
| Filename | c3-N4061-16Sv3v4\_S24\_L001\_R2\_001.fastq.gz |
| File type | Conventional base calls |
| Encoding | Sanger / Illumina 1.9 |
| Total Sequences | 100219 |
| Total Bases | 30.1 Mbp |
| Sequences flagged as poor quality | 0 |
| Sequence length | 301 |
| %GC | 54 |

## Per base sequence quality

## Per tile sequence quality

## Per sequence quality scores

## Per base sequence content

## Per sequence GC content

## Per base N content

## Sequence Length Distribution

## Sequence Duplication Levels

## Overrepresented sequences

| Sequence | Count | Percentage | Possible Source |
| --- | --- | --- | --- |
| GACTACTAGGGTATCTAATCCTGTTTGCTCCCCACGCTTTCGCGCCTCAG | 2756 | 2.7499775491673235 | No Hit |
| GACTACTCGGGTATCTAATCCTGTTTGCTCCCCACGCTTTCGCGCCTCAG | 2331 | 2.325906265279039 | No Hit |
| GACTACTGGGGTATCTAATCCTGTTTGCTCCCCACGCTTTCGCGCCTCAG | 2087 | 2.0824394575878826 | No Hit |
| GACTACAAGGGTATCTAATCCTGTTTGCTCCCCACGCTTTCGCGCCTCAG | 1925 | 1.9207934623175247 | No Hit |
| GACTACCAGGGTATCTAATCCTGTTTGCTCCCCACGCTTTCGCGCCTCAG | 1729 | 1.7252217643361039 | No Hit |
| GACTACACGGGTATCTAATCCTGTTTGCTCCCCACGCTTTCGCGCCTCAG | 1626 | 1.6224468414172961 | No Hit |
| GACTACAGGGGTATCTAATCCTGTTTGCTCCCCACGCTTTCGCGCCTCAG | 1516 | 1.512687214999152 | No Hit |
| GACTACCGGGGTATCTAATCCTGTTTGCTCCCCACGCTTTCGCGCCTCAG | 1191 | 1.1883974096728165 | No Hit |
| GACTACCCGGGTATCTAATCCTGTTTGCTCCCCACGCTTTCGCGCCTCAG | 1072 | 1.0696574501840967 | No Hit |
| TGACTACTAGGGTATCTAATCCTGTTTGCTCCCCACGCTTTCGCGCCTCA | 861 | 0.8591185304183837 | No Hit |
| TGACTACTCGGGTATCTAATCCTGTTTGCTCCCCACGCTTTCGCGCCTCA | 751 | 0.7493589040002395 | No Hit |
| TGACTACAAGGGTATCTAATCCTGTTTGCTCCCCACGCTTTCGCGCCTCA | 678 | 0.6765184246500164 | No Hit |
| TGACTACTGGGGTATCTAATCCTGTTTGCTCCCCACGCTTTCGCGCCTCA | 625 | 0.6236342410121833 | No Hit |
| AGACTACTAGGGTATCTAATCCTGTTTGCTCCCCACGCTTTCGCGCCTCA | 593 | 0.5917041678723595 | No Hit |
| TGACTACCAGGGTATCTAATCCTGTTTGCTCCCCACGCTTTCGCGCCTCA | 560 | 0.5587762799469163 | No Hit |
| CGACTACTAGGGTATCTAATCCTGTTTGCTCCCCACGCTTTCGCGCCTCA | 529 | 0.5278440215927119 | No Hit |
| GGACTACTAGGGTATCTAATCCTGTTTGCTCCCCACGCTTTCGCGCCTCA | 528 | 0.5268462068070925 | No Hit |
| AGACTACAAGGGTATCTAATCCTGTTTGCTCCCCACGCTTTCGCGCCTCA | 498 | 0.4969117632385077 | No Hit |
| TGACTACAGGGGTATCTAATCCTGTTTGCTCCCCACGCTTTCGCGCCTCA | 479 | 0.47795328231173734 | No Hit |
| AGACTACTCGGGTATCTAATCCTGTTTGCTCCCCACGCTTTCGCGCCTCA | 471 | 0.46997076402678134 | No Hit |
| GGACTACTGGGGTATCTAATCCTGTTTGCTCCCCACGCTTTCGCGCCTCA | 457 | 0.45600135702810846 | No Hit |
| GGACTACTCGGGTATCTAATCCTGTTTGCTCCCCACGCTTTCGCGCCTCA | 446 | 0.44502539438629407 | No Hit |
| TGACTACACGGGTATCTAATCCTGTTTGCTCCCCACGCTTTCGCGCCTCA | 444 | 0.443029764815055 | No Hit |
| AGACTACCAGGGTATCTAATCCTGTTTGCTCCCCACGCTTTCGCGCCTCA | 422 | 0.42107783953142613 | No Hit |
| GGACTACAAGGGTATCTAATCCTGTTTGCTCCCCACGCTTTCGCGCCTCA | 421 | 0.42008002474580675 | No Hit |
| GGACTACCAGGGTATCTAATCCTGTTTGCTCCCCACGCTTTCGCGCCTCA | 411 | 0.41010187688961175 | No Hit |
| TGACTACCCGGGTATCTAATCCTGTTTGCTCCCCACGCTTTCGCGCCTCA | 407 | 0.4061106177471338 | No Hit |
| TGACTACCGGGGTATCTAATCCTGTTTGCTCCCCACGCTTTCGCGCCTCA | 405 | 0.4041149881758948 | No Hit |
| CGACTACCAGGGTATCTAATCCTGTTTGCTCCCCACGCTTTCGCGCCTCA | 400 | 0.39912591424779736 | No Hit |
| CGACTACTCGGGTATCTAATCCTGTTTGCTCCCCACGCTTTCGCGCCTCA | 393 | 0.39214121074846087 | No Hit |
| AGACTACTGGGGTATCTAATCCTGTTTGCTCCCCACGCTTTCGCGCCTCA | 390 | 0.3891477663916024 | No Hit |
| CGACTACAAGGGTATCTAATCCTGTTTGCTCCCCACGCTTTCGCGCCTCA | 386 | 0.3851565072491244 | No Hit |
| CGACTACTGGGGTATCTAATCCTGTTTGCTCCCCACGCTTTCGCGCCTCA | 378 | 0.3771739889641685 | No Hit |
| AGACTACAGGGGTATCTAATCCTGTTTGCTCCCCACGCTTTCGCGCCTCA | 372 | 0.3711871002504515 | No Hit |
| AGACTACACGGGTATCTAATCCTGTTTGCTCCCCACGCTTTCGCGCCTCA | 369 | 0.36819365589359304 | No Hit |
| GGACTACACGGGTATCTAATCCTGTTTGCTCCCCACGCTTTCGCGCCTCA | 354 | 0.35322643410930066 | No Hit |
| GGACTACAGGGGTATCTAATCCTGTTTGCTCCCCACGCTTTCGCGCCTCA | 347 | 0.3462417306099642 | No Hit |
| GGACTACCGGGGTATCTAATCCTGTTTGCTCCCCACGCTTTCGCGCCTCA | 323 | 0.32229417575509633 | No Hit |
| AGACTACCGGGGTATCTAATCCTGTTTGCTCCCCACGCTTTCGCGCCTCA | 317 | 0.3163072870413794 | No Hit |
| TTGACTACTAGGGTATCTAATCCTGTTTGCTCCCCACGCTTTCGCGCCTC | 295 | 0.29435536175775057 | No Hit |
| AGACTACCCGGGTATCTAATCCTGTTTGCTCCCCACGCTTTCGCGCCTCA | 287 | 0.28637284347279457 | No Hit |
| CGACTACAGGGGTATCTAATCCTGTTTGCTCCCCACGCTTTCGCGCCTCA | 283 | 0.28238158433031657 | No Hit |
| CGACTACACGGGTATCTAATCCTGTTTGCTCCCCACGCTTTCGCGCCTCA | 269 | 0.26841217733164374 | No Hit |
| TTGACTACTCGGGTATCTAATCCTGTTTGCTCCCCACGCTTTCGCGCCTC | 247 | 0.24646025204801483 | No Hit |
| CGACTACCCGGGTATCTAATCCTGTTTGCTCCCCACGCTTTCGCGCCTCA | 217 | 0.21652580847943007 | No Hit |
| ATGACTACTAGGGTATCTAATCCTGTTTGCTCCCCACGCTTTCGCGCCTC | 209 | 0.2085432901944741 | No Hit |
| TTGACTACTGGGGTATCTAATCCTGTTTGCTCCCCACGCTTTCGCGCCTC | 209 | 0.2085432901944741 | No Hit |
| TTGACTACCAGGGTATCTAATCCTGTTTGCTCCCCACGCTTTCGCGCCTC | 206 | 0.20554984583761562 | No Hit |
| GACTACTAGGGTATCTAATCCTGTTTGCTCCCCACGCTTTCGCACCTCAG | 204 | 0.20355421626637665 | No Hit |
| GACTACTAGGGTATCTAATCCTGTTTGCTCCCCACGCTTTCGCGNCTCAG | 202 | 0.20155858669513765 | No Hit |
| GACTACAAGGGTATCTAATCCTGTTTGCTCCCCACGCTTTCGCACCTCAG | 194 | 0.1935760684101817 | No Hit |
| GGACTACCCGGGTATCTAATCCTGTTTGCTCCCCACGCTTTCGCGCCTCA | 192 | 0.1915804388389427 | No Hit |
| TGGACTACTAGGGTATCTAATCCTGTTTGCTCCCCACGCTTTCGCGCCTC | 187 | 0.18659136491084524 | No Hit |
| GACTACTCGGGTATCTAATCCTGTTTGCTCCCCACGCTTTCGCACCTCAG | 187 | 0.18659136491084524 | No Hit |
| GTGACTACTAGGGTATCTAATCCTGTTTGCTCCCCACGCTTTCGCGCCTC | 184 | 0.18359792055398677 | No Hit |
| TAGACTACTCGGGTATCTAATCCTGTTTGCTCCCCACGCTTTCGCGCCTC | 174 | 0.17361977269779183 | No Hit |
| ATGACTACTGGGGTATCTAATCCTGTTTGCTCCCCACGCTTTCGCGCCTC | 173 | 0.17262195791217233 | No Hit |
| TTGACTACAGGGGTATCTAATCCTGTTTGCTCCCCACGCTTTCGCGCCTC | 170 | 0.16962851355531386 | No Hit |
| TAGACTACTAGGGTATCTAATCCTGTTTGCTCCCCACGCTTTCGCGCCTC | 162 | 0.16164599527035792 | No Hit |
| GTGACTACTGGGGTATCTAATCCTGTTTGCTCCCCACGCTTTCGCGCCTC | 161 | 0.16064818048473842 | No Hit |
| GACTACACGGGTATCTAATCCTGTTTGCTCCCCACGCTTTCGCACCTCAG | 159 | 0.15865255091349945 | No Hit |
| ATGACTACTCGGGTATCTAATCCTGTTTGCTCCCCACGCTTTCGCGCCTC | 158 | 0.15765473612787995 | No Hit |
| TTGACTACACGGGTATCTAATCCTGTTTGCTCCCCACGCTTTCGCGCCTC | 156 | 0.15565910655664095 | No Hit |
| CGACTACCGGGGTATCTAATCCTGTTTGCTCCCCACGCTTTCGCGCCTCA | 154 | 0.15366347698540198 | No Hit |
| GTGACTACTCGGGTATCTAATCCTGTTTGCTCCCCACGCTTTCGCGCCTC | 153 | 0.15266566219978248 | No Hit |
| TGGACTACTCGGGTATCTAATCCTGTTTGCTCCCCACGCTTTCGCGCCTC | 153 | 0.15266566219978248 | No Hit |
| GACTACTGGGGTATCTAATCCTGTTTGCTCCCCACGCTTTCGCACCTCAG | 152 | 0.15166784741416298 | No Hit |
| TCGACTACTAGGGTATCTAATCCTGTTTGCTCCCCACGCTTTCGCGCCTC | 152 | 0.15166784741416298 | No Hit |
| GACTACAGGGGTATCTAATCCTGTTTGCTCCCCACGCTTTCGCACCTCAG | 151 | 0.15067003262854348 | No Hit |
| ATGACTACAAGGGTATCTAATCCTGTTTGCTCCCCACGCTTTCGCGCCTC | 147 | 0.1466787734860655 | No Hit |
| TAGACTACTGGGGTATCTAATCCTGTTTGCTCCCCACGCTTTCGCGCCTC | 147 | 0.1466787734860655 | No Hit |
| TAGACTACAAGGGTATCTAATCCTGTTTGCTCCCCACGCTTTCGCGCCTC | 147 | 0.1466787734860655 | No Hit |
| GTGACTACAAGGGTATCTAATCCTGTTTGCTCCCCACGCTTTCGCGCCTC | 144 | 0.14368532912920703 | No Hit |
| GACTACTCGGGTATCTAATCCTGTTTGCTCCCCACGCTTTCGCGNCTCAG | 143 | 0.14268751434358753 | No Hit |
| CTGACTACTAGGGTATCTAATCCTGTTTGCTCCCCACGCTTTCGCGCCTC | 140 | 0.13969406998672906 | No Hit |
| CTGACTACTGGGGTATCTAATCCTGTTTGCTCCCCACGCTTTCGCGCCTC | 138 | 0.13769844041549006 | No Hit |
| GACTACTGGGGTATCTAATCCTGTTTGCTCCCCACGCTTTCGCGNCTCAG | 138 | 0.13769844041549006 | No Hit |
| AAGACTACTAGGGTATCTAATCCTGTTTGCTCCCCACGCTTTCGCGCCTC | 134 | 0.1337071812730121 | No Hit |
| TGGACTACAAGGGTATCTAATCCTGTTTGCTCCCCACGCTTTCGCGCCTC | 133 | 0.13270936648739262 | No Hit |
| GACTACCAGGGTATCTAATCCTGTTTGCTCCCCACGCTTTCGCACCTCAG | 133 | 0.13270936648739262 | No Hit |
| CTGACTACTCGGGTATCTAATCCTGTTTGCTCCCCACGCTTTCGCGCCTC | 131 | 0.13071373691615362 | No Hit |
| GGGACTACTAGGGTATCTAATCCTGTTTGCTCCCCACGCTTTCGCGCCTC | 128 | 0.12772029255929515 | No Hit |
| TGGACTACTGGGGTATCTAATCCTGTTTGCTCCCCACGCTTTCGCGCCTC | 128 | 0.12772029255929515 | No Hit |
| TCGACTACTCGGGTATCTAATCCTGTTTGCTCCCCACGCTTTCGCGCCTC | 127 | 0.12672247777367565 | No Hit |
| GGGGGGGGGGGGGGGGGGGGGGGGGGGGGGGGGGGGGGGGGGGGGGGGGG | 124 | 0.12372903341681717 | No Hit |
| TTGACTACCGGGGTATCTAATCCTGTTTGCTCCCCACGCTTTCGCGCCTC | 124 | 0.12372903341681717 | No Hit |
| TAGACTACCAGGGTATCTAATCCTGTTTGCTCCCCACGCTTTCGCGCCTC | 124 | 0.12372903341681717 | No Hit |
| GACTACAAGGGTATCTAATCCTGTTTGCTCCCCACGCTTTCGCGNCTCAG | 122 | 0.12173340384557818 | No Hit |
| TTGACTACCCGGGTATCTAATCCTGTTTGCTCCCCACGCTTTCGCGCCTC | 121 | 0.1207355890599587 | No Hit |
| GTGACTACCAGGGTATCTAATCCTGTTTGCTCCCCACGCTTTCGCGCCTC | 121 | 0.1207355890599587 | No Hit |
| TTGACTACAAGGGTATCTAATCCTGTTTGCTCCCCACGCTTTCGCGCCTC | 121 | 0.1207355890599587 | No Hit |
| AGGACTACTAGGGTATCTAATCCTGTTTGCTCCCCACGCTTTCGCGCCTC | 119 | 0.1187399594887197 | No Hit |
| CTGACTACAAGGGTATCTAATCCTGTTTGCTCCCCACGCTTTCGCGCCTC | 118 | 0.11774214470310021 | No Hit |
| TAGACTACAGGGGTATCTAATCCTGTTTGCTCCCCACGCTTTCGCGCCTC | 117 | 0.11674432991748071 | No Hit |
| CAGACTACTAGGGTATCTAATCCTGTTTGCTCCCCACGCTTTCGCGCCTC | 117 | 0.11674432991748071 | No Hit |
| GCGACTACTAGGGTATCTAATCCTGTTTGCTCCCCACGCTTTCGCGCCTC | 116 | 0.11574651513186122 | No Hit |
| CGGACTACTAGGGTATCTAATCCTGTTTGCTCCCCACGCTTTCGCGCCTC | 115 | 0.11474870034624174 | No Hit |
| GACTACACGGGTATCTAATCCTGTTTGCTCCCCACGCTTTCGCGNCTCAG | 115 | 0.11474870034624174 | No Hit |
| TGACTACTAGGGTATCTAATCCTGTTTGCTCCCCACGCTTTCGCACCTCA | 114 | 0.11375088556062224 | No Hit |
| TCGACTACTGGGGTATCTAATCCTGTTTGCTCCCCACGCTTTCGCGCCTC | 114 | 0.11375088556062224 | No Hit |
| GACTACCCGGGTATCTAATCCTGTTTGCTCCCCACGCTTTCGCACCTCAG | 114 | 0.11375088556062224 | No Hit |
| ATGACTACACGGGTATCTAATCCTGTTTGCTCCCCACGCTTTCGCGCCTC | 114 | 0.11375088556062224 | No Hit |
| GAGACTACTAGGGTATCTAATCCTGTTTGCTCCCCACGCTTTCGCGCCTC | 113 | 0.11275307077500275 | No Hit |
| ATGACTACAGGGGTATCTAATCCTGTTTGCTCCCCACGCTTTCGCGCCTC | 113 | 0.11275307077500275 | No Hit |
| GAGACTACTCGGGTATCTAATCCTGTTTGCTCCCCACGCTTTCGCGCCTC | 112 | 0.11175525598938324 | No Hit |
| TCGACTACAAGGGTATCTAATCCTGTTTGCTCCCCACGCTTTCGCGCCTC | 110 | 0.10975962641814427 | No Hit |
| GACTACCGGGGTATCTAATCCTGTTTGCTCCCCACGCTTTCGCACCTCAG | 108 | 0.10776399684690528 | No Hit |
| TGGACTACAGGGGTATCTAATCCTGTTTGCTCCCCACGCTTTCGCGCCTC | 108 | 0.10776399684690528 | No Hit |
| GACTACCAGGGTATCTAATCCTGTTTGCTCCCCACGCTTTCGCGNCTCAG | 107 | 0.10676618206128577 | No Hit |
| TAGACTACACGGGTATCTAATCCTGTTTGCTCCCCACGCTTTCGCGCCTC | 106 | 0.10576836727566628 | No Hit |
| ACGACTACAAGGGTATCTAATCCTGTTTGCTCCCCACGCTTTCGCGCCTC | 106 | 0.10576836727566628 | No Hit |
| CAGACTACTCGGGTATCTAATCCTGTTTGCTCCCCACGCTTTCGCGCCTC | 105 | 0.1047705524900468 | No Hit |
| AAGACTACTCGGGTATCTAATCCTGTTTGCTCCCCACGCTTTCGCGCCTC | 105 | 0.1047705524900468 | No Hit |
| GGGACTACTCGGGTATCTAATCCTGTTTGCTCCCCACGCTTTCGCGCCTC | 104 | 0.1037727377044273 | No Hit |
| CTGACTACCAGGGTATCTAATCCTGTTTGCTCCCCACGCTTTCGCGCCTC | 104 | 0.1037727377044273 | No Hit |
| TCGACTACAGGGGTATCTAATCCTGTTTGCTCCCCACGCTTTCGCGCCTC | 103 | 0.10277492291880781 | No Hit |
| GAGACTACTGGGGTATCTAATCCTGTTTGCTCCCCACGCTTTCGCGCCTC | 102 | 0.10177710813318833 | No Hit |
| CGGACTACTGGGGTATCTAATCCTGTTTGCTCCCCACGCTTTCGCGCCTC | 102 | 0.10177710813318833 | No Hit |
| AGGACTACTGGGGTATCTAATCCTGTTTGCTCCCCACGCTTTCGCGCCTC | 101 | 0.10077929334756883 | No Hit |

## Adapter Content

Produced by FastQC (version 0.12.1)
