# Supplementary material for: Bacterial community data associated with Stephanitis pyri using 16S rRNA gene metabarcoding
Source: Data Brief. 2026 Jul 29;68:113107. doi: 10.1016/j.dib.2026.113107 (PMC13474393; doi:10.1016/j.dib.2026.113107)
Supplement: Supplementary file 1 [file mmc1.zip › supplementary_material_1/fastqc/c4-N4062-16Sv3v4_S52_L001_R1_001_fastqc.zip!/c4-N4062-16Sv3v4_S52_L001_R1_001_fastqc/fastqc_report.html]

c4-N4062-16Sv3v4\_S52\_L001\_R1\_001.fastq.gz FastQC Report 

FastQC Report

Mon 13 Oct 2025  
c4-N4062-16Sv3v4\_S52\_L001\_R1\_001.fastq.gz

## Summary

- Basic Statistics
- Per base sequence quality
- Per tile sequence quality
- Per sequence quality scores
- Per base sequence content
- Per sequence GC content
- Per base N content
- Sequence Length Distribution
- Sequence Duplication Levels
- Overrepresented sequences
- Adapter Content

## Basic Statistics

| Measure | Value |
| --- | --- |
| Filename | c4-N4062-16Sv3v4\_S52\_L001\_R1\_001.fastq.gz |
| File type | Conventional base calls |
| Encoding | Sanger / Illumina 1.9 |
| Total Sequences | 53837 |
| Total Bases | 16.2 Mbp |
| Sequences flagged as poor quality | 0 |
| Sequence length | 301 |
| %GC | 55 |

## Per base sequence quality

## Per tile sequence quality

## Per sequence quality scores

## Per base sequence content

## Per sequence GC content

## Per base N content

## Sequence Length Distribution

## Sequence Duplication Levels

## Overrepresented sequences

| Sequence | Count | Percentage | Possible Source |
| --- | --- | --- | --- |
| CCTACGGGTGGCAGCAGTGGGGAATATTGGACAATGGGCGCAAGCCTGAT | 2271 | 4.218288537622825 | No Hit |
| CCTACGGGAGGCAGCAGTGGGGAATATTGGACAATGGGCGCAAGCCTGAT | 964 | 1.790590114605197 | No Hit |
| CCTACGGGTGGCTGCAGTGGGGAATATTGGACAATGGGCGCAAGCCTGAT | 925 | 1.7181492282259414 | No Hit |
| CCTACGGGGGGCAGCAGTGGGGAATATTGGACAATGGGCGCAAGCCTGAT | 925 | 1.7181492282259414 | No Hit |
| TCCTACGGGTGGCAGCAGTGGGGAATATTGGACAATGGGCGCAAGCCTGA | 808 | 1.5008265690881735 | No Hit |
| ACCTACGGGTGGCAGCAGTGGGGAATATTGGACAATGGGCGCAAGCCTGA | 764 | 1.419098389583372 | No Hit |
| CCTACGGGAGGCTGCAGTGGGGAATATTGGACAATGGGCGCAAGCCTGAT | 620 | 1.151624347567658 | No Hit |
| GCCTACGGGTGGCAGCAGTGGGGAATATTGGACAATGGGCGCAAGCCTGA | 607 | 1.1274773854412394 | No Hit |
| CCTACGGGCGGCAGCAGTGGGGAATATTGGACAATGGGCGCAAGCCTGAT | 511 | 0.9491613574307632 | No Hit |
| CCTACGGGGGGCTGCAGTGGGGAATATTGGACAATGGGCGCAAGCCTGAT | 405 | 0.752270743169196 | No Hit |
| CCTACGGGTGGCAGCAGTGGGGAATATTGGACAATGGGCGAAAGCCTGAT | 403 | 0.7485558259189776 | No Hit |
| TCCTACGGGAGGCAGCAGTGGGGAATATTGGACAATGGGCGCAAGCCTGA | 383 | 0.7114066534167952 | No Hit |
| TCCTACGGGTGGCTGCAGTGGGGAATATTGGACAATGGGCGCAAGCCTGA | 369 | 0.6854022326652675 | No Hit |
| ACCTACGGGTGGCTGCAGTGGGGAATATTGGACAATGGGCGCAAGCCTGA | 367 | 0.6816873154150491 | No Hit |
| TTCCTACGGGTGGCAGCAGTGGGGAATATTGGACAATGGGCGCAAGCCTG | 356 | 0.6612552705388487 | No Hit |
| ACCTACGGGGGGCAGCAGTGGGGAATATTGGACAATGGGCGCAAGCCTGA | 348 | 0.6463956015379757 | No Hit |
| ACCTACGGGAGGCAGCAGTGGGGAATATTGGACAATGGGCGCAAGCCTGA | 326 | 0.605531511785575 | No Hit |
| GCCTACGGGAGGCAGCAGTGGGGAATATTGGACAATGGGCGCAAGCCTGA | 321 | 0.5962442186600293 | No Hit |
| CCCTACGGGTGGCAGCAGTGGGGAATATTGGACAATGGGCGCAAGCCTGA | 317 | 0.5888143841595929 | No Hit |
| TCCTACGGGGGGCAGCAGTGGGGAATATTGGACAATGGGCGCAAGCCTGA | 291 | 0.5405204599067556 | No Hit |
| TACCTACGGGTGGCAGCAGTGGGGAATATTGGACAATGGGCGCAAGCCTG | 285 | 0.5293757081561008 | No Hit |
| TCCTACGGGAGGCTGCAGTGGGGAATATTGGACAATGGGCGCAAGCCTGA | 256 | 0.4755094080279362 | No Hit |
| ATCCTACGGGTGGCAGCAGTGGGGAATATTGGACAATGGGCGCAAGCCTG | 237 | 0.44021769415086276 | No Hit |
| CCTACGGGCGGCTGCAGTGGGGAATATTGGACAATGGGCGCAAGCCTGAT | 236 | 0.43836023552575365 | No Hit |
| ACCTACGGGAGGCTGCAGTGGGGAATATTGGACAATGGGCGCAAGCCTGA | 224 | 0.41607073202444417 | No Hit |
| GCCTACGGGTGGCTGCAGTGGGGAATATTGGACAATGGGCGCAAGCCTGA | 213 | 0.39563868714824374 | No Hit |
| AACCTACGGGTGGCAGCAGTGGGGAATATTGGACAATGGGCGCAAGCCTG | 208 | 0.38635139402269814 | No Hit |
| GCCTACGGGGGGCAGCAGTGGGGAATATTGGACAATGGGCGCAAGCCTGA | 204 | 0.37892155952226164 | No Hit |
| GCCTACGGGAGGCTGCAGTGGGGAATATTGGACAATGGGCGCAAGCCTGA | 201 | 0.37334918364693426 | No Hit |
| GTCCTACGGGTGGCAGCAGTGGGGAATATTGGACAATGGGCGCAAGCCTG | 199 | 0.369634266396716 | No Hit |
| CTCCTACGGGTGGCAGCAGTGGGGAATATTGGACAATGGGCGCAAGCCTG | 191 | 0.354774597395843 | No Hit |
| TGCCTACGGGTGGCAGCAGTGGGGAATATTGGACAATGGGCGCAAGCCTG | 188 | 0.3492022215205156 | No Hit |
| TTCCTACGGGAGGCAGCAGTGGGGAATATTGGACAATGGGCGCAAGCCTG | 185 | 0.3436298456451882 | No Hit |
| TCCTACGGGGGGCTGCAGTGGGGAATATTGGACAATGGGCGCAAGCCTGA | 178 | 0.33062763526942435 | No Hit |
| CCTACGGGTGGCTGCAGTGGGGAATATTGGACAATGGGCGAAAGCCTGAT | 178 | 0.33062763526942435 | No Hit |
| CCTACGGGGGGCAGCAGTGGGGAATATTGGACAATGGGCGAAAGCCTGAT | 176 | 0.3269127180192061 | No Hit |
| TCCTACGGGCGGCAGCAGTGGGGAATATTGGACAATGGGCGCAAGCCTGA | 169 | 0.31391050764344225 | No Hit |
| CCTACGGGAGGCAGCAGTGGGGAATATTGGACAATGGGCGAAAGCCTGAT | 164 | 0.30462321451789665 | No Hit |
| GACCTACGGGTGGCAGCAGTGGGGAATATTGGACAATGGGCGCAAGCCTG | 163 | 0.3027657558927875 | No Hit |
| TCCTACGGGTGGCAGCAGTGGGGAATATTGGACAATGGGCGAAAGCCTGA | 163 | 0.3027657558927875 | No Hit |
| CCCTACGGGAGGCAGCAGTGGGGAATATTGGACAATGGGCGCAAGCCTGA | 161 | 0.29905083864256926 | No Hit |
| TCCCTACGGGTGGCAGCAGTGGGGAATATTGGACAATGGGCGCAAGCCTG | 158 | 0.2934784627672419 | No Hit |
| TTCCTACGGGTGGCTGCAGTGGGGAATATTGGACAATGGGCGCAAGCCTG | 158 | 0.2934784627672419 | No Hit |
| CCCTACGGGTGGCTGCAGTGGGGAATATTGGACAATGGGCGCAAGCCTGA | 142 | 0.26375912476549584 | No Hit |
| AGCCTACGGGTGGCAGCAGTGGGGAATATTGGACAATGGGCGCAAGCCTG | 141 | 0.2619016661403867 | No Hit |
| ACCTACGGGCGGCAGCAGTGGGGAATATTGGACAATGGGCGCAAGCCTGA | 141 | 0.2619016661403867 | No Hit |
| TACCTACGGGGGGCAGCAGTGGGGAATATTGGACAATGGGCGCAAGCCTG | 139 | 0.25818674889016846 | No Hit |
| TACCTACGGGAGGCAGCAGTGGGGAATATTGGACAATGGGCGCAAGCCTG | 134 | 0.24889945576462283 | No Hit |
| ACCCTACGGGTGGCAGCAGTGGGGAATATTGGACAATGGGCGCAAGCCTG | 132 | 0.24518453851440458 | No Hit |
| CACCTACGGGTGGCAGCAGTGGGGAATATTGGACAATGGGCGCAAGCCTG | 131 | 0.24332707988929547 | No Hit |
| ACCTACGGGGGGCTGCAGTGGGGAATATTGGACAATGGGCGCAAGCCTGA | 129 | 0.23961216263907723 | No Hit |
| CCTACGGGAGGCTGCAGTGGGGAATATTGGACAATGGGCGAAAGCCTGAT | 125 | 0.2321823281386407 | No Hit |
| TTCCTACGGGGGGCAGCAGTGGGGAATATTGGACAATGGGCGCAAGCCTG | 123 | 0.22846741088842246 | No Hit |
| GGCCTACGGGTGGCAGCAGTGGGGAATATTGGACAATGGGCGCAAGCCTG | 122 | 0.22660995226331335 | No Hit |
| TACCTACGGGTGGCTGCAGTGGGGAATATTGGACAATGGGCGCAAGCCTG | 122 | 0.22660995226331335 | No Hit |
| TTCCTACGGGAGGCTGCAGTGGGGAATATTGGACAATGGGCGCAAGCCTG | 118 | 0.21918011776287682 | No Hit |
| GCCTACGGGCGGCAGCAGTGGGGAATATTGGACAATGGGCGCAAGCCTGA | 116 | 0.21546520051265858 | No Hit |
| TTTCCTACGGGTGGCAGCAGTGGGGAATATTGGACAATGGGCGCAAGCCT | 115 | 0.21360774188754947 | No Hit |
| CCCTACGGGGGGCAGCAGTGGGGAATATTGGACAATGGGCGCAAGCCTGA | 114 | 0.21175028326244033 | No Hit |
| GCCCTACGGGTGGCAGCAGTGGGGAATATTGGACAATGGGCGCAAGCCTG | 111 | 0.20617790738711295 | No Hit |
| TACCTACGGGAGGCTGCAGTGGGGAATATTGGACAATGGGCGCAAGCCTG | 108 | 0.2006055315117856 | No Hit |
| ATCCTACGGGAGGCAGCAGTGGGGAATATTGGACAATGGGCGCAAGCCTG | 106 | 0.19689061426156731 | No Hit |
| ACCTACGGGTGGCAGCAGTGGGGAATATTGGACAATGGGCGAAAGCCTGA | 106 | 0.19689061426156731 | No Hit |
| TGCCTACGGGAGGCAGCAGTGGGGAATATTGGACAATGGGCGCAAGCCTG | 105 | 0.1950331556364582 | No Hit |
| GCCTACGGGTGGCAGCAGTGGGGAATATTGGACAATGGGCGAAAGCCTGA | 104 | 0.19317569701134907 | No Hit |
| TTCCTACGGGCGGCAGCAGTGGGGAATATTGGACAATGGGCGCAAGCCTG | 100 | 0.18574586251091257 | No Hit |
| GGCCTACGGGAGGCAGCAGTGGGGAATATTGGACAATGGGCGCAAGCCTG | 97 | 0.1801734866355852 | No Hit |
| CTCCTACGGGAGGCAGCAGTGGGGAATATTGGACAATGGGCGCAAGCCTG | 97 | 0.1801734866355852 | No Hit |
| AACCTACGGGTGGCTGCAGTGGGGAATATTGGACAATGGGCGCAAGCCTG | 95 | 0.17645856938536694 | No Hit |
| AGCCTACGGGAGGCAGCAGTGGGGAATATTGGACAATGGGCGCAAGCCTG | 94 | 0.1746011107602578 | No Hit |
| GTCCTACGGGAGGCAGCAGTGGGGAATATTGGACAATGGGCGCAAGCCTG | 94 | 0.1746011107602578 | No Hit |
| CCCTACGGGAGGCTGCAGTGGGGAATATTGGACAATGGGCGCAAGCCTGA | 94 | 0.1746011107602578 | No Hit |
| GCCTACGGGGGGCTGCAGTGGGGAATATTGGACAATGGGCGCAAGCCTGA | 93 | 0.1727436521351487 | No Hit |
| CCTACGGGTGGCTGCATTTGATACGTCTAGACTAGAGTGTGAGAGAGGGT | 93 | 0.1727436521351487 | No Hit |
| ATTCCTACGGGTGGCAGCAGTGGGGAATATTGGACAATGGGCGCAAGCCT | 90 | 0.1671712762598213 | No Hit |
| ATCCTACGGGTGGCTGCAGTGGGGAATATTGGACAATGGGCGCAAGCCTG | 89 | 0.16531381763471217 | No Hit |
| CCTACGGGTGGCAGCAGTGGGGAATCTTGGACAATGGGCGAAAGCCCGAT | 89 | 0.16531381763471217 | No Hit |
| GACCTACGGGAGGCAGCAGTGGGGAATATTGGACAATGGGCGCAAGCCTG | 87 | 0.16159890038449393 | No Hit |
| CTCCTACGGGTGGCTGCAGTGGGGAATATTGGACAATGGGCGCAAGCCTG | 87 | 0.16159890038449393 | No Hit |
| AACCTACGGGAGGCAGCAGTGGGGAATATTGGACAATGGGCGCAAGCCTG | 85 | 0.15788398313427568 | No Hit |
| CCTACGGGCGGCAGCAGTGGGGAATATTGGACAATGGGCGAAAGCCTGAT | 84 | 0.15602652450916657 | No Hit |
| TGCCTACGGGGGGCAGCAGTGGGGAATATTGGACAATGGGCGCAAGCCTG | 84 | 0.15602652450916657 | No Hit |
| CACCTACGGGTGGCTGCAGTGGGGAATATTGGACAATGGGCGCAAGCCTG | 84 | 0.15602652450916657 | No Hit |
| TGCCTACGGGTGGCTGCAGTGGGGAATATTGGACAATGGGCGCAAGCCTG | 82 | 0.15231160725894832 | No Hit |
| TTACCTACGGGTGGCAGCAGTGGGGAATATTGGACAATGGGCGCAAGCCT | 79 | 0.14673923138362094 | No Hit |
| TCCTACGGGTGGCTGCAGTGGGGAATATTGGACAATGGGCGAAAGCCTGA | 79 | 0.14673923138362094 | No Hit |
| ACCCTACGGGAGGCAGCAGTGGGGAATATTGGACAATGGGCGCAAGCCTG | 77 | 0.1430243141334027 | No Hit |
| GTCCTACGGGTGGCTGCAGTGGGGAATATTGGACAATGGGCGCAAGCCTG | 77 | 0.1430243141334027 | No Hit |
| GACCTACGGGGGGCAGCAGTGGGGAATATTGGACAATGGGCGCAAGCCTG | 77 | 0.1430243141334027 | No Hit |
| AGCCTACGGGGGGCAGCAGTGGGGAATATTGGACAATGGGCGCAAGCCTG | 77 | 0.1430243141334027 | No Hit |
| TTCCCTACGGGTGGCAGCAGTGGGGAATATTGGACAATGGGCGCAAGCCT | 76 | 0.14116685550829355 | No Hit |
| ATCCTACGGGGGGCAGCAGTGGGGAATATTGGACAATGGGCGCAAGCCTG | 75 | 0.13930939688318444 | No Hit |
| TATCCTACGGGTGGCAGCAGTGGGGAATATTGGACAATGGGCGCAAGCCT | 74 | 0.13745193825807528 | No Hit |
| GTCCTACGGGGGGCAGCAGTGGGGAATATTGGACAATGGGCGCAAGCCTG | 73 | 0.13559447963296617 | No Hit |
| CACCTACGGGAGGCAGCAGTGGGGAATATTGGACAATGGGCGCAAGCCTG | 73 | 0.13559447963296617 | No Hit |
| TCCTAAGGGTGGCAGCAGTGGGGAATATTGGACAATGGGCGCAAGCCTGA | 71 | 0.13187956238274792 | No Hit |
| CCCCTACGGGTGGCAGCAGTGGGGAATATTGGACAATGGGCGCAAGCCTG | 70 | 0.1300221037576388 | No Hit |
| CCTACGGGGGGCAGCAGTGGGGAATCTTGGACAATGGGCGAAAGCCCGAT | 70 | 0.1300221037576388 | No Hit |
| ACCCTACGGGTGGCTGCAGTGGGGAATATTGGACAATGGGCGCAAGCCTG | 70 | 0.1300221037576388 | No Hit |
| AGCCTACGGGTGGCTGCAGTGGGGAATATTGGACAATGGGCGCAAGCCTG | 69 | 0.12816464513252968 | No Hit |
| TTCCTACGGGTGGCAGCAGTGGGGAATATTGGACAATGGGCGAAAGCCTG | 68 | 0.12630718650742057 | No Hit |
| AACCTACGGGGGGCAGCAGTGGGGAATATTGGACAATGGGCGCAAGCCTG | 68 | 0.12630718650742057 | No Hit |
| TGCCTACGGGAGGCTGCAGTGGGGAATATTGGACAATGGGCGCAAGCCTG | 68 | 0.12630718650742057 | No Hit |
| CCTACGGGGGGCTGCAGTGGGGAATATTGGACAATGGGCGAAAGCCTGAT | 68 | 0.12630718650742057 | No Hit |
| TCCTACGGGAGGCAGCAGTGGGGAATATTGGACAATGGGCGAAAGCCTGA | 68 | 0.12630718650742057 | No Hit |
| TACCTACGGGCGGCAGCAGTGGGGAATATTGGACAATGGGCGCAAGCCTG | 67 | 0.12444972788231141 | No Hit |
| CGCCTACGGGTGGCAGCAGTGGGGAATATTGGACAATGGGCGCAAGCCTG | 66 | 0.12259226925720229 | No Hit |
| AATCCTACGGGTGGCAGCAGTGGGGAATATTGGACAATGGGCGCAAGCCT | 66 | 0.12259226925720229 | No Hit |
| ATCCTACGGGAGGCTGCAGTGGGGAATATTGGACAATGGGCGCAAGCCTG | 64 | 0.11887735200698404 | No Hit |
| TCCCTACGGGTGGCTGCAGTGGGGAATATTGGACAATGGGCGCAAGCCTG | 64 | 0.11887735200698404 | No Hit |
| CCTACGGGGGGCTGCATTTGATACGTCTAGACTAGAGTGTGAGAGAGGGT | 64 | 0.11887735200698404 | No Hit |
| GCCCTACGGGAGGCAGCAGTGGGGAATATTGGACAATGGGCGCAAGCCTG | 64 | 0.11887735200698404 | No Hit |
| ACCTACGGGTGGCTGCAGTGGGGAATATTGGACAATGGGCGAAAGCCTGA | 63 | 0.11701989338187492 | No Hit |
| TAACCTACGGGTGGCAGCAGTGGGGAATATTGGACAATGGGCGCAAGCCT | 63 | 0.11701989338187492 | No Hit |
| AACCTACGGGAGGCTGCAGTGGGGAATATTGGACAATGGGCGCAAGCCTG | 62 | 0.1151624347567658 | No Hit |
| GACCTACGGGTGGCTGCAGTGGGGAATATTGGACAATGGGCGCAAGCCTG | 62 | 0.1151624347567658 | No Hit |
| TCCTACGGGCGGCTGCAGTGGGGAATATTGGACAATGGGCGCAAGCCTGA | 62 | 0.1151624347567658 | No Hit |
| ACCTACGGGAGGCAGCAGTGGGGAATATTGGACAATGGGCGAAAGCCTGA | 61 | 0.11330497613165667 | No Hit |
| TGCCTACGGGCGGCAGCAGTGGGGAATATTGGACAATGGGCGCAAGCCTG | 60 | 0.11144751750654754 | No Hit |
| GTCCTACGGGAGGCTGCAGTGGGGAATATTGGACAATGGGCGCAAGCCTG | 60 | 0.11144751750654754 | No Hit |
| CTCCTACGGGGGGCAGCAGTGGGGAATATTGGACAATGGGCGCAAGCCTG | 60 | 0.11144751750654754 | No Hit |
| CCCTACGGGTGGCAGCAGTGGGGAATATTGGACAATGGGCGAAAGCCTGA | 58 | 0.10773260025632929 | No Hit |
| TTCCTACGGGGGGCTGCAGTGGGGAATATTGGACAATGGGCGCAAGCCTG | 57 | 0.10587514163122017 | No Hit |
| ATCCTACGGGCGGCAGCAGTGGGGAATATTGGACAATGGGCGCAAGCCTG | 56 | 0.10401768300611104 | No Hit |
| ACCTACGGGTGGCAGCAGTGGGGAATCTTGGACAATGGGCGAAAGCCCGA | 55 | 0.10216022438100192 | No Hit |
| CACTACGGGTGGCAGCAGTGGGGAATATTGGACAATGGGCGCAAGCCTGA | 55 | 0.10216022438100192 | No Hit |
| TCCTACGGGGGGCAGCAGTGGGGAATATTGGACAATGGGCGAAAGCCTGA | 55 | 0.10216022438100192 | No Hit |
| CCCTACGGGCGGCAGCAGTGGGGAATATTGGACAATGGGCGCAAGCCTGA | 55 | 0.10216022438100192 | No Hit |
| GTTCCTACGGGTGGCAGCAGTGGGGAATATTGGACAATGGGCGCAAGCCT | 54 | 0.1003027657558928 | No Hit |

## Adapter Content

Produced by FastQC (version 0.12.1)
