# Supplementary material for: Bacterial community data associated with Stephanitis pyri using 16S rRNA gene metabarcoding
Source: Data Brief. 2026 Jul 29;68:113107. doi: 10.1016/j.dib.2026.113107 (PMC13474393; doi:10.1016/j.dib.2026.113107)
Supplement: Supplementary file 1 [file mmc1.zip › supplementary_material_1/fastqc/c4-N4062-16Sv3v4_S52_L001_R2_001_fastqc.zip!/c4-N4062-16Sv3v4_S52_L001_R2_001_fastqc/fastqc_report.html]

c4-N4062-16Sv3v4\_S52\_L001\_R2\_001.fastq.gz FastQC Report 

FastQC Report

Mon 13 Oct 2025  
c4-N4062-16Sv3v4\_S52\_L001\_R2\_001.fastq.gz

## Summary

- Basic Statistics
- Per base sequence quality
- Per tile sequence quality
- Per sequence quality scores
- Per base sequence content
- Per sequence GC content
- Per base N content
- Sequence Length Distribution
- Sequence Duplication Levels
- Overrepresented sequences
- Adapter Content

## Basic Statistics

| Measure | Value |
| --- | --- |
| Filename | c4-N4062-16Sv3v4\_S52\_L001\_R2\_001.fastq.gz |
| File type | Conventional base calls |
| Encoding | Sanger / Illumina 1.9 |
| Total Sequences | 53837 |
| Total Bases | 16.2 Mbp |
| Sequences flagged as poor quality | 0 |
| Sequence length | 301 |
| %GC | 54 |

## Per base sequence quality

## Per tile sequence quality

## Per sequence quality scores

## Per base sequence content

## Per sequence GC content

## Per base N content

## Sequence Length Distribution

## Sequence Duplication Levels

## Overrepresented sequences

| Sequence | Count | Percentage | Possible Source |
| --- | --- | --- | --- |
| GACTACTAGGGTATCTAATCCTGTTTGCTCCCCACGCTTTCGCGCCTCAG | 1238 | 2.2995337778850975 | No Hit |
| GACTACTCGGGTATCTAATCCTGTTTGCTCCCCACGCTTTCGCGCCTCAG | 1074 | 1.9949105633672009 | No Hit |
| GACTACTGGGGTATCTAATCCTGTTTGCTCCCCACGCTTTCGCGCCTCAG | 1015 | 1.8853205044857626 | No Hit |
| GACTACAAGGGTATCTAATCCTGTTTGCTCCCCACGCTTTCGCGCCTCAG | 913 | 1.6958597247246316 | No Hit |
| GACTACCAGGGTATCTAATCCTGTTTGCTCCCCACGCTTTCGCGCCTCAG | 781 | 1.4506751862102272 | No Hit |
| GACTACACGGGTATCTAATCCTGTTTGCTCCCCACGCTTTCGCGCCTCAG | 709 | 1.3169381652023702 | No Hit |
| GACTACAGGGGTATCTAATCCTGTTTGCTCCCCACGCTTTCGCGCCTCAG | 689 | 1.2797889927001875 | No Hit |
| GACTACCGGGGTATCTAATCCTGTTTGCTCCCCACGCTTTCGCGCCTCAG | 585 | 1.0866132956888386 | No Hit |
| GACTACCCGGGTATCTAATCCTGTTTGCTCCCCACGCTTTCGCGCCTCAG | 492 | 0.9138696435536898 | No Hit |
| TGACTACTAGGGTATCTAATCCTGTTTGCTCCCCACGCTTTCGCGCCTCA | 427 | 0.7931348329215967 | No Hit |
| TGACTACTCGGGTATCTAATCCTGTTTGCTCCCCACGCTTTCGCGCCTCA | 369 | 0.6854022326652675 | No Hit |
| TGACTACAAGGGTATCTAATCCTGTTTGCTCCCCACGCTTTCGCGCCTCA | 303 | 0.5628099634080651 | No Hit |
| AGACTACTAGGGTATCTAATCCTGTTTGCTCCCCACGCTTTCGCGCCTCA | 296 | 0.5498077530323011 | No Hit |
| TGACTACTGGGGTATCTAATCCTGTTTGCTCCCCACGCTTTCGCGCCTCA | 285 | 0.5293757081561008 | No Hit |
| GGACTACTAGGGTATCTAATCCTGTTTGCTCCCCACGCTTTCGCGCCTCA | 277 | 0.5145160391552278 | No Hit |
| CGACTACTAGGGTATCTAATCCTGTTTGCTCCCCACGCTTTCGCGCCTCA | 272 | 0.5052287460296823 | No Hit |
| TGACTACCAGGGTATCTAATCCTGTTTGCTCCCCACGCTTTCGCGCCTCA | 271 | 0.503371287404573 | No Hit |
| TGACTACAGGGGTATCTAATCCTGTTTGCTCCCCACGCTTTCGCGCCTCA | 260 | 0.48293924252837267 | No Hit |
| GGACTACTGGGGTATCTAATCCTGTTTGCTCCCCACGCTTTCGCGCCTCA | 244 | 0.4532199045266267 | No Hit |
| AGACTACTGGGGTATCTAATCCTGTTTGCTCCCCACGCTTTCGCGCCTCA | 240 | 0.44579007002619014 | No Hit |
| TGACTACACGGGTATCTAATCCTGTTTGCTCCCCACGCTTTCGCGCCTCA | 228 | 0.42350056652488066 | No Hit |
| AGACTACTCGGGTATCTAATCCTGTTTGCTCCCCACGCTTTCGCGCCTCA | 226 | 0.4197856492746624 | No Hit |
| GACTACTAGGGTATCTAATCCTGTTTGCTCCCCACGCTTTCGCACCTCAG | 218 | 0.4049259802737894 | No Hit |
| GGACTACTCGGGTATCTAATCCTGTTTGCTCCCCACGCTTTCGCGCCTCA | 217 | 0.40306852164868023 | No Hit |
| GGACTACAAGGGTATCTAATCCTGTTTGCTCCCCACGCTTTCGCGCCTCA | 213 | 0.39563868714824374 | No Hit |
| CGACTACTGGGGTATCTAATCCTGTTTGCTCCCCACGCTTTCGCGCCTCA | 208 | 0.38635139402269814 | No Hit |
| CGACTACAAGGGTATCTAATCCTGTTTGCTCCCCACGCTTTCGCGCCTCA | 199 | 0.369634266396716 | No Hit |
| GACTACTCGGGTATCTAATCCTGTTTGCTCCCCACGCTTTCGCACCTCAG | 197 | 0.36591934914649776 | No Hit |
| CGACTACTCGGGTATCTAATCCTGTTTGCTCCCCACGCTTTCGCGCCTCA | 189 | 0.3510596801456248 | No Hit |
| AGACTACAAGGGTATCTAATCCTGTTTGCTCCCCACGCTTTCGCGCCTCA | 187 | 0.3473447628954065 | No Hit |
| GACTACTGGGGTATCTAATCCTGTTTGCTCCCCACGCTTTCGCACCTCAG | 177 | 0.32877017664431524 | No Hit |
| GGACTACCAGGGTATCTAATCCTGTTTGCTCCCCACGCTTTCGCGCCTCA | 176 | 0.3269127180192061 | No Hit |
| CGACTACCAGGGTATCTAATCCTGTTTGCTCCCCACGCTTTCGCGCCTCA | 173 | 0.32134034214387874 | No Hit |
| TGACTACCCGGGTATCTAATCCTGTTTGCTCCCCACGCTTTCGCGCCTCA | 173 | 0.32134034214387874 | No Hit |
| AGACTACCAGGGTATCTAATCCTGTTTGCTCCCCACGCTTTCGCGCCTCA | 170 | 0.31576796626855136 | No Hit |
| AGACTACAGGGGTATCTAATCCTGTTTGCTCCCCACGCTTTCGCGCCTCA | 168 | 0.31205304901833314 | No Hit |
| TGACTACCGGGGTATCTAATCCTGTTTGCTCCCCACGCTTTCGCGCCTCA | 164 | 0.30462321451789665 | No Hit |
| GGACTACACGGGTATCTAATCCTGTTTGCTCCCCACGCTTTCGCGCCTCA | 163 | 0.3027657558927875 | No Hit |
| GGACTACAGGGGTATCTAATCCTGTTTGCTCCCCACGCTTTCGCGCCTCA | 163 | 0.3027657558927875 | No Hit |
| AGACTACACGGGTATCTAATCCTGTTTGCTCCCCACGCTTTCGCGCCTCA | 156 | 0.2897635455170236 | No Hit |
| GACTACAAGGGTATCTAATCCTGTTTGCTCCCCACGCTTTCGCACCTCAG | 152 | 0.2823337110165871 | No Hit |
| AGACTACCCGGGTATCTAATCCTGTTTGCTCCCCACGCTTTCGCGCCTCA | 150 | 0.2786187937663689 | No Hit |
| TTGACTACTCGGGTATCTAATCCTGTTTGCTCCCCACGCTTTCGCGCCTC | 143 | 0.265616583390605 | No Hit |
| GACTACAGGGGTATCTAATCCTGTTTGCTCCCCACGCTTTCGCACCTCAG | 138 | 0.25632929026505935 | No Hit |
| TTGACTACTAGGGTATCTAATCCTGTTTGCTCCCCACGCTTTCGCGCCTC | 136 | 0.25261437301484113 | No Hit |
| AGACTACCGGGGTATCTAATCCTGTTTGCTCCCCACGCTTTCGCGCCTCA | 135 | 0.25075691438973197 | No Hit |
| GACTACACGGGTATCTAATCCTGTTTGCTCCCCACGCTTTCGCACCTCAG | 133 | 0.24704199713951372 | No Hit |
| CGACTACAGGGGTATCTAATCCTGTTTGCTCCCCACGCTTTCGCGCCTCA | 130 | 0.24146962126418633 | No Hit |
| TTGACTACTGGGGTATCTAATCCTGTTTGCTCCCCACGCTTTCGCGCCTC | 125 | 0.2321823281386407 | No Hit |
| GACTACCAGGGTATCTAATCCTGTTTGCTCCCCACGCTTTCGCACCTCAG | 124 | 0.2303248695135316 | No Hit |
| CGACTACACGGGTATCTAATCCTGTTTGCTCCCCACGCTTTCGCGCCTCA | 122 | 0.22660995226331335 | No Hit |
| GGACTACCGGGGTATCTAATCCTGTTTGCTCCCCACGCTTTCGCGCCTCA | 120 | 0.22289503501309507 | No Hit |
| CGACTACCCGGGTATCTAATCCTGTTTGCTCCCCACGCTTTCGCGCCTCA | 112 | 0.20803536601222208 | No Hit |
| ATGACTACTAGGGTATCTAATCCTGTTTGCTCCCCACGCTTTCGCGCCTC | 97 | 0.1801734866355852 | No Hit |
| TTGACTACAGGGGTATCTAATCCTGTTTGCTCCCCACGCTTTCGCGCCTC | 97 | 0.1801734866355852 | No Hit |
| GACTACCCGGGTATCTAATCCTGTTTGCTCCCCACGCTTTCGCACCTCAG | 96 | 0.17831602801047605 | No Hit |
| TGGACTACTAGGGTATCTAATCCTGTTTGCTCCCCACGCTTTCGCGCCTC | 94 | 0.1746011107602578 | No Hit |
| GACTACCGGGGTATCTAATCCTGTTTGCTCCCCACGCTTTCGCACCTCAG | 94 | 0.1746011107602578 | No Hit |
| GGGGGGGGGGGGGGGGGGGGGGGGGGGGGGGGGGGGGGGGGGGGGGGGGG | 91 | 0.16902873488493045 | No Hit |
| TAGACTACTCGGGTATCTAATCCTGTTTGCTCCCCACGCTTTCGCGCCTC | 89 | 0.16531381763471217 | No Hit |
| GTGACTACTCGGGTATCTAATCCTGTTTGCTCCCCACGCTTTCGCGCCTC | 86 | 0.15974144175938482 | No Hit |
| GTGACTACTAGGGTATCTAATCCTGTTTGCTCCCCACGCTTTCGCGCCTC | 86 | 0.15974144175938482 | No Hit |
| TAGACTACTGGGGTATCTAATCCTGTTTGCTCCCCACGCTTTCGCGCCTC | 82 | 0.15231160725894832 | No Hit |
| ATGACTACAAGGGTATCTAATCCTGTTTGCTCCCCACGCTTTCGCGCCTC | 81 | 0.15045414863383919 | No Hit |
| TCGACTACTAGGGTATCTAATCCTGTTTGCTCCCCACGCTTTCGCGCCTC | 80 | 0.14859669000873005 | No Hit |
| CTGACTACTAGGGTATCTAATCCTGTTTGCTCCCCACGCTTTCGCGCCTC | 80 | 0.14859669000873005 | No Hit |
| GTGACTACTGGGGTATCTAATCCTGTTTGCTCCCCACGCTTTCGCGCCTC | 80 | 0.14859669000873005 | No Hit |
| ATGACTACTGGGGTATCTAATCCTGTTTGCTCCCCACGCTTTCGCGCCTC | 80 | 0.14859669000873005 | No Hit |
| TTGACTACCAGGGTATCTAATCCTGTTTGCTCCCCACGCTTTCGCGCCTC | 79 | 0.14673923138362094 | No Hit |
| TCGACTACTGGGGTATCTAATCCTGTTTGCTCCCCACGCTTTCGCGCCTC | 78 | 0.1448817727585118 | No Hit |
| CTGACTACTCGGGTATCTAATCCTGTTTGCTCCCCACGCTTTCGCGCCTC | 77 | 0.1430243141334027 | No Hit |
| ATGACTACTCGGGTATCTAATCCTGTTTGCTCCCCACGCTTTCGCGCCTC | 77 | 0.1430243141334027 | No Hit |
| TAGACTACTAGGGTATCTAATCCTGTTTGCTCCCCACGCTTTCGCGCCTC | 77 | 0.1430243141334027 | No Hit |
| CGACTACCGGGGTATCTAATCCTGTTTGCTCCCCACGCTTTCGCGCCTCA | 76 | 0.14116685550829355 | No Hit |
| TGGACTACTCGGGTATCTAATCCTGTTTGCTCCCCACGCTTTCGCGCCTC | 74 | 0.13745193825807528 | No Hit |
| TGGACTACAAGGGTATCTAATCCTGTTTGCTCCCCACGCTTTCGCGCCTC | 73 | 0.13559447963296617 | No Hit |
| CTGACTACTGGGGTATCTAATCCTGTTTGCTCCCCACGCTTTCGCGCCTC | 72 | 0.13373702100785706 | No Hit |
| AAGACTACTCGGGTATCTAATCCTGTTTGCTCCCCACGCTTTCGCGCCTC | 72 | 0.13373702100785706 | No Hit |
| TAGACTACAAGGGTATCTAATCCTGTTTGCTCCCCACGCTTTCGCGCCTC | 72 | 0.13373702100785706 | No Hit |
| CTGACTACAAGGGTATCTAATCCTGTTTGCTCCCCACGCTTTCGCGCCTC | 71 | 0.13187956238274792 | No Hit |
| ATGACTACACGGGTATCTAATCCTGTTTGCTCCCCACGCTTTCGCGCCTC | 70 | 0.1300221037576388 | No Hit |
| TGACTACTAGGGTATCTAATCCTGTTTGCTCCCCACGCTTTCGCACCTCA | 69 | 0.12816464513252968 | No Hit |
| TTGACTACAAGGGTATCTAATCCTGTTTGCTCCCCACGCTTTCGCGCCTC | 66 | 0.12259226925720229 | No Hit |
| TAGACTACAGGGGTATCTAATCCTGTTTGCTCCCCACGCTTTCGCGCCTC | 65 | 0.12073481063209317 | No Hit |
| TTGACTACACGGGTATCTAATCCTGTTTGCTCCCCACGCTTTCGCGCCTC | 65 | 0.12073481063209317 | No Hit |
| GACTACTAGGGTATCTAATCCTGTTTGCTCCCCACGCTTTCGCGCCTAAG | 64 | 0.11887735200698404 | No Hit |
| TGGACTACTGGGGTATCTAATCCTGTTTGCTCCCCACGCTTTCGCGCCTC | 64 | 0.11887735200698404 | No Hit |
| TAGACTACACGGGTATCTAATCCTGTTTGCTCCCCACGCTTTCGCGCCTC | 64 | 0.11887735200698404 | No Hit |
| TTGACTACCCGGGTATCTAATCCTGTTTGCTCCCCACGCTTTCGCGCCTC | 63 | 0.11701989338187492 | No Hit |
| TCGACTACAAGGGTATCTAATCCTGTTTGCTCCCCACGCTTTCGCGCCTC | 63 | 0.11701989338187492 | No Hit |
| CGGACTACTGGGGTATCTAATCCTGTTTGCTCCCCACGCTTTCGCGCCTC | 63 | 0.11701989338187492 | No Hit |
| CGGACTACTAGGGTATCTAATCCTGTTTGCTCCCCACGCTTTCGCGCCTC | 62 | 0.1151624347567658 | No Hit |
| AGGACTACTAGGGTATCTAATCCTGTTTGCTCCCCACGCTTTCGCGCCTC | 62 | 0.1151624347567658 | No Hit |
| TGACTACTCGGGTATCTAATCCTGTTTGCTCCCCACGCTTTCGCACCTCA | 62 | 0.1151624347567658 | No Hit |
| CAGACTACTCGGGTATCTAATCCTGTTTGCTCCCCACGCTTTCGCGCCTC | 60 | 0.11144751750654754 | No Hit |
| GGACTACCCGGGTATCTAATCCTGTTTGCTCCCCACGCTTTCGCGCCTCA | 60 | 0.11144751750654754 | No Hit |
| GACTACTCGGGTATCTAATCCTGTTTGCTCCCCACGCTTTCGCGCCTAAG | 59 | 0.10959005888143841 | No Hit |
| AGACTACTAGGGTATCTAATCCTGTTTGCTCCCCACGCTTTCGCACCTCA | 59 | 0.10959005888143841 | No Hit |
| GACTACTGGGGTATCTAATCCTGTTTGCTCCCCACGCTTTCGCGCCTAAG | 59 | 0.10959005888143841 | No Hit |
| ATGACTACCAGGGTATCTAATCCTGTTTGCTCCCCACGCTTTCGCGCCTC | 59 | 0.10959005888143841 | No Hit |
| GGGACTACTGGGGTATCTAATCCTGTTTGCTCCCCACGCTTTCGCGCCTC | 58 | 0.10773260025632929 | No Hit |
| TCGACTACTCGGGTATCTAATCCTGTTTGCTCCCCACGCTTTCGCGCCTC | 58 | 0.10773260025632929 | No Hit |
| TTTGACTACTAGGGTATCTAATCCTGTTTGCTCCCCACGCTTTCGCGCCT | 58 | 0.10773260025632929 | No Hit |
| TGACTACAAGGGTATCTAATCCTGTTTGCTCCCCACGCTTTCGCACCTCA | 57 | 0.10587514163122017 | No Hit |
| GTGACTACAGGGGTATCTAATCCTGTTTGCTCCCCACGCTTTCGCGCCTC | 57 | 0.10587514163122017 | No Hit |
| GTGACTACAAGGGTATCTAATCCTGTTTGCTCCCCACGCTTTCGCGCCTC | 56 | 0.10401768300611104 | No Hit |
| TAGACTACCAGGGTATCTAATCCTGTTTGCTCCCCACGCTTTCGCGCCTC | 56 | 0.10401768300611104 | No Hit |
| GGGACTACTAGGGTATCTAATCCTGTTTGCTCCCCACGCTTTCGCGCCTC | 56 | 0.10401768300611104 | No Hit |
| GTGACTACCCGGGTATCTAATCCTGTTTGCTCCCCACGCTTTCGCGCCTC | 56 | 0.10401768300611104 | No Hit |
| GAGACTACTGGGGTATCTAATCCTGTTTGCTCCCCACGCTTTCGCGCCTC | 55 | 0.10216022438100192 | No Hit |
| TCGACTACACGGGTATCTAATCCTGTTTGCTCCCCACGCTTTCGCGCCTC | 55 | 0.10216022438100192 | No Hit |
| TCGACTACCAGGGTATCTAATCCTGTTTGCTCCCCACGCTTTCGCGCCTC | 55 | 0.10216022438100192 | No Hit |
| TTGACTACCGGGGTATCTAATCCTGTTTGCTCCCCACGCTTTCGCGCCTC | 54 | 0.1003027657558928 | No Hit |
| GACTACTAGGGTATCTAATCCCGTTCGCTCCCCATGCTTTCGCACTCCAG | 54 | 0.1003027657558928 | No Hit |

## Adapter Content

Produced by FastQC (version 0.12.1)
