# Supplementary material for: Bacterial community data associated with Stephanitis pyri using 16S rRNA gene metabarcoding
Source: Data Brief. 2026 Jul 29;68:113107. doi: 10.1016/j.dib.2026.113107 (PMC13474393; doi:10.1016/j.dib.2026.113107)
Supplement: Supplementary file 1 [file mmc1.zip › supplementary_material_1/fastqc/Extr-N4063-16Sv3v4_S53_L001_R1_001_fastqc.zip!/Extr-N4063-16Sv3v4_S53_L001_R1_001_fastqc/fastqc_report.html]

Extr-N4063-16Sv3v4\_S53\_L001\_R1\_001.fastq.gz FastQC Report 

FastQC Report

Mon 13 Oct 2025  
Extr-N4063-16Sv3v4\_S53\_L001\_R1\_001.fastq.gz

## Summary

- Basic Statistics
- Per base sequence quality
- Per tile sequence quality
- Per sequence quality scores
- Per base sequence content
- Per sequence GC content
- Per base N content
- Sequence Length Distribution
- Sequence Duplication Levels
- Overrepresented sequences
- Adapter Content

## Basic Statistics

| Measure | Value |
| --- | --- |
| Filename | Extr-N4063-16Sv3v4\_S53\_L001\_R1\_001.fastq.gz |
| File type | Conventional base calls |
| Encoding | Sanger / Illumina 1.9 |
| Total Sequences | 34560 |
| Total Bases | 10.4 Mbp |
| Sequences flagged as poor quality | 0 |
| Sequence length | 301 |
| %GC | 53 |

## Per base sequence quality

## Per tile sequence quality

## Per sequence quality scores

## Per base sequence content

## Per sequence GC content

## Per base N content

## Sequence Length Distribution

## Sequence Duplication Levels

## Overrepresented sequences

| Sequence | Count | Percentage | Possible Source |
| --- | --- | --- | --- |
| CCTACGGGTGGCAGCAGTGGGGAATATTGGACAATGGGCGAAAGCCTGAT | 1752 | 5.069444444444445 | No Hit |
| CCTACGGGAGGCAGCAGTGGGGAATATTGGACAATGGGCGAAAGCCTGAT | 834 | 2.4131944444444446 | No Hit |
| TCCTACGGGTGGCAGCAGTGGGGAATATTGGACAATGGGCGAAAGCCTGA | 664 | 1.9212962962962963 | No Hit |
| ACCTACGGGTGGCAGCAGTGGGGAATATTGGACAATGGGCGAAAGCCTGA | 645 | 1.8663194444444444 | No Hit |
| CCTACGGGTGGCTGCAGTGGGGAATATTGGACAATGGGCGAAAGCCTGAT | 637 | 1.8431712962962963 | No Hit |
| CCTACGGGGGGCAGCAGTGGGGAATATTGGACAATGGGCGAAAGCCTGAT | 630 | 1.8229166666666667 | No Hit |
| GCCTACGGGTGGCAGCAGTGGGGAATATTGGACAATGGGCGAAAGCCTGA | 480 | 1.3888888888888888 | No Hit |
| CCTACGGGAGGCTGCAGTGGGGAATATTGGACAATGGGCGAAAGCCTGAT | 436 | 1.2615740740740742 | No Hit |
| TCCTACGGGAGGCAGCAGTGGGGAATATTGGACAATGGGCGAAAGCCTGA | 388 | 1.1226851851851853 | No Hit |
| CCTACGGGCGGCAGCAGTGGGGAATATTGGACAATGGGCGAAAGCCTGAT | 339 | 0.9809027777777778 | No Hit |
| ACCTACGGGAGGCAGCAGTGGGGAATATTGGACAATGGGCGAAAGCCTGA | 339 | 0.9809027777777778 | No Hit |
| GCCTACGGGAGGCAGCAGTGGGGAATATTGGACAATGGGCGAAAGCCTGA | 290 | 0.8391203703703705 | No Hit |
| TTCCTACGGGTGGCAGCAGTGGGGAATATTGGACAATGGGCGAAAGCCTG | 283 | 0.8188657407407407 | No Hit |
| TACCTACGGGTGGCAGCAGTGGGGAATATTGGACAATGGGCGAAAGCCTG | 278 | 0.8043981481481481 | No Hit |
| CCCTACGGGTGGCAGCAGTGGGGAATATTGGACAATGGGCGAAAGCCTGA | 256 | 0.7407407407407408 | No Hit |
| ACCTACGGGGGGCAGCAGTGGGGAATATTGGACAATGGGCGAAAGCCTGA | 251 | 0.7262731481481481 | No Hit |
| CCTACGGGGGGCTGCAGTGGGGAATATTGGACAATGGGCGAAAGCCTGAT | 245 | 0.7089120370370371 | No Hit |
| TCCTACGGGTGGCTGCAGTGGGGAATATTGGACAATGGGCGAAAGCCTGA | 242 | 0.7002314814814814 | No Hit |
| TCCTACGGGGGGCAGCAGTGGGGAATATTGGACAATGGGCGAAAGCCTGA | 237 | 0.6857638888888888 | No Hit |
| ACCTACGGGTGGCTGCAGTGGGGAATATTGGACAATGGGCGAAAGCCTGA | 208 | 0.6018518518518519 | No Hit |
| AACCTACGGGTGGCAGCAGTGGGGAATATTGGACAATGGGCGAAAGCCTG | 204 | 0.5902777777777778 | No Hit |
| GCCTACGGGGGGCAGCAGTGGGGAATATTGGACAATGGGCGAAAGCCTGA | 203 | 0.5873842592592593 | No Hit |
| TCCTACGGGAGGCTGCAGTGGGGAATATTGGACAATGGGCGAAAGCCTGA | 194 | 0.5613425925925927 | No Hit |
| ATCCTACGGGTGGCAGCAGTGGGGAATATTGGACAATGGGCGAAAGCCTG | 172 | 0.49768518518518523 | No Hit |
| TTCCTACGGGAGGCAGCAGTGGGGAATATTGGACAATGGGCGAAAGCCTG | 166 | 0.48032407407407407 | No Hit |
| ACCTACGGGAGGCTGCAGTGGGGAATATTGGACAATGGGCGAAAGCCTGA | 163 | 0.47164351851851855 | No Hit |
| GTCCTACGGGTGGCAGCAGTGGGGAATATTGGACAATGGGCGAAAGCCTG | 162 | 0.46875 | No Hit |
| TGCCTACGGGTGGCAGCAGTGGGGAATATTGGACAATGGGCGAAAGCCTG | 159 | 0.4600694444444445 | No Hit |
| GCCTACGGGTGGCTGCAGTGGGGAATATTGGACAATGGGCGAAAGCCTGA | 159 | 0.4600694444444445 | No Hit |
| TCCTACGGGCGGCAGCAGTGGGGAATATTGGACAATGGGCGAAAGCCTGA | 141 | 0.40798611111111116 | No Hit |
| AGCCTACGGGTGGCAGCAGTGGGGAATATTGGACAATGGGCGAAAGCCTG | 140 | 0.40509259259259256 | No Hit |
| CACCTACGGGTGGCAGCAGTGGGGAATATTGGACAATGGGCGAAAGCCTG | 134 | 0.38773148148148145 | No Hit |
| TCCCTACGGGTGGCAGCAGTGGGGAATATTGGACAATGGGCGAAAGCCTG | 134 | 0.38773148148148145 | No Hit |
| ACCTACGGGCGGCAGCAGTGGGGAATATTGGACAATGGGCGAAAGCCTGA | 127 | 0.36747685185185186 | No Hit |
| GCCTACGGGAGGCTGCAGTGGGGAATATTGGACAATGGGCGAAAGCCTGA | 126 | 0.3645833333333333 | No Hit |
| CCTACGGGCGGCTGCAGTGGGGAATATTGGACAATGGGCGAAAGCCTGAT | 126 | 0.3645833333333333 | No Hit |
| CTCCTACGGGTGGCAGCAGTGGGGAATATTGGACAATGGGCGAAAGCCTG | 126 | 0.3645833333333333 | No Hit |
| GACCTACGGGTGGCAGCAGTGGGGAATATTGGACAATGGGCGAAAGCCTG | 124 | 0.3587962962962963 | No Hit |
| TACCTACGGGAGGCAGCAGTGGGGAATATTGGACAATGGGCGAAAGCCTG | 123 | 0.3559027777777778 | No Hit |
| ACCCTACGGGTGGCAGCAGTGGGGAATATTGGACAATGGGCGAAAGCCTG | 120 | 0.3472222222222222 | No Hit |
| AACCTACGGGAGGCAGCAGTGGGGAATATTGGACAATGGGCGAAAGCCTG | 118 | 0.3414351851851852 | No Hit |
| CCCTACGGGAGGCAGCAGTGGGGAATATTGGACAATGGGCGAAAGCCTGA | 113 | 0.32696759259259256 | No Hit |
| CCCTACGGGTGGCTGCAGTGGGGAATATTGGACAATGGGCGAAAGCCTGA | 110 | 0.31828703703703703 | No Hit |
| TGCCTACGGGAGGCAGCAGTGGGGAATATTGGACAATGGGCGAAAGCCTG | 109 | 0.31539351851851855 | No Hit |
| GTCCTACGGGAGGCAGCAGTGGGGAATATTGGACAATGGGCGAAAGCCTG | 107 | 0.3096064814814815 | No Hit |
| TTCCTACGGGGGGCAGCAGTGGGGAATATTGGACAATGGGCGAAAGCCTG | 105 | 0.3038194444444445 | No Hit |
| CCCTACGGGGGGCAGCAGTGGGGAATATTGGACAATGGGCGAAAGCCTGA | 103 | 0.2980324074074074 | No Hit |
| TCCTACGGGGGGCTGCAGTGGGGAATATTGGACAATGGGCGAAAGCCTGA | 101 | 0.29224537037037035 | No Hit |
| TTCCTACGGGTGGCTGCAGTGGGGAATATTGGACAATGGGCGAAAGCCTG | 101 | 0.29224537037037035 | No Hit |
| TACCTACGGGTGGCTGCAGTGGGGAATATTGGACAATGGGCGAAAGCCTG | 98 | 0.2835648148148148 | No Hit |
| GCCCTACGGGTGGCAGCAGTGGGGAATATTGGACAATGGGCGAAAGCCTG | 97 | 0.28067129629629634 | No Hit |
| ATCCTACGGGAGGCAGCAGTGGGGAATATTGGACAATGGGCGAAAGCCTG | 91 | 0.2633101851851852 | No Hit |
| AGCCTACGGGAGGCAGCAGTGGGGAATATTGGACAATGGGCGAAAGCCTG | 89 | 0.25752314814814814 | No Hit |
| TTTCCTACGGGTGGCAGCAGTGGGGAATATTGGACAATGGGCGAAAGCCT | 86 | 0.24884259259259262 | No Hit |
| TACCTACGGGGGGCAGCAGTGGGGAATATTGGACAATGGGCGAAAGCCTG | 85 | 0.24594907407407407 | No Hit |
| TAACCTACGGGTGGCAGCAGTGGGGAATATTGGACAATGGGCGAAAGCCT | 84 | 0.24305555555555555 | No Hit |
| TTACCTACGGGTGGCAGCAGTGGGGAATATTGGACAATGGGCGAAAGCCT | 84 | 0.24305555555555555 | No Hit |
| GCCTACGGGCGGCAGCAGTGGGGAATATTGGACAATGGGCGAAAGCCTGA | 83 | 0.24016203703703703 | No Hit |
| GACCTACGGGAGGCAGCAGTGGGGAATATTGGACAATGGGCGAAAGCCTG | 79 | 0.22858796296296294 | No Hit |
| TACCTACGGGAGGCTGCAGTGGGGAATATTGGACAATGGGCGAAAGCCTG | 78 | 0.22569444444444442 | No Hit |
| ATTCCTACGGGTGGCAGCAGTGGGGAATATTGGACAATGGGCGAAAGCCT | 77 | 0.22280092592592593 | No Hit |
| ACCTACGGGGGGCTGCAGTGGGGAATATTGGACAATGGGCGAAAGCCTGA | 76 | 0.2199074074074074 | No Hit |
| TTCCTACGGGAGGCTGCAGTGGGGAATATTGGACAATGGGCGAAAGCCTG | 75 | 0.2170138888888889 | No Hit |
| GGCCTACGGGTGGCAGCAGTGGGGAATATTGGACAATGGGCGAAAGCCTG | 74 | 0.21412037037037038 | No Hit |
| TACCTACGGGCGGCAGCAGTGGGGAATATTGGACAATGGGCGAAAGCCTG | 70 | 0.20254629629629628 | No Hit |
| AACCTACGGGGGGCAGCAGTGGGGAATATTGGACAATGGGCGAAAGCCTG | 70 | 0.20254629629629628 | No Hit |
| AACCTACGGGTGGCTGCAGTGGGGAATATTGGACAATGGGCGAAAGCCTG | 69 | 0.19965277777777776 | No Hit |
| TCTACGGGTGGCAGCAGTGGGGAATATTGGACAATGGGCGAAAGCCTGAT | 68 | 0.19675925925925924 | No Hit |
| ACCCTACGGGAGGCAGCAGTGGGGAATATTGGACAATGGGCGAAAGCCTG | 68 | 0.19675925925925924 | No Hit |
| TTCCTACGGGCGGCAGCAGTGGGGAATATTGGACAATGGGCGAAAGCCTG | 67 | 0.19386574074074073 | No Hit |
| GTCCTACGGGGGGCAGCAGTGGGGAATATTGGACAATGGGCGAAAGCCTG | 67 | 0.19386574074074073 | No Hit |
| ATCCTACGGGGGGCAGCAGTGGGGAATATTGGACAATGGGCGAAAGCCTG | 67 | 0.19386574074074073 | No Hit |
| CTCCTACGGGAGGCAGCAGTGGGGAATATTGGACAATGGGCGAAAGCCTG | 67 | 0.19386574074074073 | No Hit |
| CCCTACGGGAGGCTGCAGTGGGGAATATTGGACAATGGGCGAAAGCCTGA | 67 | 0.19386574074074073 | No Hit |
| TATCCTACGGGTGGCAGCAGTGGGGAATATTGGACAATGGGCGAAAGCCT | 66 | 0.1909722222222222 | No Hit |
| GGCCTACGGGAGGCAGCAGTGGGGAATATTGGACAATGGGCGAAAGCCTG | 64 | 0.1851851851851852 | No Hit |
| TTCCCTACGGGTGGCAGCAGTGGGGAATATTGGACAATGGGCGAAAGCCT | 63 | 0.18229166666666666 | No Hit |
| CATACGGGTGGCAGCAGTGGGGAATATTGGACAATGGGCGAAAGCCTGAT | 62 | 0.17939814814814814 | No Hit |
| CACCTACGGGAGGCAGCAGTGGGGAATATTGGACAATGGGCGAAAGCCTG | 62 | 0.17939814814814814 | No Hit |
| CTCCTACGGGTGGCTGCAGTGGGGAATATTGGACAATGGGCGAAAGCCTG | 61 | 0.17650462962962962 | No Hit |
| AGCCTACGGGGGGCAGCAGTGGGGAATATTGGACAATGGGCGAAAGCCTG | 61 | 0.17650462962962962 | No Hit |
| GCCTACGGGGGGCTGCAGTGGGGAATATTGGACAATGGGCGAAAGCCTGA | 61 | 0.17650462962962962 | No Hit |
| GACCTACGGGGGGCAGCAGTGGGGAATATTGGACAATGGGCGAAAGCCTG | 60 | 0.1736111111111111 | No Hit |
| TGCCTACGGGTGGCTGCAGTGGGGAATATTGGACAATGGGCGAAAGCCTG | 59 | 0.1707175925925926 | No Hit |
| ATACCTACGGGTGGCAGCAGTGGGGAATATTGGACAATGGGCGAAAGCCT | 59 | 0.1707175925925926 | No Hit |
| AACCTACGGGAGGCTGCAGTGGGGAATATTGGACAATGGGCGAAAGCCTG | 58 | 0.16782407407407407 | No Hit |
| GACCTACGGGAGGCTGCAGTGGGGAATATTGGACAATGGGCGAAAGCCTG | 55 | 0.15914351851851852 | No Hit |
| TCCCTACGGGTGGCTGCAGTGGGGAATATTGGACAATGGGCGAAAGCCTG | 54 | 0.15625 | No Hit |
| GTCCTACGGGAGGCTGCAGTGGGGAATATTGGACAATGGGCGAAAGCCTG | 54 | 0.15625 | No Hit |
| GTTCCTACGGGTGGCAGCAGTGGGGAATATTGGACAATGGGCGAAAGCCT | 53 | 0.15335648148148148 | No Hit |
| ATCCTACGGGAGGCTGCAGTGGGGAATATTGGACAATGGGCGAAAGCCTG | 53 | 0.15335648148148148 | No Hit |
| TTTTCCTACGGGTGGCAGCAGTGGGGAATATTGGACAATGGGCGAAAGCC | 52 | 0.15046296296296297 | No Hit |
| TGCCTACGGGGGGCAGCAGTGGGGAATATTGGACAATGGGCGAAAGCCTG | 51 | 0.14756944444444445 | No Hit |
| ATCCTACGGGTGGCTGCAGTGGGGAATATTGGACAATGGGCGAAAGCCTG | 50 | 0.14467592592592593 | No Hit |
| CGCCTACGGGTGGCAGCAGTGGGGAATATTGGACAATGGGCGAAAGCCTG | 50 | 0.14467592592592593 | No Hit |
| TACCCTACGGGTGGCAGCAGTGGGGAATATTGGACAATGGGCGAAAGCCT | 50 | 0.14467592592592593 | No Hit |
| TTACCTACGGGAGGCAGCAGTGGGGAATATTGGACAATGGGCGAAAGCCT | 50 | 0.14467592592592593 | No Hit |
| TCCTAAGGGTGGCAGCAGTGGGGAATATTGGACAATGGGCGAAAGCCTGA | 48 | 0.1388888888888889 | No Hit |
| AATCCTACGGGTGGCAGCAGTGGGGAATATTGGACAATGGGCGAAAGCCT | 48 | 0.1388888888888889 | No Hit |
| CCCTACGGGCGGCAGCAGTGGGGAATATTGGACAATGGGCGAAAGCCTGA | 47 | 0.13599537037037038 | No Hit |
| GCCCTACGGGAGGCAGCAGTGGGGAATATTGGACAATGGGCGAAAGCCTG | 46 | 0.13310185185185186 | No Hit |
| TCTACGGGAGGCAGCAGTGGGGAATATTGGACAATGGGCGAAAGCCTGAT | 46 | 0.13310185185185186 | No Hit |
| GTCCTACGGGTGGCTGCAGTGGGGAATATTGGACAATGGGCGAAAGCCTG | 45 | 0.13020833333333331 | No Hit |
| TCCCTACGGGAGGCAGCAGTGGGGAATATTGGACAATGGGCGAAAGCCTG | 45 | 0.13020833333333331 | No Hit |
| CCCCTACGGGTGGCAGCAGTGGGGAATATTGGACAATGGGCGAAAGCCTG | 44 | 0.12731481481481483 | No Hit |
| TTTCCTACGGGAGGCAGCAGTGGGGAATATTGGACAATGGGCGAAAGCCT | 44 | 0.12731481481481483 | No Hit |
| AACCTACGGGCGGCAGCAGTGGGGAATATTGGACAATGGGCGAAAGCCTG | 43 | 0.12442129629629631 | No Hit |
| TCCTACGGGCGGCTGCAGTGGGGAATATTGGACAATGGGCGAAAGCCTGA | 43 | 0.12442129629629631 | No Hit |
| ACCCTACGGGTGGCTGCAGTGGGGAATATTGGACAATGGGCGAAAGCCTG | 42 | 0.12152777777777778 | No Hit |
| ACCTACGGGCGGCTGCAGTGGGGAATATTGGACAATGGGCGAAAGCCTGA | 42 | 0.12152777777777778 | No Hit |
| TGCCTACGGGCGGCAGCAGTGGGGAATATTGGACAATGGGCGAAAGCCTG | 42 | 0.12152777777777778 | No Hit |
| AAACCTACGGGTGGCAGCAGTGGGGAATATTGGACAATGGGCGAAAGCCT | 41 | 0.11863425925925926 | No Hit |
| GAACCTACGGGTGGCAGCAGTGGGGAATATTGGACAATGGGCGAAAGCCT | 41 | 0.11863425925925926 | No Hit |
| TGCCTACGGGAGGCTGCAGTGGGGAATATTGGACAATGGGCGAAAGCCTG | 41 | 0.11863425925925926 | No Hit |
| CACTACGGGTGGCAGCAGTGGGGAATATTGGACAATGGGCGAAAGCCTGA | 41 | 0.11863425925925926 | No Hit |
| TACCTACGGGGGGCTGCAGTGGGGAATATTGGACAATGGGCGAAAGCCTG | 40 | 0.11574074074074073 | No Hit |
| TAGCCTACGGGTGGCAGCAGTGGGGAATATTGGACAATGGGCGAAAGCCT | 40 | 0.11574074074074073 | No Hit |
| ACCCTACGGGAGGCTGCAGTGGGGAATATTGGACAATGGGCGAAAGCCTG | 40 | 0.11574074074074073 | No Hit |
| TGTCCTACGGGTGGCAGCAGTGGGGAATATTGGACAATGGGCGAAAGCCT | 40 | 0.11574074074074073 | No Hit |
| TCCTAAGGGAGGCAGCAGTGGGGAATATTGGACAATGGGCGAAAGCCTGA | 39 | 0.11284722222222221 | No Hit |
| CATACGGGCGGCAGCAGTGGGGAATATTGGACAATGGGCGAAAGCCTGAT | 39 | 0.11284722222222221 | No Hit |
| GGCCTACGGGGGGCAGCAGTGGGGAATATTGGACAATGGGCGAAAGCCTG | 39 | 0.11284722222222221 | No Hit |
| TTTCCTACGGGGGGCAGCAGTGGGGAATATTGGACAATGGGCGAAAGCCT | 39 | 0.11284722222222221 | No Hit |
| CACCTACGGGTGGCTGCAGTGGGGAATATTGGACAATGGGCGAAAGCCTG | 38 | 0.1099537037037037 | No Hit |
| TATCCTACGGGAGGCAGCAGTGGGGAATATTGGACAATGGGCGAAAGCCT | 38 | 0.1099537037037037 | No Hit |
| GCCTACGGGCGGCTGCAGTGGGGAATATTGGACAATGGGCGAAAGCCTGA | 38 | 0.1099537037037037 | No Hit |
| ATTCCTACGGGGGGCAGCAGTGGGGAATATTGGACAATGGGCGAAAGCCT | 37 | 0.10706018518518519 | No Hit |
| TTGCCTACGGGTGGCAGCAGTGGGGAATATTGGACAATGGGCGAAAGCCT | 37 | 0.10706018518518519 | No Hit |
| AGCCTACGGGAGGCTGCAGTGGGGAATATTGGACAATGGGCGAAAGCCTG | 37 | 0.10706018518518519 | No Hit |
| AGCCTACGGGTGGCTGCAGTGGGGAATATTGGACAATGGGCGAAAGCCTG | 36 | 0.10416666666666667 | No Hit |
| ATCCCTACGGGTGGCAGCAGTGGGGAATATTGGACAATGGGCGAAAGCCT | 36 | 0.10416666666666667 | No Hit |
| GCCCTACGGGTGGCTGCAGTGGGGAATATTGGACAATGGGCGAAAGCCTG | 36 | 0.10416666666666667 | No Hit |
| AGACCTACGGGTGGCAGCAGTGGGGAATATTGGACAATGGGCGAAAGCCT | 35 | 0.10127314814814814 | No Hit |

## Adapter Content

Produced by FastQC (version 0.12.1)
