# Supplementary material for: Bacterial community data associated with Stephanitis pyri using 16S rRNA gene metabarcoding
Source: Data Brief. 2026 Jul 29;68:113107. doi: 10.1016/j.dib.2026.113107 (PMC13474393; doi:10.1016/j.dib.2026.113107)
Supplement: Supplementary file 1 [file mmc1.zip › supplementary_material_1/fastqc/Extr-N4063-16Sv3v4_S53_L001_R2_001_fastqc.zip!/Extr-N4063-16Sv3v4_S53_L001_R2_001_fastqc/fastqc_report.html]

Extr-N4063-16Sv3v4\_S53\_L001\_R2\_001.fastq.gz FastQC Report 

FastQC Report

Mon 13 Oct 2025  
Extr-N4063-16Sv3v4\_S53\_L001\_R2\_001.fastq.gz

## Summary

- Basic Statistics
- Per base sequence quality
- Per tile sequence quality
- Per sequence quality scores
- Per base sequence content
- Per sequence GC content
- Per base N content
- Sequence Length Distribution
- Sequence Duplication Levels
- Overrepresented sequences
- Adapter Content

## Basic Statistics

| Measure | Value |
| --- | --- |
| Filename | Extr-N4063-16Sv3v4\_S53\_L001\_R2\_001.fastq.gz |
| File type | Conventional base calls |
| Encoding | Sanger / Illumina 1.9 |
| Total Sequences | 34560 |
| Total Bases | 10.4 Mbp |
| Sequences flagged as poor quality | 0 |
| Sequence length | 301 |
| %GC | 52 |

## Per base sequence quality

## Per tile sequence quality

## Per sequence quality scores

## Per base sequence content

## Per sequence GC content

## Per base N content

## Sequence Length Distribution

## Sequence Duplication Levels

## Overrepresented sequences

| Sequence | Count | Percentage | Possible Source |
| --- | --- | --- | --- |
| GACTACTAGGGTATCTAATCCTGTTTGCTCCCCACGCTTTCGCACCTCAG | 1024 | 2.9629629629629632 | No Hit |
| GACTACTCGGGTATCTAATCCTGTTTGCTCCCCACGCTTTCGCACCTCAG | 871 | 2.5202546296296298 | No Hit |
| GACTACTGGGGTATCTAATCCTGTTTGCTCCCCACGCTTTCGCACCTCAG | 804 | 2.326388888888889 | No Hit |
| GACTACAAGGGTATCTAATCCTGTTTGCTCCCCACGCTTTCGCACCTCAG | 802 | 2.3206018518518516 | No Hit |
| GACTACCAGGGTATCTAATCCTGTTTGCTCCCCACGCTTTCGCACCTCAG | 656 | 1.8981481481481481 | No Hit |
| GACTACACGGGTATCTAATCCTGTTTGCTCCCCACGCTTTCGCACCTCAG | 641 | 1.8547453703703705 | No Hit |
| GACTACAGGGGTATCTAATCCTGTTTGCTCCCCACGCTTTCGCACCTCAG | 548 | 1.5856481481481481 | No Hit |
| GACTACCGGGGTATCTAATCCTGTTTGCTCCCCACGCTTTCGCACCTCAG | 430 | 1.244212962962963 | No Hit |
| GACTACCCGGGTATCTAATCCTGTTTGCTCCCCACGCTTTCGCACCTCAG | 417 | 1.2065972222222223 | No Hit |
| TGACTACTAGGGTATCTAATCCTGTTTGCTCCCCACGCTTTCGCACCTCA | 357 | 1.0329861111111112 | No Hit |
| TGACTACTCGGGTATCTAATCCTGTTTGCTCCCCACGCTTTCGCACCTCA | 310 | 0.8969907407407407 | No Hit |
| TGACTACAAGGGTATCTAATCCTGTTTGCTCCCCACGCTTTCGCACCTCA | 274 | 0.7928240740740741 | No Hit |
| TGACTACCAGGGTATCTAATCCTGTTTGCTCCCCACGCTTTCGCACCTCA | 248 | 0.7175925925925926 | No Hit |
| GGACTACTAGGGTATCTAATCCTGTTTGCTCCCCACGCTTTCGCACCTCA | 221 | 0.6394675925925926 | No Hit |
| TGACTACTGGGGTATCTAATCCTGTTTGCTCCCCACGCTTTCGCACCTCA | 218 | 0.6307870370370371 | No Hit |
| AGACTACTAGGGTATCTAATCCTGTTTGCTCCCCACGCTTTCGCACCTCA | 211 | 0.6105324074074073 | No Hit |
| CGACTACTAGGGTATCTAATCCTGTTTGCTCCCCACGCTTTCGCACCTCA | 199 | 0.5758101851851852 | No Hit |
| TGACTACAGGGGTATCTAATCCTGTTTGCTCCCCACGCTTTCGCACCTCA | 194 | 0.5613425925925927 | No Hit |
| AGACTACTCGGGTATCTAATCCTGTTTGCTCCCCACGCTTTCGCACCTCA | 181 | 0.5237268518518519 | No Hit |
| CGACTACAAGGGTATCTAATCCTGTTTGCTCCCCACGCTTTCGCACCTCA | 177 | 0.5121527777777778 | No Hit |
| GGACTACTCGGGTATCTAATCCTGTTTGCTCCCCACGCTTTCGCACCTCA | 175 | 0.5063657407407407 | No Hit |
| AGACTACAAGGGTATCTAATCCTGTTTGCTCCCCACGCTTTCGCACCTCA | 174 | 0.5034722222222222 | No Hit |
| GGACTACTGGGGTATCTAATCCTGTTTGCTCCCCACGCTTTCGCACCTCA | 171 | 0.49479166666666663 | No Hit |
| CGACTACTGGGGTATCTAATCCTGTTTGCTCCCCACGCTTTCGCACCTCA | 171 | 0.49479166666666663 | No Hit |
| GGACTACAAGGGTATCTAATCCTGTTTGCTCCCCACGCTTTCGCACCTCA | 168 | 0.4861111111111111 | No Hit |
| TGACTACCGGGGTATCTAATCCTGTTTGCTCCCCACGCTTTCGCACCTCA | 168 | 0.4861111111111111 | No Hit |
| TGACTACCCGGGTATCTAATCCTGTTTGCTCCCCACGCTTTCGCACCTCA | 166 | 0.48032407407407407 | No Hit |
| CGACTACTCGGGTATCTAATCCTGTTTGCTCCCCACGCTTTCGCACCTCA | 154 | 0.44560185185185186 | No Hit |
| GGACTACCAGGGTATCTAATCCTGTTTGCTCCCCACGCTTTCGCACCTCA | 150 | 0.4340277777777778 | No Hit |
| AGACTACTGGGGTATCTAATCCTGTTTGCTCCCCACGCTTTCGCACCTCA | 150 | 0.4340277777777778 | No Hit |
| AGACTACACGGGTATCTAATCCTGTTTGCTCCCCACGCTTTCGCACCTCA | 140 | 0.40509259259259256 | No Hit |
| AGACTACCAGGGTATCTAATCCTGTTTGCTCCCCACGCTTTCGCACCTCA | 138 | 0.3993055555555555 | No Hit |
| GGACTACAGGGGTATCTAATCCTGTTTGCTCCCCACGCTTTCGCACCTCA | 137 | 0.39641203703703703 | No Hit |
| AGACTACAGGGGTATCTAATCCTGTTTGCTCCCCACGCTTTCGCACCTCA | 136 | 0.3935185185185185 | No Hit |
| TGACTACACGGGTATCTAATCCTGTTTGCTCCCCACGCTTTCGCACCTCA | 133 | 0.38483796296296297 | No Hit |
| CGACTACCAGGGTATCTAATCCTGTTTGCTCCCCACGCTTTCGCACCTCA | 126 | 0.3645833333333333 | No Hit |
| TTGACTACTAGGGTATCTAATCCTGTTTGCTCCCCACGCTTTCGCACCTC | 125 | 0.3616898148148148 | No Hit |
| CGACTACAGGGGTATCTAATCCTGTTTGCTCCCCACGCTTTCGCACCTCA | 113 | 0.32696759259259256 | No Hit |
| AGACTACCGGGGTATCTAATCCTGTTTGCTCCCCACGCTTTCGCACCTCA | 109 | 0.31539351851851855 | No Hit |
| GGACTACCGGGGTATCTAATCCTGTTTGCTCCCCACGCTTTCGCACCTCA | 108 | 0.3125 | No Hit |
| TTGACTACTCGGGTATCTAATCCTGTTTGCTCCCCACGCTTTCGCACCTC | 106 | 0.30671296296296297 | No Hit |
| GGACTACACGGGTATCTAATCCTGTTTGCTCCCCACGCTTTCGCACCTCA | 104 | 0.30092592592592593 | No Hit |
| CGACTACACGGGTATCTAATCCTGTTTGCTCCCCACGCTTTCGCACCTCA | 97 | 0.28067129629629634 | No Hit |
| GTGACTACTAGGGTATCTAATCCTGTTTGCTCCCCACGCTTTCGCACCTC | 96 | 0.2777777777777778 | No Hit |
| TTGACTACCAGGGTATCTAATCCTGTTTGCTCCCCACGCTTTCGCACCTC | 93 | 0.2690972222222222 | No Hit |
| ATGACTACTCGGGTATCTAATCCTGTTTGCTCCCCACGCTTTCGCACCTC | 88 | 0.25462962962962965 | No Hit |
| AGACTACCCGGGTATCTAATCCTGTTTGCTCCCCACGCTTTCGCACCTCA | 83 | 0.24016203703703703 | No Hit |
| CGACTACCCGGGTATCTAATCCTGTTTGCTCCCCACGCTTTCGCACCTCA | 76 | 0.2199074074074074 | No Hit |
| TCGACTACTAGGGTATCTAATCCTGTTTGCTCCCCACGCTTTCGCACCTC | 75 | 0.2170138888888889 | No Hit |
| TGGACTACTAGGGTATCTAATCCTGTTTGCTCCCCACGCTTTCGCACCTC | 75 | 0.2170138888888889 | No Hit |
| ATGACTACTGGGGTATCTAATCCTGTTTGCTCCCCACGCTTTCGCACCTC | 74 | 0.21412037037037038 | No Hit |
| TTGACTACTGGGGTATCTAATCCTGTTTGCTCCCCACGCTTTCGCACCTC | 74 | 0.21412037037037038 | No Hit |
| TAGACTACTCGGGTATCTAATCCTGTTTGCTCCCCACGCTTTCGCACCTC | 73 | 0.21122685185185186 | No Hit |
| ATGACTACTAGGGTATCTAATCCTGTTTGCTCCCCACGCTTTCGCACCTC | 72 | 0.20833333333333334 | No Hit |
| ATGACTACAAGGGTATCTAATCCTGTTTGCTCCCCACGCTTTCGCACCTC | 70 | 0.20254629629629628 | No Hit |
| CTGACTACTAGGGTATCTAATCCTGTTTGCTCCCCACGCTTTCGCACCTC | 65 | 0.1880787037037037 | No Hit |
| TAGACTACTAGGGTATCTAATCCTGTTTGCTCCCCACGCTTTCGCACCTC | 63 | 0.18229166666666666 | No Hit |
| GTGACTACAAGGGTATCTAATCCTGTTTGCTCCCCACGCTTTCGCACCTC | 61 | 0.17650462962962962 | No Hit |
| GGGGGGGGGGGGGGGGGGGGGGGGGGGGGGGGGGGGGGGGGGGGGGGGGG | 61 | 0.17650462962962962 | No Hit |
| TTGACTACAAGGGTATCTAATCCTGTTTGCTCCCCACGCTTTCGCACCTC | 60 | 0.1736111111111111 | No Hit |
| TGGACTACTCGGGTATCTAATCCTGTTTGCTCCCCACGCTTTCGCACCTC | 58 | 0.16782407407407407 | No Hit |
| TGGACTACTGGGGTATCTAATCCTGTTTGCTCCCCACGCTTTCGCACCTC | 58 | 0.16782407407407407 | No Hit |
| TTGACTACCGGGGTATCTAATCCTGTTTGCTCCCCACGCTTTCGCACCTC | 57 | 0.16493055555555555 | No Hit |
| TTGACTACACGGGTATCTAATCCTGTTTGCTCCCCACGCTTTCGCACCTC | 56 | 0.16203703703703703 | No Hit |
| CTGACTACTGGGGTATCTAATCCTGTTTGCTCCCCACGCTTTCGCACCTC | 56 | 0.16203703703703703 | No Hit |
| TAGACTACAGGGGTATCTAATCCTGTTTGCTCCCCACGCTTTCGCACCTC | 56 | 0.16203703703703703 | No Hit |
| CTGACTACTCGGGTATCTAATCCTGTTTGCTCCCCACGCTTTCGCACCTC | 56 | 0.16203703703703703 | No Hit |
| TAGACTACCAGGGTATCTAATCCTGTTTGCTCCCCACGCTTTCGCACCTC | 54 | 0.15625 | No Hit |
| TGGACTACAAGGGTATCTAATCCTGTTTGCTCCCCACGCTTTCGCACCTC | 53 | 0.15335648148148148 | No Hit |
| TAGACTACTGGGGTATCTAATCCTGTTTGCTCCCCACGCTTTCGCACCTC | 53 | 0.15335648148148148 | No Hit |
| GTGACTACTCGGGTATCTAATCCTGTTTGCTCCCCACGCTTTCGCACCTC | 53 | 0.15335648148148148 | No Hit |
| GTGACTACTGGGGTATCTAATCCTGTTTGCTCCCCACGCTTTCGCACCTC | 53 | 0.15335648148148148 | No Hit |
| CGGACTACTAGGGTATCTAATCCTGTTTGCTCCCCACGCTTTCGCACCTC | 53 | 0.15335648148148148 | No Hit |
| ACGACTACTAGGGTATCTAATCCTGTTTGCTCCCCACGCTTTCGCACCTC | 52 | 0.15046296296296297 | No Hit |
| TTGACTACCCGGGTATCTAATCCTGTTTGCTCCCCACGCTTTCGCACCTC | 52 | 0.15046296296296297 | No Hit |
| GAGACTACTCGGGTATCTAATCCTGTTTGCTCCCCACGCTTTCGCACCTC | 52 | 0.15046296296296297 | No Hit |
| AAGACTACTGGGGTATCTAATCCTGTTTGCTCCCCACGCTTTCGCACCTC | 51 | 0.14756944444444445 | No Hit |
| AGGACTACTAGGGTATCTAATCCTGTTTGCTCCCCACGCTTTCGCACCTC | 50 | 0.14467592592592593 | No Hit |
| TAGACTACACGGGTATCTAATCCTGTTTGCTCCCCACGCTTTCGCACCTC | 50 | 0.14467592592592593 | No Hit |
| GCGACTACTAGGGTATCTAATCCTGTTTGCTCCCCACGCTTTCGCACCTC | 50 | 0.14467592592592593 | No Hit |
| GGACTACCCGGGTATCTAATCCTGTTTGCTCCCCACGCTTTCGCACCTCA | 49 | 0.1417824074074074 | No Hit |
| CTGACTACAAGGGTATCTAATCCTGTTTGCTCCCCACGCTTTCGCACCTC | 49 | 0.1417824074074074 | No Hit |
| TTGACTACAGGGGTATCTAATCCTGTTTGCTCCCCACGCTTTCGCACCTC | 49 | 0.1417824074074074 | No Hit |
| ATGACTACCAGGGTATCTAATCCTGTTTGCTCCCCACGCTTTCGCACCTC | 47 | 0.13599537037037038 | No Hit |
| AAGACTACTAGGGTATCTAATCCTGTTTGCTCCCCACGCTTTCGCACCTC | 47 | 0.13599537037037038 | No Hit |
| TCGACTACTGGGGTATCTAATCCTGTTTGCTCCCCACGCTTTCGCACCTC | 47 | 0.13599537037037038 | No Hit |
| GTGACTACAGGGGTATCTAATCCTGTTTGCTCCCCACGCTTTCGCACCTC | 47 | 0.13599537037037038 | No Hit |
| GGGACTACTCGGGTATCTAATCCTGTTTGCTCCCCACGCTTTCGCACCTC | 46 | 0.13310185185185186 | No Hit |
| ACGACTACTCGGGTATCTAATCCTGTTTGCTCCCCACGCTTTCGCACCTC | 46 | 0.13310185185185186 | No Hit |
| GGGACTACTAGGGTATCTAATCCTGTTTGCTCCCCACGCTTTCGCACCTC | 46 | 0.13310185185185186 | No Hit |
| CAGACTACTAGGGTATCTAATCCTGTTTGCTCCCCACGCTTTCGCACCTC | 45 | 0.13020833333333331 | No Hit |
| ACGACTACCAGGGTATCTAATCCTGTTTGCTCCCCACGCTTTCGCACCTC | 45 | 0.13020833333333331 | No Hit |
| CTGACTACCAGGGTATCTAATCCTGTTTGCTCCCCACGCTTTCGCACCTC | 45 | 0.13020833333333331 | No Hit |
| GAGACTACTAGGGTATCTAATCCTGTTTGCTCCCCACGCTTTCGCACCTC | 45 | 0.13020833333333331 | No Hit |
| CAGACTACAAGGGTATCTAATCCTGTTTGCTCCCCACGCTTTCGCACCTC | 45 | 0.13020833333333331 | No Hit |
| TAGACTACCCGGGTATCTAATCCTGTTTGCTCCCCACGCTTTCGCACCTC | 44 | 0.12731481481481483 | No Hit |
| TCGACTACCAGGGTATCTAATCCTGTTTGCTCCCCACGCTTTCGCACCTC | 44 | 0.12731481481481483 | No Hit |
| ATGACTACACGGGTATCTAATCCTGTTTGCTCCCCACGCTTTCGCACCTC | 43 | 0.12442129629629631 | No Hit |
| TTTGACTACTAGGGTATCTAATCCTGTTTGCTCCCCACGCTTTCGCACCT | 43 | 0.12442129629629631 | No Hit |
| AAGACTACTCGGGTATCTAATCCTGTTTGCTCCCCACGCTTTCGCACCTC | 43 | 0.12442129629629631 | No Hit |
| TAGACTACAAGGGTATCTAATCCTGTTTGCTCCCCACGCTTTCGCACCTC | 42 | 0.12152777777777778 | No Hit |
| TTTGACTACTCGGGTATCTAATCCTGTTTGCTCCCCACGCTTTCGCACCT | 41 | 0.11863425925925926 | No Hit |
| TCGACTACAAGGGTATCTAATCCTGTTTGCTCCCCACGCTTTCGCACCTC | 40 | 0.11574074074074073 | No Hit |
| ATGACTACCGGGGTATCTAATCCTGTTTGCTCCCCACGCTTTCGCACCTC | 39 | 0.11284722222222221 | No Hit |
| CGGACTACAAGGGTATCTAATCCTGTTTGCTCCCCACGCTTTCGCACCTC | 39 | 0.11284722222222221 | No Hit |
| CGACTACCGGGGTATCTAATCCTGTTTGCTCCCCACGCTTTCGCACCTCA | 38 | 0.1099537037037037 | No Hit |
| TCGACTACTCGGGTATCTAATCCTGTTTGCTCCCCACGCTTTCGCACCTC | 38 | 0.1099537037037037 | No Hit |
| CAGACTACTCGGGTATCTAATCCTGTTTGCTCCCCACGCTTTCGCACCTC | 38 | 0.1099537037037037 | No Hit |
| TTAGACTACTAGGGTATCTAATCCTGTTTGCTCCCCACGCTTTCGCACCT | 38 | 0.1099537037037037 | No Hit |
| GCGACTACTGGGGTATCTAATCCTGTTTGCTCCCCACGCTTTCGCACCTC | 37 | 0.10706018518518519 | No Hit |
| GGGACTACTGGGGTATCTAATCCTGTTTGCTCCCCACGCTTTCGCACCTC | 37 | 0.10706018518518519 | No Hit |
| TAGACTACCGGGGTATCTAATCCTGTTTGCTCCCCACGCTTTCGCACCTC | 37 | 0.10706018518518519 | No Hit |
| AAGACTACAAGGGTATCTAATCCTGTTTGCTCCCCACGCTTTCGCACCTC | 37 | 0.10706018518518519 | No Hit |
| TGGACTACAGGGGTATCTAATCCTGTTTGCTCCCCACGCTTTCGCACCTC | 36 | 0.10416666666666667 | No Hit |
| GGGACTACAAGGGTATCTAATCCTGTTTGCTCCCCACGCTTTCGCACCTC | 36 | 0.10416666666666667 | No Hit |
| GACTAACAGGGTATCTAATCCTGTTTGCTCCCCACGCTTTCGCACCTCAG | 36 | 0.10416666666666667 | No Hit |
| AGGACTACTGGGGTATCTAATCCTGTTTGCTCCCCACGCTTTCGCACCTC | 35 | 0.10127314814814814 | No Hit |
| CTGACTACACGGGTATCTAATCCTGTTTGCTCCCCACGCTTTCGCACCTC | 35 | 0.10127314814814814 | No Hit |

## Adapter Content

Produced by FastQC (version 0.12.1)
