# Supplementary material for: Bacterial community data associated with Stephanitis pyri using 16S rRNA gene metabarcoding
Source: Data Brief. 2026 Jul 29;68:113107. doi: 10.1016/j.dib.2026.113107 (PMC13474393; doi:10.1016/j.dib.2026.113107)
Supplement: Supplementary file 2 [file mmc2.zip › supplementary_material_2/qiime2_results/filtered/exported_files/krona/index.html]

Javascript must be enabled to view this page.

magnitude
magnitudeUnassigned

c1
c2
c3
c4
Extr

615116633264186374501236

4558659322

4558659322

4558659322

4558659322

4558659322

4558659322

4558659322

1506106

00066

00066

00066

00066

00066

00066

150640

150640

150640

150640

150640

150640

015000

015000

015000

015000

015000

015000

015000

614516625964115373471208

16154206

16154206

16154206

16154206

16154206

16154206

04905

04905

04905

04905

04905

04905

130000

130000

130000

130000

130000

130000

6041448566450

180000

180000

180000

180000

180000

000011

000011

000011

000011

000011

332520023

181020023

181020023

181020023

181020023

1515000

1515000

1515000

1515000

06600

06600

06600

06600

06600

1301600

1301600

1301600

1301600

1301600

527864361342

21121018101

21121018101

912101127

912101127

000016

000016

1200758

000726

1200032

0562820157

000044

000044

000044

0562820113

0562820103

000012

049182070

070021

001000

000010

000010

000012

000012

000012

000012

2370025

200025

200025

200025

150000

150000

150000

2020000

2020000

480000

1540000

2691801520

2691801520

2691801520

2691801520

000014

000014

000014

000014

005633

005633

000012

000012

005621

005621

13270574

1080558

1080558

1080558

000010

1080548

3190016

3190016

3190016

3190016

120000

120000

120000

120000

120000

120000

0311029

0311012

0311012

030012

030012

030012

001100

001100

001100

000017

000017

000017

000017

000017

58228643086215636064295

57279641146203435325159

266818079

160000

160000

160000

465500

465500

465500

2130022

2100010

2100010

030012

030012

183013046

14501300

14501300

1900018

1900018

000011

000011

000017

000017

190000

190000

000011

000011

000011

2000036

2000036

000013

000013

000023

000023

200000

200000

5692264097620103532515

5692264097620103532515

5692264097620103532515

5692264097620103532515

385600

385600

280000

280000

105600

105600

000011

000011

000011

000011

000011

000011

000011

000011

334007

334007

334007

334007

949194122739136

000010

000010

000010

000010

86310562

86310562

5610050

5610050

9210045

9210045

70007

70007

1400010

1400010

25542240925

11403785

500400

500400

6403385

6002605

040780

2440223120

2440223120

2440223120

0244027

0244027

0244027

0244027

2971950

2971950

1801950

1801950

117000

117000

393101771512

300000

200000

200000

100000

100000

170000

170000

170000

2796877150

2526877150

2526877150

160000

160000

110000

110000

3600012

000012

000012

360000

360000

522000

017000

017000

55000

55000

2611000

260000

260000

011000

011000

170000

170000

170000

170000

6216000

6216000

100000

100000

5216000

5216000

1071103050

01102740

000400

000400

01102340

01102340

1070000

1070000

1070000

000310

000310

000310

68866020

6251600

6251600

016000

016000

016000

5135600

5135600

510000

035600

110000

110000

110000

6350020

040020

040020

040020

040020

621000

621000

621000

68000

013000

010000

010000

010000

010000

503017

000010

000010

000010

000010

000010

50307

50307

50307

50307

50307

1115274253189175

000011

000011

000011

000011

000011

000025

000015

000015

000015

000015

000010

000010

000010

000010

1115274253189139

3021761651370

3021761651370

3021761651370

3021761651370

180000

180000

180000

180000

10235022

10235022

0125022

0125022

1011000

1011000

000015

000015

000015

000015

12924363553

050011

050011

050011

1000133527

1000133527

1000133527

131923015

130000

130000

01923015

01923015

160000

160000

160000

240000

120000

120000

120000

120000

120000

120000

1548261718

6408018

4508018

190000

150000

1108018

190000

190000

7261870

4161870

4161870

310000

310000

1820100

1820100

1820100

4744316011

428117011

428117011

428117011

4632900

3512900

3512900

110000

110000

020000

020000

405020

405020

405020

405020

1173178091

1173178091

2551900

195900

195900

195900

601000

601000

601000

37018036

4018020

0011020

0011020

40700

40700

3300016

1900016

1900016

140000

140000

481041018

360200

360200

260200

100000

0019018

0019018

0019018

002000

002000

002000

1210000

1210000

1210000

75000

75000

75000

75000

0110037

0110037

0110023

0110023

000014

000014

70900

70900

70900

70900

70900

70900

0100011

010000

010000

010000

010000

010000

000011

000011

000011

000011

000011

124813661453102310

124813661453102310

124813661453102310

124813661453102310

124813661453102310

124813661453102310

000010

000010

000010

000010

000010

000010

0100014

000014

000014

000014

000014

000014

010000

010000

010000

010000

010000

000029

000010

000010

000010

000010

000010

000019

000019

000019

000019

000019

18010535

18010535

0010011

000011

000011

000011

001000

001000

001000

1800524

1800524

180055

80055

100000

000019

000019

080011

080011

080011

080011

080011

080011
