# Supplementary material for: Bacterial community data associated with Stephanitis pyri using 16S rRNA gene metabarcoding
Source: Data Brief. 2026 Jul 29;68:113107. doi: 10.1016/j.dib.2026.113107 (PMC13474393; doi:10.1016/j.dib.2026.113107)
Supplement: Supplementary file 2 [file mmc2.zip › supplementary_material_2/qiime2_results/filtered/exported_files/alpha_rarefaction/index.html]

q2\_diversity : alpha\_rarefaction


# Alpha rarefaction

Download CSV

Metric

Sample Metadata Column

Help

×

#### Alpha Diversity Help

In the legend below, click the box symbols to toggle the
display of the line charts and click the circles to toggle
the display of the box plots and scatter plots. If a color is
visible in the symbol, the corresponding plot elements are
displayed, otherwise they are hidden.

The box plots in the upper figure represent the distribution
of the selected alpha diversity metric for each group of
samples at each even sampling depth. The lower and upper
whiskers of the box plot are the 9th and 91st percentiles of
the distribution (respectively), while the lower and upper
extents of the box are the 25th and 75th percentiles of the
distribution (respectively). The horizontal bar through the
middle of the box is the median of the dstribution
(i.e., the 50th percentile). Outlier points of these
distributions are not shown.

The line chart in the upper figure connects the median
values of the alpha diversity metric distribution
across the sampling depths.

If a sampling depth is higher than the number of sequences
in a sample, that sample will not be included in the
rarefaction plot at that sampling depth. The line chart in
the lower figure illustrates the number of samples in each
group (i.e., the sample size for each box plot) at each
sampling depth.

Close
